# Prediction of the axial compression capacity of stub CFST columns using machine learning techniques

**DOI:** 10.1038/s41598-024-53352-1

**Published:** 2024-02-05

**Authors:** Khaled Megahed, Nabil Said Mahmoud, Saad Elden Mostafa Abd-Rabou

**Affiliations:** Department of Structural Engineering, Mansoura University, Mansoura, Egypt

**Keywords:** Materials science, Computer science, Scientific data, Statistics

## Abstract

Concrete-filled steel tubular (CFST) columns have extensive applications in structural engineering due to their exceptional load-bearing capability and ductility. However, existing design code standards often yield different design capacities for the same column properties, introducing uncertainty for engineering designers. Moreover, conventional regression analysis fails to accurately predict the intricate relationship between column properties and compressive strength. To address these issues, this study proposes the use of two machine learning (ML) models—Gaussian process regression (GPR) and symbolic regression (SR). These models accept a variety of input variables, encompassing geometric and material properties of stub CFST columns, to estimate their strength. An experimental database of 1316 specimens was compiled from various research papers, including circular, rectangular, and double-skin stub CFST columns. In addition, a dimensionless output variable, referred to as the strength index, is introduced to enhance model performance. To validate the efficiency of the introduced models, predictions from these models are compared with those from two established standard codes and various ML algorithms, including support vector regression optimized with particle swarm optimization (PSVR), artificial neural networks, XGBoost (XGB), CatBoost (CATB), Random Forest, and LightGBM models. Through performance metrics, the CATB, GPR, PSVR and XGB models emerge as the most accurate and reliable models from the evaluation results. In addition, simple and practical design equations for the different types of CFST columns have been proposed based on the SR model. The developed ML models and proposed equations can predict the compressive strength of stub CFST columns with reliable and accurate results, making them valuable tools for structural engineering. Furthermore, the Shapley additive interpretation (SHAP) technique is employed for feature analysis. The results of the feature analysis reveal that section slenderness ratio and concrete strength parameters negatively impact the compressive strength index.

## Introduction

Concrete-filled steel tube (CFST) members are composite structures of hollow steel tubes filled with concrete. They offer advantages over steel and traditional reinforced concrete columns, including improved structural performance, strength, and ductility^1^. The steel tube provides confinement to the concrete core, delaying or preventing its failure or lateral expansion, and the concrete core constrains the inward local buckling of the outer steel tube^1,2^. CFST columns also exhibit enhanced fire and seismic resistance and act as permanent formwork for concrete, reducing construction time.

CFST columns are available in different types based on loading patterns and geometry, such as concrete-filled steel tube columns and concrete-filled double-skin steel tubular columns (CFDSTs), which have an additional hollow inner steel tube arrangement. Different cross-sectional shapes can be used for CFST columns^3–6^, including circular, square, rectangular, octagonal, hexagonal, or elliptic sections. Circular CFST columns are preferred for their uniform confinement on the concrete core, providing higher load-bearing capacity and ductility. In contrast, rectangular-shaped columns are preferred for the ease of construction and connection erection.

In addition, CFST columns are classified in terms of global buckling as stub and long columns. Stub columns, also known as short columns, are designed to resist axial compressive loads and have relatively short heights compared to their cross-section dimensions^7,8^. The design considerations for stub and long columns differ due to their distinct structural behaviors, i.e., stub columns need to investigate the interaction between confined concrete and the steel tube and local outward buckling. On the other hand, long columns may need more attention to factors such as effective length, global buckling, and lateral bracing to consider stability issues.

Experimental investigations are commonly used to investigate the behavior of CFST columns. However, experimental studies are often limited by the range of parameters and can be costly and time-consuming. Machine learning (ML) techniques can complement experimental studies, as they have proven effective in predicting structural element behaviors. ML algorithms such as support vector regression (SVR)^9^, Gaussian process (GPR)^10,11^, gene expression programming (GEP)^12^, and artificial neural network (ANN)^13–18^ have been developed and successfully used by researchers in developing empirical formulas and statistical models for predicting material properties such as strength and elastic modulus, as well as the performance of structural members.

ML methods find numerous applications in predicting the ultimate capacity of CFST columns, with artificial neural networks (ANNs) commonly employed. For example, Ahmadi et al.^13,14^ utilized ANN to predict the ultimate strength of short CFST columns. Du et al.^15^ employed ANN models to calculate the axial concentric strength of stub rectangular CFST columns using 305 column specimens. Le et al.^16^ used an ANN to predict the axial capacity of square and rectangular CFST columns using 880 specimens. Tran et al.^17^ gathered a database of 300 samples under uniaxial loading to drive ML models for calculating the axial strength of the squared CFT column. In addition, Zarringol et al.^18^ utilized four distinct databases, totaling 3091 CFST columns, encompassing rectangular and circular columns with and without eccentricity. They developed four separate ANN models for the axial capacities of each category and incorporated strength reduction factors to enhance practical design applications.

Gaussian process regression (GPR) is another ML technique applied in computing the ultimate capacity of CFST columns. Le^19^ proposed a GPR-based ML model for estimating the ultimate strength of square CFSTs, demonstrating a considerably high prediction accuracy. Furthermore, Hou and Zhou^20^ optimized ML models, including the backpropagation ANN, GPR model, genetic algorithm, radial basis function neural network (RBFNN), and multiple linear regression (MLR) models, to predict the axial compressive strength of stub and long circular CFST columns.

Furthermore, gene expression programming (GEP) and genetic algorithms (GAs)^12^ are valuable tools in predicting empirical formulas for the ultimate strength of CFST columns. Guneyisi et al.^21,22^ utilized gene expression programming to generate empirical formulations for the axial strength of circular CFST and CFDST stub columns. Javed et al.^23^ implemented GEP to predict the load-bearing strength of circular CFST long columns. Furthermore, Jiang et al.^24^ compared GEP results and finite element analysis outcomes for circular CFST columns. Naser et al.^25^ employed GA and GEP to develop predictive models for the axial ultimate load of rectangular and circular CFST columns. Table 1 summarizes previous ML models in predicting CFST stub column strength.

**Table 1:** Summary of previous ML models in predicting the strength of axially loaded stub CFST columns.

Reference	Category (number) Type [Split ratio%]	Input (output)	Models: Statistical criteria
Ahmadi ^13,14^	Stub (272) Circular [80:20]	*L, D, t, f*_*y*_*, f*_*c*_*′, E*_*s*_ (*P*_*u*_)	ANN: R^2^ = 0.97, MAPE% = 5.8, Regression model: R^2^ = 0.926, MAPE% = 13.2, with expression ${P}_{u}=p\left(D\right)p\left({f}_{y}\right)p\left({f}_{c}{\prime}\right)p\left(t\right)p\left(L\right)$ where $p(x)$ is a third-degree to six-degree polynomial function of x
Du^15^	Stub (305) Rectangular [90:10]	*H, B, t, f*_*y*_*, f*_*c*_*′* (*P*_*u*_)	ANN: μ = 1.013, CoV = 0.0702
Le^16^	Stub + long (880) Rectangular [83:17]	*L, H, B, t, f*_*y*_*, f*_*c*_*′, E*_*s*_ (*P*_*u*_)	ANN: R^2^ = 0.9956, a20-index = 0.925, RMSE = 154.66, MAPE% = 7.54
Tran^41^	Stub + long (258) Circular [85:15]	*L, D, t, f*_*y*_*, f*_*c*_*′* (*P*_*u*_)	Regression model: with expression ${P}_{u}=p\left(D\right)p\left({f}_{y}\right)p\left({f}_{c}{\prime}\right)p\left(t\right)p\left(L\right)$ where $p(x)$ is a polynomial function of x. μ = 1.04, CoV = 0.24, MAPE = 0.18, MAE = 323.52, RMSE = 565, R^2^ = 0.95
Tran^17^	Stub + long (300) Square [85:15]	*L, H, t, f*_*y*_*, f*_*c*_*′* (*P*_*u*_)	ANN: μ = 1.05, CoV = 0.07, R^2^ = 0.996, a20-index = 0.993, MSE = 0.011535 Regression model: with expression ${P}_{u}=p\left(H\right)p\left({f}_{y}\right)p\left({f}_{c}{\prime}\right)p\left(t\right)p\left(L\right)$ where $p(x)$ is a polynomial function of x. MAPE = 0.22, MAE = 559.7, RMSE = 789.8, R2 = 0.98, μ = 1.18, CoV = 0.25
Guneyisi^21^	Stub (314) Circular [75:25]	*L, D, t, f*_*y*_*, f*_*c*_*′* (*P*_*u*_)	GEP: with ${P}_{u}={P}_{1}{P}_{2}{P}_{3}{P}_{4}{P}_{5}{P}_{6}$ where* ${P}_{1}={\text{sin}}\left[\mathit{sin}\left(\frac{24.4\left(t-D\right)}{{f}_{c}{\prime}}+\left({f}_{c}{\prime}-24.4+{10}^{24}\right)\right)\right]-{f}_{y}$ MAPE% = 7.49, RMSE = 228
Ipek^22^	Stub (103) Double skin [75:25]	*L, D, t, D*_*i*_*, t*_*i*_*, f*_*y*_*, f*_*c*_*′, f*_*yi*_ (*P*_*u*_)	GEP: with ${P}_{u}={P}_{1}{+P}_{2}+{P}_{3}+{P}_{4}+{P}_{5}+{P}_{6}$ where* ${P}_{1}=Dt-D{\text{cos}}\left(t-{D}_{i}-\frac{{f}_{c}{\prime}}{47.4}\right)$ MAPE% = 6.43, RMSE = 85.7, R^2^ = 0.987, a20-index = 0.884, μ = 1.003, CoV = 0.084
Javed^23^	Stub (227) circular [78:22]	*L, D, t, f*_*y*_*, f*_*c*_*′* (*P*_*u*_)	GEP: with ${P}_{u}=D\left(3t-1\right)-{t}^{2}-137.67t-\left(4t+1\right)\frac{L}{D}+\frac{{f}_{y}}{t}+6.72{f}_{c}{\prime}+{\left({f}_{y}-L\right)}^\frac{1}{3}-46.61$ RMSE = 258, R^2^ = 0.98, MAE = 138.7, μ = 1.2, CoV = 0.1
Jiang^24^	Stub (22) Circular	*L, D, t, f*_*y*_*, f*_*c*_*′* (*P*_*u*_)	GEP:${P}_{u}=2D\sqrt{{f}_{c}{\prime}}+{f}_{y}\left(\sqrt{D}-\left(6.219-t\right)\right)+\left[8.078{f}_{y}+0.626L\right]/{\text{tanh}}\left(-2.831\right)$
Naser^25^	Stub + long circular (1245), rectangular (979) [70:30]	*L, D, H, B, t, f*_*y*_*, f*_*c*_*′* (*P*_*u*_)	GA^**+**^**:** ${P}_{u}=0.00439Dt{f}_{y}+0.00072t{D}^{2}+0.00727{f}_{c}{\prime}{D}^{2}+1.38\times {10}^{-5}DL{f}_{c}{\prime}-3.7\times {10}^{-7}DtL{f}_{y}$ μ = 1.02, CoV = 0.13, MAE = 202, RMSE = 295 **GEP**: μ = 1.06, CoV = 0.15, MAE = 238, RMSE = 340
Ren^35^	Stub (180) Square [85:15]	*L, H, t, f*_*y*_*, f*_*c*_*′, E*_*c*_*, E*_*s*_ (*P*_*u*_)	SVM: (Train) R^2^ = 0.932, MAPE% = 14.3, MAE = 239, RMSE = 314 (Test) R^2^ = 0.914, MAPE% = 14.5, MAE = 227, RMSE = 304
Memarzadeh 2023^42^	Stub circular (646) Stub Square (347) [85:15]	*f*_*y*_*, f*_*c*_*′, A*_*c*_*, A*_*s*_*, B/t, *$\lambda$ (*P*_*u*_)	GEP: (Circular) ${P}_{u}={A}_{s}+2{f}_{c}{\prime}-4\lambda +\sqrt{{f}_{c}{\prime}}\left({A}_{c}+\sqrt{3{f}_{c}{\prime}-9.596}\right)+\frac{0.169{A}_{s}\left({f}_{y}-2\lambda \right)\sqrt{{A}_{c}-11.562}}{D/t}$ μ = 0.98, CoV = 0.22, R^2^ = 0.98, a20-index = 72.14, MAE = 242, RMSE = 384 GEP: (Square) ${P}_{u}=3{A}_{c}++9.669{A}_{s}-\frac{B}{t}+{f}_{y}+{A}_{s}{f}_{c}-\frac{\left({f}_{c}{\prime}{A}_{s}^{2}+{f}_{c}{\prime}\right)}{{A}_{c}}-\frac{22.27\lambda \left({f}_{c}{\prime}-\lambda +188.36\right)}{{f}_{y}}$ μ = 0.99, CoV = 0.23, R^2^ = 0.98, a20-index = 0.70, MAE = 324, RMSE = 464 ANN: (Circular) μ = 0.99, CoV = 0.13, R2 = 0.99, a20-index = 0.89, MAE = 134, RMSE = 205 ANN: (Square) μ = 1.01, CoV = 0.12, R^2^ = 0.99, a20-index = 0.916, MAE = 163, RMSE = 254
This study	Circular (674) Rectangular (396) Double skin (246) [80:20]	Table 2	SR: (Circular) μ = 1.01, CoV = 0.075, a20-index = 0.987, MAPE% = 5.856, RMSE = 552kN SR: (Square) μ = 1.013, CoV = 0.072, a20-index = 0.995, MAPE% = 5.856, RMSE = 368.2kN SR: (Double skin) μ = 1.005, CoV = 0.076, a20-index = 0.988, MAPE% = 5.756, RMSE = 194.9kN

* The remaining parameters ${P}_{i}$ have similar expressions to ${P}_{1}$.

+ The expression provided is for circular columns. Similar expressions are introduced for rectangular and circular columns using GA and GEP.

## Research significance

ML models can offer a robust and innovative approach to predicting the axial capacity of CFST columns. Although some existing ML models and simplified design equations have been introduced for CFST column predictions^17,18,26,27^, further work is necessary, primarily for the following reasons:

1. Many researchers directly used axial strength as the output parameter even when its statistical distribution is skewed and biased, without further manipulation or considering its impact on model performance.
2. Existing studies often utilize the entire global database consisting of short and long columns for training/testing ML models. However, the distinct failure mechanisms of long and stub CFST columns can affect the relationships between inputs and strengths. Ipek et al.^28^ conducted a sensitivity analysis to evaluate the performance of the developed ML models using a global database. It was observed that the performance of these models deteriorates for length-to-depth ratios between 2 and 4 while consistently performing well for larger ratios. Additionally, as highlighted by Hou and Zhou^20^, the division of databases into long-column and stub-column subsets significantly enhanced the accuracy of ML methods instead of using the global database. Therefore, this study focuses only on predicting the axial capacity of short columns.
3. While most studies focus on using ANN to predict the axial compression strength of CFST columns, other supervised ML algorithms, such as SVR, GPR, symbolic regression, and tree-based ML algorithms, are less commonly employed.
4. Although the ANN model can introduce design formulas, the resulting formulas include numerous weights, biases, and transfer functions, which are not suitable for engineering practice^16^.
5. As reported in the literature in Table 1, the predicted formulas for designing CFST columns using GA and GEP are efficient and compatible with experimental results. However, a significant drawback is that many of the provided formulas are complicated, unit-dependent, and lack explanations. This paper introduces a novel model to derive simple, practical, unit-independent expressions for predicting the axial compression of CFST columns.

This research collects an extensive experimental database of 1316 specimens from diverse research papers, including circular, rectangular, and double-skin stub CFST columns under axial load without eccentricity. Eight data-driven models are developed, including Gaussian process regression (GPR), symbolic regression (SR), support vector regression optimized with particle swarm optimization (PSVR), artificial neural networks (ANN), XGBoost (XGB), CatBoost (CATB), Random Forest (RF), and LightGBM (LGBM) models. The axial loads reported from the experimental results are normalized to enhance the performance of the ML models. In addition, the proposed formulas are introduced for designing each column type. The hyperparameter tuning of the introduced ML models is performed using the Bayesian Optimization (BO) technique.

## Dataset description

**Figure 1:**
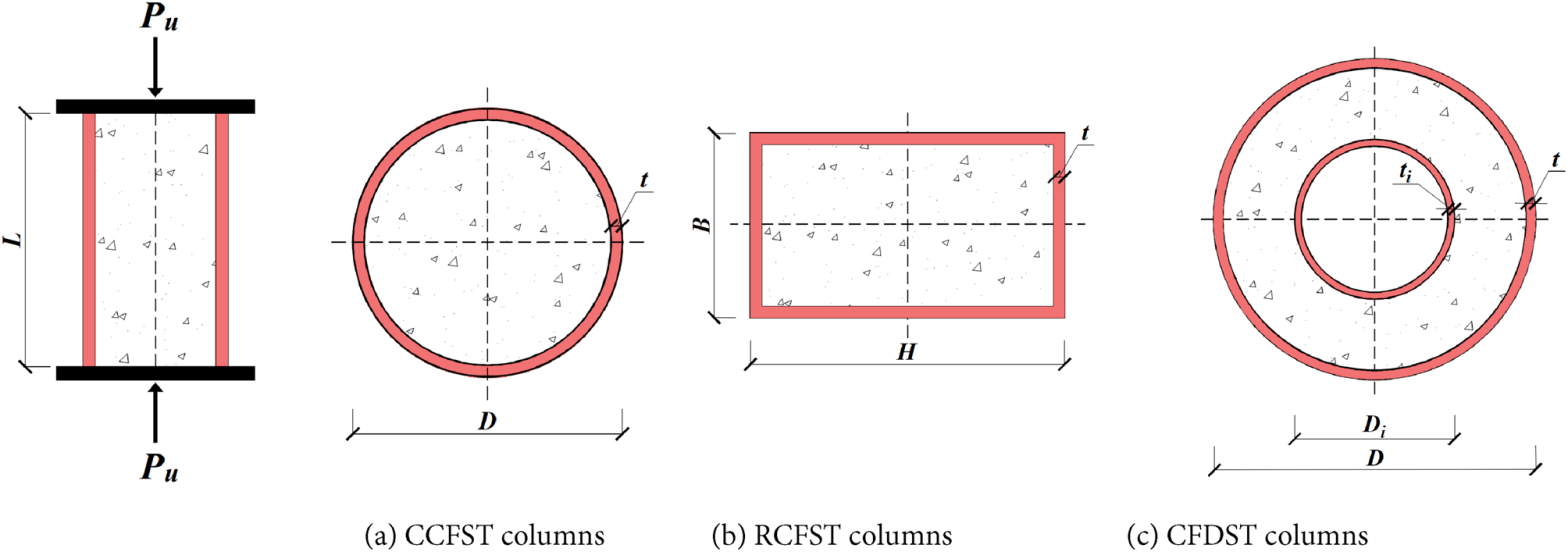
The dimensions of CFST columns.

In this section, a comprehensive experimental database containing 1316 column specimens has been carefully selected from research papers focusing on axially loaded stub CFST columns without eccentricity. The loading and geometric configuration of the specimens are illustrated in Fig. 1. All collected tests were conducted on CFST short columns (with length-to-width ratios smaller than or equal to 4.0^7,8,18^) under monotonic loading and without internal rebar reinforcement. Only samples loaded uniformly across the entire cross-section are considered in the dataset. The database gathered includes the following: (1) Dataset 1 comprises 674 observations with five input parameters related to circular CFST (CCFST) columns; (2) Dataset 2 involves 396 observations with six input parameters relevant to rectangular CFST (RCFST) columns; and (3) Dataset 3 contains 246 observations and involves seven input parameters associated with double-skin CFST (CFDST) columns.

The information presented in Table 2 summarizes the details of the collected specimens, including the outer steel tube diameter (*D* in mm) for circular CFST and CFDST columns, the outside diameter of the inner steel tube (*D*_*i*_ in mm) for CFDST columns, the outer steel tube width (*B* in mm) and outer steel tube depth (*H* in mm) for rectangular CFST columns, the thickness of the outer steel tube (*t* in mm), the thickness of the inner steel tube (*t*_*i*_ in mm), the compressive strength of the core concrete (*f′*_*c*_ in MPa), the yield strength of the outer steel tube (*f*_*y*_ in MPa), the yield strength of the inner steel tube (*f*_*yi*_ in MPa), and the column length (*L* in mm). These parameters are assumed to directly influence the axial capacity (*P*_*u*_) of CFST columns of 1316 observations. Naser et al.^25^ suggested that the remaining material properties of concrete and steel, i.e., Young’s modulus of steel (*E*_*s*_) and concrete (*E*_*c*_) and the ultimate strength of steel (*f*_*u*_), have no significant influence on the training of data-driven models. Table 2 illustrates the statistical distributions of the collected datasets.

**Table 2:** Statistic features of the experimental dataset.

Column cross-section type	Variable	Symbol	Type	Statisticsfoptimi					
				Min	Max	Mean	Std	Skewness	Kurtosis
Circular	Diameter of outer tube	$D$(mm)	Input	60	1100	204.5	163.3	2.71	7.89
	Thickness of outer tube	$t$(mm)	Input	0.52	20	4.95	3.66	2.26	5.99
	Column length	$L$(mm)	Input	152.3	3060	548.9	412.5	2.96	10.25
	Yield strength of outer tube	${f}_{y}$(MPa)	Input	185.7	1153	370.2	141.6	2.46	7.36
	Concrete strength	${f}_{c}{\prime}$(MPa)	Input	15	190	60.6	34.2	1.27	1.31
	Slenderness ratio	$\lambda$	–	0.014	0.438	0.086	0.061	2.2	6.72
	Axial load	${P}_{u}$(MPa)	–	237.4	155,156	6772	18,477	5.05	26.24
	Strength index	${p}_{si}$	Output	0.884	1.686	1.233	0.14	0.54	0.33
Rectangular	Height of outer tube	H (mm)	Input	60	1001	169.8	91.6	3.74	23.42
	Width of outer tube	B (mm)	Input	50.6	1001	161.9	90.2	3.98	26.21
	Thickness of outer tube	$t$(mm)	Input	0.7	20.35	4.92	2.68	1.88	5.96
	Column length	$L$(mm)	Input	60	2499	489.2	260.7	2.77	13.09
	Yield strength of outer tube	${f}_{y}$(MPa)	Input	176.7	1030.6	426.2	192.2	1.25	0.61
	Concrete strength	${f}_{c}{\prime}$(MPa)	Input	11.8	157.54	54.9	26.9	1.2	1.78
	Slenderness ratio	$\lambda$	–	0.437	7.358	1.687	0.993	2.27	8.03
	Axial load	${P}_{u}$(MPa)	–	318	61,980	3127	4395	8.22	93.59
	Normalized load	${p}_{si}$	Output	0.839	1.225	1.036	0.083	0.13	-0.7
Double skin	Diameter of outer tube	$D$(mm)	Input	74.7	356	157.8	55.6	1.37	2.82
	Thickness of outer tube	$t$(mm)	Input	0.59	6.77	3.31	1.61	0.33	-0.98
	Diameter of inner tube	${D}_{i}$(mm)	Input	22	231	72	35.7	1.91	4.9
	Thickness of inner tube	${t}_{i}$(mm)	Input	0.55	10.76	2.92	1.48	1.53	5.58
	Column length	$L$(mm)	Input	117	1500	460.9	220.4	2.29	7.48
	Yield strength of outer tube	${f}_{y}$(MPa)	Input	220	763	357.4	96.2	1.67	3.9
	Yield strength of inner tube	${f}_{yi}$(MPa)	Input	216	1029	393	154.4	2.34	6.15
	Concrete strength	${f}_{c}{\prime}$(MPa)	Input	9.84	141	50.7	26.6	1.55	2.87
	Slenderness ratio	$\lambda$	–	0.039	0.332	0.096	0.051	1.73	3.29
	Axial load	${P}_{u}$(MPa)	–	264	12,015	1895	1737	3.29	14.63
	Normalized load	${p}_{si}$	Output	0.804	1.705	1.122	0.15	0.62	0.73

**Figure 2:**
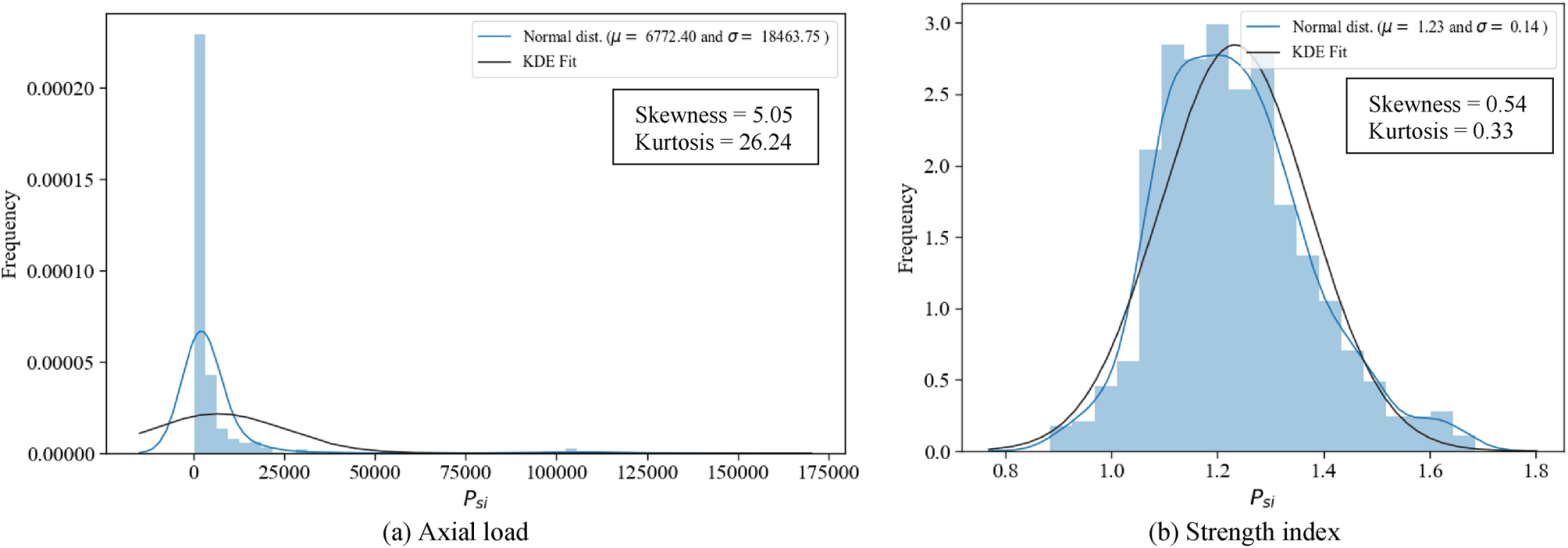
Frequency histogram of the axial load output and strength index for CCFST columns.

Generally, using approximately normally distributed data for machine learning algorithms results in more stable and reliable models. As shown in Fig. 2a, the axial capacity distribution is not normally distributed with extreme skewness for CCFST columns, deteriorating the performance of machine learning models. Therefore, the authors proposed a dimensionless strength index, denoted by *p*_*si*_, as the main output parameter, extracted from normalizing the axial load by dividing the column capacity by the sum of the individual strengths of its components: the steel tubes and core concrete, as defined in Eq. (1).

1 $$p_{si} = \frac{{P_{u} }}{{N_{pl} }}, \quad N_{pl} = A_{s} f_{y} + A_{c} f^{\prime}_{c}$$

where *A*_*s*_ and *A*_*c*_ are the outer steel tube and concrete areas, respectively. Note that for CFDST columns, the contribution of the inner tube, ${A}_{si}{f}_{yi}$ is added to the nominal column capacity, ${N}_{pl}$, in the above equation, where ${A}_{si}$ is the area of the inner steel tube for CFDST columns. The strength index can reflect the confinement efficiency of the CFST column, i.e., a relatively high value of the strength index (*p*_*si*_ > 1.0) indicates high confinement exerted by the outer tube. High confinement exerted by the outer steel tube enhances the actual triaxial strength of inner concrete compared to its uniaxial strength *f*_*c*_*′*. As depicted in Fig. 2b and Table 2, the statistical distribution of the strength index closely resembles a normal distribution. The proximity of strength index values to 1.0 and its physical and dimensionless nature make it easily predictable and interpretable.

The most critical parameter that controls stub column stability is the local slenderness coefficient, *λ*, defined in Eq. (2) for circular and rectangular tubes^29^, as follows:

2 $$\lambda = \frac{D}{t}\left( {\frac{{f_{y} }}{{E_{s} }}} \right) \left( {circular} \right), \quad \lambda = \frac{H}{t}\sqrt {\frac{{f_{y} }}{{E_{s} }}} \left( {{\text{rectangular}}} \right)$$

As shown in Table 2, the database covers a wide range of steel section slenderness, including all compact (λ ≤ 0.15 for circular tubes, λ ≤ 2.26 for rectangular tubes), noncompact (0.15 ≤ λ ≤ 0.19 for circular tubes, 2.26 ≤ λ ≤ 3.0 for rectangular tubes) and slender (λ > 0.19 for circular tubes, λ > 3.0 for rectangular tubes) columns, as recommended by AISC360-22^29^. In addition, the database encompasses a wide range of concrete and steel strengths. As shown in Table 2, the database includes both traditional materials (with *f*_*c*_*′* values below 70 MPa and *f*_*y*_ values below 460 MPa, as suggested in AISC 360-22^29^) and higher strength classes (with *f*_*c*_*′* up to 190 MPa and *f*_*y*_ up to 1153 MPa). It should be noted that most design codes of practice impose limits within their scope of application^29,30^. These restrictions are related to the strengths of steel and concrete materials and the slenderness of steel sections.

**Figure 3:**
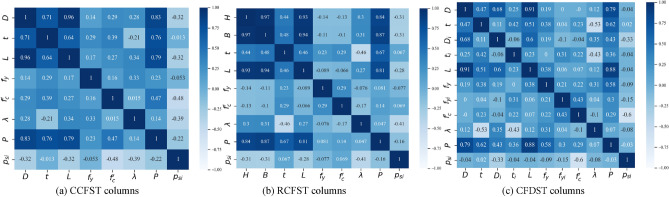
Correlation matrix for the CFST columns database.

Furthermore, Fig. 3 visually presents the correlation matrices of both the input and output variables. As displayed in Fig. 3, the correlation coefficient between any pair of input variables is relatively weak (*ρ* < 0.5), except for the correlations between the outer dimensions of the tube and the column length. In addition, there is a strong relationship between the dimensions of the columns and their axial capacity, which may reduce the performance of the ML training process. However, the correlation between the dimensions and the strength index, *p*_*si*_, is less significant, nearly positive for tube thickness and negative for outer dimensions of the columns and section slenderness, as decreasing the outer dimensions-to-thickness ratio enhances the confinement behavior of stub columns. The yielding strength of the outer steel tube has a negligible impact on the strength index. In contrast, concrete compressive strength is inversely correlated to the strength index for circular and CFDST columns and has a negligible effect on rectangular columns. The high observed correlation for circular sections refers to the ductile behavior of using low-strength concrete. In contrast, the low correlation for rectangular sections refers to the general low confinement provided by the steel tube with a rectangular shape compared to the circular-shaped sections.

## Gaussian process

**Figure 4:**
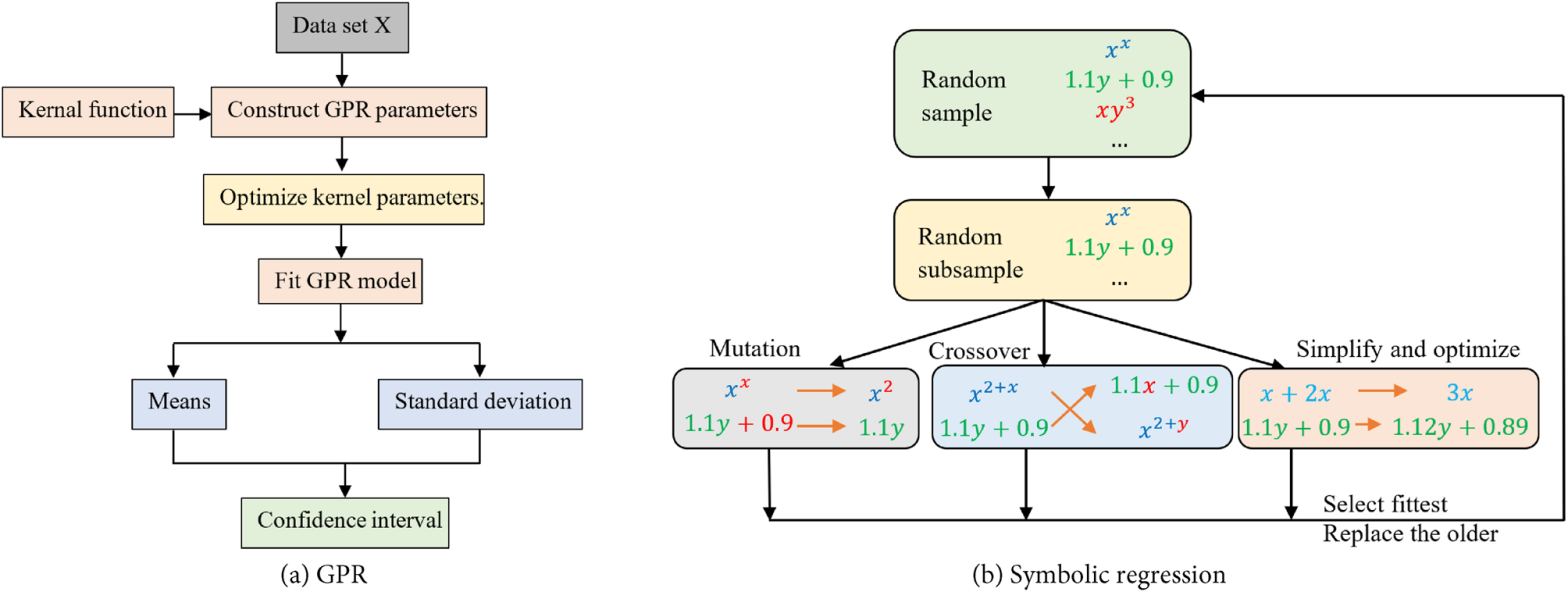
Flow charts of the introduced ML models.

Gaussian processes (GPRs)^10^ are an ML method based on Bayesian learning principles. GPR constructs a Gaussian distribution over functions, as defined in Eq. (3), and observed data points inform this distribution. This technique can effectively handle uncertainty, adapt to noise and complexity levels, and prevent overfitting.

3 $$f\left( x \right)\sim GP\left( {m\left( x \right),K\left( {x,x^{\prime}} \right)} \right)$$

where $f\left(x\right)$ is the function distribution at input $x$, $m\left(x\right)$ is the mean function, and $K\left( {x,x^{\prime}} \right)$ is the covariance (kernel) function determining the covariance between any inputs x and $x^{\prime}$. A combination of kernels, including the Gaussian kernel, Matern kernel, and periodic kernel, are utilized together to capture the different aspects of the data, such as the overall level, smoothness, noise, and variations. The kernel parameters are optimized by maximizing the log-marginal-likelihood^10^. Given observed input‒output pairs, GPR allows predictions for new inputs by inferring a Gaussian distribution over functions as follows:

4 $$p\left(f\left(x\right)|X,y\right)\sim N\left(f\left(x\right)|{\mu }_{p}\left(X\right),{\Sigma }_{p}\left(X\right)\right)$$

where the posterior distribution $p\left(f\left(x\right)|X,y\right)$ is also a Gaussian distribution with a posterior mean function ${\mu }_{p}\left(X\right)$ and a posterior covariance function ${\Sigma }_{p}\left(X\right)$ defined as follows:

5 $${\mu }_{p}\left(x\right)=m\left(x\right)+{K}^{T}\left(X,x\right){\left[K+{\sigma }_{n}^{2}I\right]}^{-1}\left(y-m\left(X\right)\right),$$

6 $${\Sigma }_{p}\left(x\right)=K\left(x,x\right)- {K}^{T}\left(X,x\right){\left[K+{\sigma }_{n}^{2}I\right]}^{-1}K\left(X,x\right)$$

where ${\mu }_{p}\left(x\right)$ and ${\Sigma }_{p}\left(x\right)$ define the mean prediction of the new input point x and the uncertainty (variance) associated with each prediction. The flow chart of the GPR model is illustrated in Fig. 4a.

**Figure 5:**
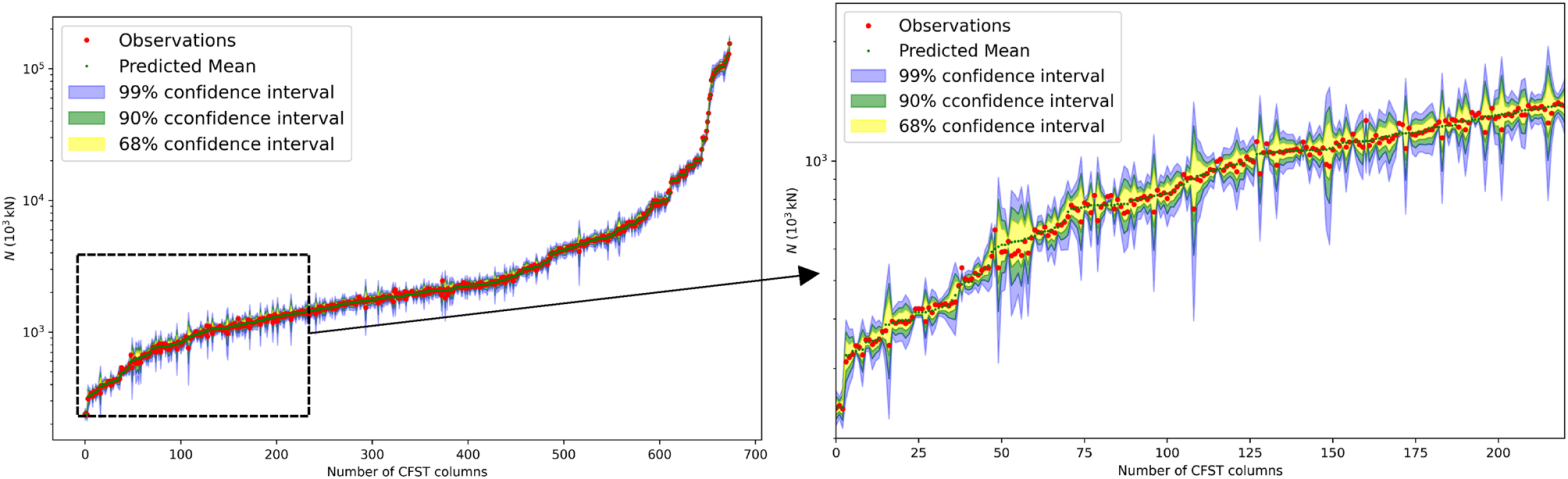
Gaussian process regression on a semilog scale on the y-axis for CCFST columnss.

The GPR model can introduce confidence intervals for prediction outcomes, as illustrated in Fig. 5. This direct quantification of uncertainty enhances its applicability in guiding practical design considerations. The even distribution of the predicted column strength around the measured strength, as depicted in Fig. 5, further substantiates GPR's accurate predictive capabilities for stub CFST column strength.

## Symbolic regression and proposed equations

Symbolic regression (SR)^31,32^ is a supervised learning task and a genetic programming technique^12^ aiming to discover simple and interpretable mathematical expressions that best fit a given dataset by exploring a predefined space of analytic expressions and mathematical functions. SR problems are solved as multi-objective optimization problems, balancing prediction accuracy and model complexity. SR algorithms often use techniques such as genetic programming to improve candidate mathematical expressions by applying the principles of natural selection and evolution to refine the expressions until satisfactory models are found iteratively. In this study, a recent Python library called PySR^33^ is employed to predict mathematical expressions for the axial capacity of stub columns.

**Figure 6:**
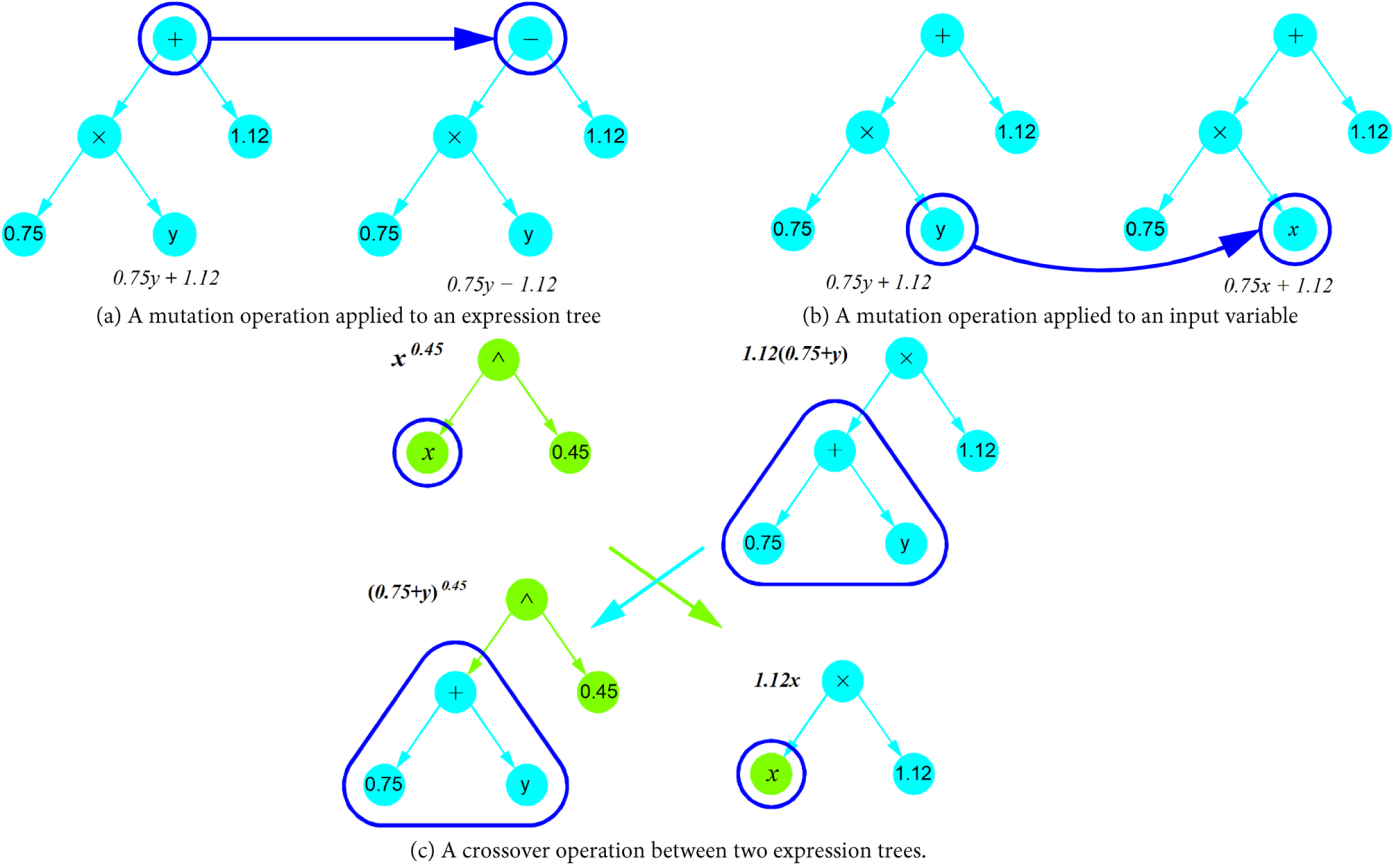
Mutation and crossover operations in SR model.

The SR algorithm starts building an initial population with a random combination of operational symbols or functions (e.g., $+ , - ,/,{*}$, ^ etc.) and terminals, such as input variables and constants, to generate a tree-liked expression for each individual in the population. Individuals are selected in a probabilistic way, giving more possibilities to the best and making it possible for the worst to be selected. Otherwise, if only the best expressions were selected, the algorithm would converge prematurely, making all the populations equal. Consequently, a great part of the search space would be stopped from being explored, and the search would be intensively carried on in a small region only. The selected individuals are mutated or crossed over to produce a new generation of populations, using the fitness function to choose the best individuals in each population generation. The mutation process consists of varying a node at random by replacing a function (Fig. 6a), a terminal (Fig. 6b), or an entire subtree with another random node or subtree, while the crossover operation performs cross-swapping of two subtrees selected randomly in a pair of individuals (Fig. 6c).

In SR modeling, error minimization, and simplicity are key objectives of the fitness function. The fitness function is defined as^33^

7 $$l\left(E\right)={l}_{pred}\left(E\right).{\text{exp}}\left({\text{frecency}}\left[C\left(E\right)\right]\right)$$

where ${l}_{pred}\left(E\right)$ is the prediction loss (chosen as the Mean Absolute Error), $C\left(E\right)$ is the complexity of the expression *E*, defined as the total number of nodes in the expression, and frecency[*C*(*E*)] is a combined measure of frequency and recency of the expression occurring at complexity *C*(*E*) in the population, which is used to avoid excessive growth and redundancies in expressions produced by the SR model. Table 3 specifies the parameters of the SR model used in generating expressions. The main procedures of the SR are introduced in Fig. 4b.

**Table 3:** Statistic features of the experimental dataset.

Parameters	Value	Parameters	Value
Number of generations	100	Allowed Binary operators	+ , *, ^, /, max, min
Total number of populations	60	Loss function	Mean Absolute Error
Population size	60	Constraints	{‘^’:(–1,10)}^(a)^
Maximum length of expressions (total number of nodes)	50	Nested constraints	‘^’:{‘^’:0,’/’:1}, ‘/’:{‘/’:0,’^’:1}^(b)^
Parsimony (factor control the expression complexity)	0.1	denoise	True

(a) The constraint ‘^’:(–1,10) says that power laws can have any complexity in the left argument, but only 10 complexity (nodes) in the right argument.

(b) The nested constraints specify how many times a combination of operators can be nested. The constraint ‘/’:{‘/’:0,‘^’:1} indicates that ‘/’ may never appears within ‘/’, but ‘^’ can be nested once in ‘/’.

The process of selecting the optimal equation requires many iterations and a thorough exploration of each iteration. These iterations involve trying various custom functions, a wide range of operators, and exhaustive combinations of unitless input variables, which have a potential influence on stub column strength, such as the confinement factor ξ, local slenderness ratio λ, global slenderness ratio $\overline{\lambda }$, and cross-section dimension ratios (L/D, L/B, H/B, D_i_/D). Unlike the approach commonly found in the literature^22^, where unit-dependent inputs were used for axial strength prediction using Gene Expression Programming (GEP), the previously mentioned inputs are unitless to enhance the robustness and interpretability of column behavior by avoiding any potential issues related to unit dependencies. The equation derived from each iteration has undergone comprehensive evaluation, simplification and refinement to achieve a concise, understandable, and accurate function. The selection criteria carefully balance various aspects, including equation complexity, accuracy, interpretability, and the sensitivity of its output to variable changes. For circular CFST columns, the following equation is extracted:

8 $$P_{u} = 1.17A_{s} f_{y} + \left[ {0.95 + \frac{1}{{1550\overline{\lambda }^{2} }} + \frac{{0.0027f_{y} }}{{\lambda f^{\prime}_{c} }}} \right]A_{c} f^{\prime}_{c}$$

where $\lambda$ and $\overline{\lambda }$ are the local slenderness and global slenderness ratios, respectively, defined as follows:

$$\lambda = \frac{D}{t}\left( {\frac{{f_{y} }}{{E_{s} }}} \right), \overline{\lambda } = \sqrt {\frac{{N_{pl} }}{{N_{cr} }}} \le 0.5, N_{pl} = f_{y} A_{s} + 0.85f^{\prime}_{c} A_{c} ,N_{cr} = \frac{{\pi^{2} \left( {EI_{eff} } \right)}}{{L^{2} }},EI_{eff} = E_{s} I_{s} + 0.6E_{c} I_{c} ,$$

where ${E}_{s}{I}_{s}$ and ${E}_{c}{I}_{c}$ are the flexural stiffness of steel and concrete parts.

Regarding the rectangular CFST columns, the proposed equation is:

9 $${P}_{u}={A}_{s}{f}_{y}+\left[0.85+\frac{0.31}{{\text{max}}\left({\lambda ,}0.83{\beta }^{{\text{max}}\left(1,\xi \right)}\right)}\right]{A}_{c}{f}_{c}^{\mathrm{^{\prime}}}$$

Here, $$\xi =\frac{{A}_{s}{f}_{y}}{{A}_{c}{f}_{c}^{\mathrm{^{\prime}}}}, \beta =\frac{H}{B} ,$$ and $$\lambda =\frac{B}{t}\sqrt{\frac{{f}_{y}}{{E}_{s}}}$$

For double-skin CFST circular columns, the equation is:

10 $$P_{u} = A_{s} f_{y} + A_{si} f_{yi} + \left( {\alpha^{2} + \frac{{E_{s} }}{{5000f^{\prime}_{c} \alpha }}\left[ {\xi + \frac{{f^{\prime}_{c} }}{{f_{y} }}} \right]} \right)^{0.3} A_{c} f^{\prime}_{c}$$

Here, $$\xi =\frac{{A}_{s}{f}_{y}}{{A}_{c}{f}_{c}^{\mathrm{^{\prime}}}}, \alpha =\frac{{D}_{i}}{D},$$ and $$0.2\le \alpha \le 0.85$$.

The proposed equations establish a comprehensive and simple framework with meaningful physical interpretations for predicting the axial capacity of various CFST columns. In the context of the circular column formula in Eq. (8), it is evident that increasing the square of global slenderness $\overline{\lambda }$, local slenderness *λ* or the *f*_*c*_*′/f*_*y*_ ratio reduces axial capacity. Concerning the rectangular section equation in Eq. (9), the axial strength of the composite column decreases with increasing local slenderness *λ* or H/B ratio; for the double-skin CFST columns formula in Eq. (10), an increase in the confinement ratio reduces the column capacity. These observations align with the experimental behavior of CFST columns. In addition, the provided equations are simple and unit-independent and have physical meaning compared to the previous studies in Table 1.

## Data preprocessing and hyperparameter optimization technique

The min–max scaling technique is employed for data normalization to reduce the negative impact of multidimensionality. The grid searching technique is utilized for tuning the models’ hyperparameters during the training phase, and fivefold cross-validation is employed to mitigate the overfitting issues. After normalization, datasets were divided into two distinct training and testing subsets. The objective of segregating testing subsets was to assess how well the trained models perform on the new unseen datasets. As widely reported by many studies^13,14,34^, eighty percent of the original dataset is allocated randomly for training, leaving the remaining 20% for testing. To compare and evaluate the effectiveness and reliability of the introduced models, six different ML models, including the support vector machine integrated with particle swarm optimized (PSVR)^35^, Artificial Neural Network (ANN), XGBoost (XGB), CatBoost (CATB), Random Forest (RF), and LightGBM (LGBM) models, were introduced. All the introduced ML models were constructed and evaluated using the same training and testing subsets for a fair comparison.

The performance of most ML algorithms largely depends on their hyperparameters, which are predefined before model training. Properly tuning these hyperparameters is necessary to guarantee the optimum prediction performance. Searching for the optimum hyperparameters involves trying out different values for each and selecting the combination that introduces the best performance on the validation data. Using traditional techniques, i.e., grid search (GS) and random search (RS), is time-consuming, especially for large search spaces with numerous hyperparameters. In contrast, Bayesian Optimization (BO) models using the surrogate function, i.e., Gaussian process, random forest, and tree-structured Parzen estimators models (TPE)^36^, guide the selection of the next hyperparameter value based on the previous results from tested hyperparameter values. This approach minimizes unnecessary evaluations, enabling BO to identify the optimal hyperparameter combination in fewer iterations than the GS and RS methods^37^. In this study, we adopted the TPE model^36^ to optimize the introduced ML models due to their robustness compared to other surrogate functions^37^. Mean Absolute Percentage Error, MAPE is chosen as the objective function in the validation dataset. The expected improvement (EI) of TPE, defined in Eq. (11), builds a probability model of the objective function and uses it to select the most promising hyperparameters to evaluate in the true objective function^36^:

11 $$EI_{{s^{*} }} \left( z \right) = \frac{{{\text{constant}}\;{\text{w}}.{\text{r}}.{\text{t }}\left( z \right)}}{{\gamma + \left( {1 - \gamma } \right)\frac{g\left( z \right)}{{l\left( z \right)}}}}$$

where z is the hyperparameter combination chosen from the search space, and s^*^ is a threshold chosen to be some quantile γ of the observed s values, so that $p\left(s<{s}^{*}\right)=\gamma$. Additionally, $l\left(z\right)$ and $g\left(z\right)$ correspond to two distinct distributions: one where the objective function values are below the threshold, l(z), and another where the values exceed the threshold, g(z). To maximize EI, TPE focuses on drawing samples of hyperparameters with the maximum l(z)/g(z) ratios, from Eq. (11). Finally, cross-validation was applied to assess the introduced models' effectiveness, avoid overfitting, and obtain accurate predictions for the testing data.

## Performance and results of ML models

**Figure 7:**
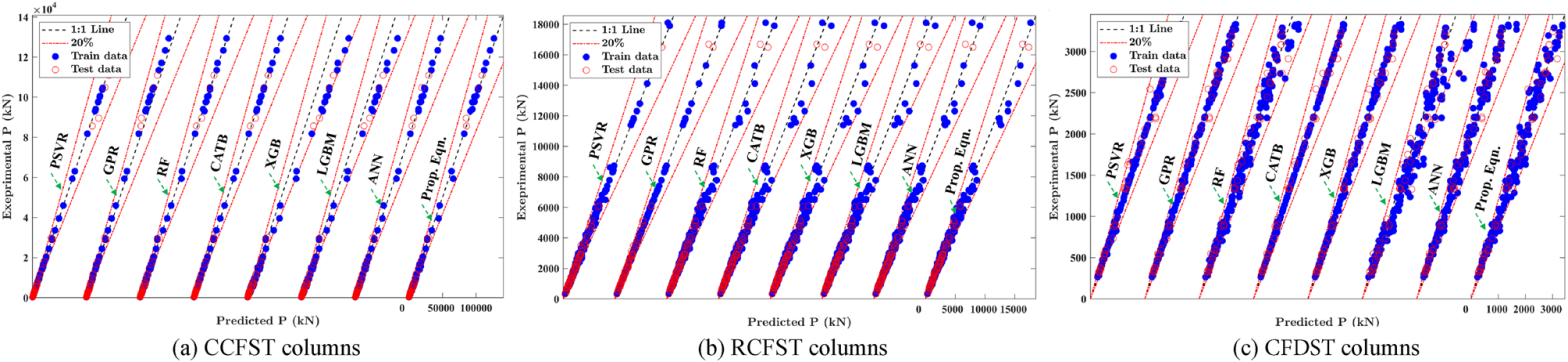
Comparison between proposed equations and ML models for training and testing datasets.

The scatter plots in Fig. 7 illustrate the relationship between experimental and predicted outcomes for various ML models applied to training and testing datasets for columns with different cross-section shapes. It can be observed that the data points tightly gather around the diagonal line for most of ML models, signifying a strong alignment between the model predictions and experimental results. This alignment signifies the reliability and prediction accuracy of the developed models. Table 4 introduces evolution metrics to assess the performance of the established ML models, including the mean (*μ*), coefficient of variance (CoV), coefficient of determination (*R*^*2*^), root mean squared error (RMSE), the mean absolute percentage error (MAPE), and a20-index, defined as follows:

12 $$\begin{aligned} & \mu = \frac{1}{n}\mathop \sum \limits_{i = 1}^{n} \frac{{y_{i} }}{{\hat{y}_{i} }}, \;\;R^{2} = 1 - \frac{{\mathop \sum \nolimits_{i = 1}^{n} \left( {\hat{y}_{i} - y_{i} } \right)^{2} }}{{\mathop \sum \nolimits_{i = 1}^{n} \left( {y_{i} - \overline{y}} \right)^{2} }} RMSE = \sqrt {\frac{1}{n}\mathop \sum \limits_{i = 1}^{n} \left( {\hat{y}_{i} - y_{i} } \right)^{2} } , \\ & MAPE = \frac{1}{n}\mathop \sum \limits_{i = 1}^{n} \left| {\frac{{\hat{y}_{i} }}{{y_{i} }} - 1} \right| \times 100\% \\ \end{aligned}$$

where ${y}_{i}$ and ${\widehat{y}}_{i}$ are the actual and predicted output values of the *i-*th sample, respectively, $\overline{y }$ is the mean value of experimental observations, and *n* is the number of specimens in the database. The a20-index^16,38^ measures the percentage of samples with actual to prediction ratio, ${\widehat{y}}_{i}/{y}_{i}$, falling within the range of 0.80–1.20. All data generated and algorithms introduced in this study are included in the supplementary file.

**Table 4:** Comparison of the developed ML models for different column types.

Metrics	Training data				Testing data				All data				EC4^30^	AISC^29^
Circular Column	Prop. Eqn	CATB	GPR	PSVR	Prop. Eqn	CATB	GPR	PSVR	Prop. Eqn	CATB	GPR	PSVR		
Mean $\mu$	1.012	1	0.999	1.001	1.006	0.998	0.996	0.996	1.011	1	0.998	1	1.041	1.294
CoV	0.076	0.017	0.026	0.038	0.074	0.054	0.065	0.064	0.075	0.029	0.037	0.045	0.091	0.253
R^2^	0.999	1	1	1	0.999	1	0.999	0.997	0.999	1	1	0.999	0.999	0.975
MAPE%	5.881	0.711	1.792	1.945	5.758	4.124	5.034	4.699	5.856	1.394	2.442	2.497	7.175	29.45
RMSE(kN)	568	89.9	125.3	108.5	482.8	297	359.4	912.8	552	155.3	196.1	419.9	424.1	2916.2
a20-index	0.987	1	0.998	0.996	0.985	1	1	0.985	0.987	1	0.999	0.994	0.957	0.286

Rectangular Column	Prop. Eqn	GPR	PSVR	RF	Prop. Eqn	GPR	PSVR	RF	Prop. Eqn	GPR	PSVR	RF	EC4^30^	AISC^29^
Mean $\mu$	1.012	0.999	1	0.999	1.02	1.012	1.007	1.002	1.013	1.002	1.002	1	1.036	1.15
CoV	0.074	0.014	0.054	0.064	0.065	0.046	0.059	0.059	0.072	0.024	0.055	0.063	0.087	0.168
R^2^	0.993	1	0.999	0.997	0.996	0.997	0.997	0.996	0.993	1.000	0.999	0.997	0.994	0.983
MAPE%	6.03	1.007	3.675	5.216	5.156	3.573	4.459	4.89	5.856	1.518	3.832	5.151	7.26	15.437
RMSE(kN)	402.8	34.2	140	261.1	168.9	150.5	136	175.5	368.2	73.9	139.2	246.5	333.8	571.4
a20-index	0.997	1	1	1	0.987	1	1	1	0.995	1	1	1	0.99	0.694

Double-Skin Column	Prop. Eqn	XGB	GPR	CATB	Prop. Eqn	XGB	GPR	CATB	Prop. Eqn	XGB	GPR	CATB	EC4^30^	AISC^29^
Mean $\mu$	1.007	0.999	0.999	1	1.031	1.008	1.009	1.014	1.005	1.001	1.001	1.003	1.032	1.252
CoV	0.075	0.025	0.025	0.014	0.085	0.044	0.047	0.048	0.076	0.03	0.031	0.025	0.102	0.236
R^2^	0.986	0.998	0.998	0.998	0.985	0.994	0.995	0.995	0.987	0.997	0.998	0.998	0.98	0.893
MAPE%	5.887	1.797	1.601	0.394	6.469	3.494	3.556	3.573	5.756	2.135	1.99	1.028	8.538	25.43
RMSE(kN)	217.2	91.6	87.6	81.4	119.9	77.6	71.3	70.8	194.9	89	84.6	79.4	246.4	568.1
a20-index	0.995	1	1	1	0.939	1	1	1	0.988	1	1	1	0.951	0.411

As shown in Table 4, all introduced ML models display mean *μ*, *R*^*2*^, and a20-index values close to 1.0 and small values for CoV, MAPE%, and RMSE for different cross sections. The prediction results of all introduced models exhibit CoV less than 0.076, MAPE% lower than 6%, and RMSE less than 552 kN, indicating minimized scattering in the prediction results compared to the experimental results. Table 4 reveals that the CATB, GPR, and XGB models introduce the best evaluation metrics for the testing subsets, with MAPE% values equal to 1.394%, 1.518%, and 2.135% for CCFST, RCFST, and CFDST column datasets, respectively. In addition, PSVR can accurately predict the capacity of stub CFST columns with MAPE% values equal to 2.497 and 5.151 for CCFST and RCFST columns, respectively. The superior predictive capability of PSVR demonstrates that the SVR model, incorporating the metaheuristic optimization methods^39^ like the PSO algorithm, can significantly enhance the performance of the SVR model.

Furthermore, the evolution metrics of the testing resemble those of the training set, except for the GPR and CATB models. However, the performance of GPR and CATB models in the testing set is comparable to that of the remaining data-driven models and even better than that of other ML models. In addition, when examining the *R*^*2*^ value and a20-index for the entire dataset, it was found that they are nearly identical to those of the test and training subdatasets. Such robust and stable alignment between the performance of sub-datasets signifies a minimal occurrence of overfitting during the training process of the models.

Although the GPR, CATB, XGB models stands out with significantly superior results compared to other models, extracting an explicit design formula from these models is a challenging task. In contrast, the proposed equations extracted from the SR algorithm offer a distinct advantage by providing simple and practical explicit design formulas, making them more accessible and easier to interpret, even with slightly lower accuracy than the introduced ML models. Although ANN could provide accurate and explicit formulas for strength prediction, utilizing the network in engineering design might not be practical due to the lengthy formulas of the ANN model.

The compressive strength predictions of CFST columns by the proposed equations were compared with the existing code formulas, including EC4^30^ and AISC360^29^ for different types of columns. As observed in Table 4, for all types of CFST columns, the proposed equations attain a mean, R2, and a20-index nearly equal to 1.0 with CoV less than 0.076 and MAPE% less than 5.9, while EC4 and AISC360 show CoV larger than 0.091, 0.168 with MAPE% larger than 7.1% and 15%, respectively. In addition, the AISC360 predictions, compared to EC4 predictions, appear to overestimate the axial capacity for different cross sections with a higher mean approaching 1.20, lower a20-index, and relatively high error indices. The RMSE and MAPE of AISC360^29^ predictions are approximately two to six times those of EC4^30^, indicating the better performance of EC4 compared to AISC360. In addition, AISC360 introduces an a20-index with a value approximately 50% lower than that obtained from the EC4 results. This discrepancy could stem from the absence of confinement effect calculations in AISC360^29^. Although all cited codes’ standards display a safe design, the error indices introduced by the ML models and proposed equation are significantly small compared to these standards. Specifically, the proposed equations demonstrate superior performance compared to these standards across all evaluation criteria.

**Figure 8:**
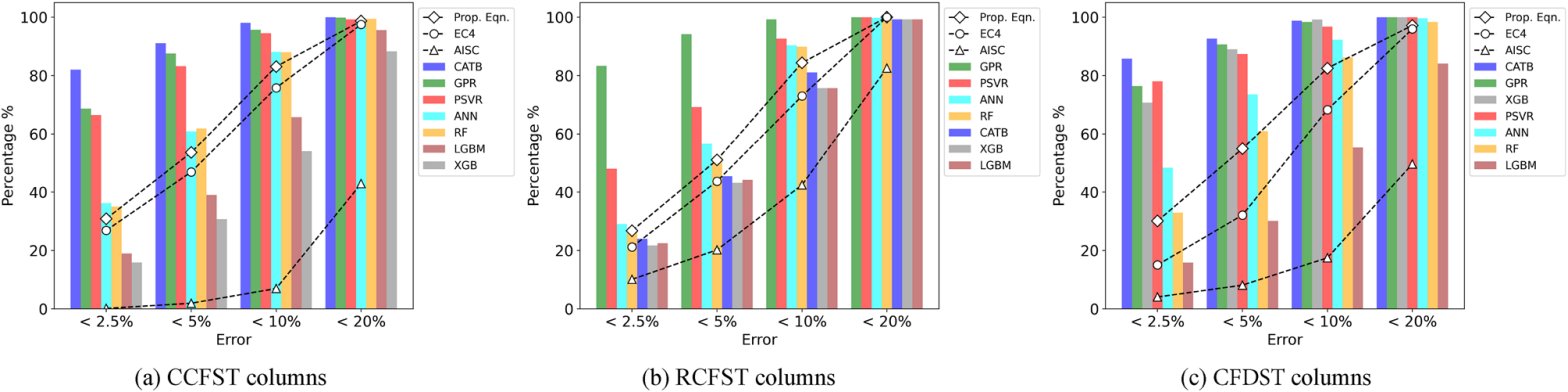
Prediction errors of design standards and established ML models.

Figure 8 displays the prediction errors of the design standards and the developed ML models for different cross sections. It indicates that most of the introduced ML models are more accurate than the design standards, especially for the GPR, CATB, and PSVR models, implying the superiority of these ML methods in estimating the axial capacity of stub CFST columns. In the case of CCFST columns, the CATB, GPR, and PSVR models display more than 95% of test samples within the 10% error range, while the proposed equation, EC4, and AISC360 show 83%, 75%, and 7% of test samples, respectively, within the same range. For RCFST columns, all ML models exhibit accuracy, with over 75% of test samples falling within a 10% error range, while the corresponding proportions for the proposed equation, EC4, and AISC360 are 85%, 73%, and 42%, respectively. Regarding CFDST columns, all ML models, excluding the RF and LGBM models, correctly predict 90% of the specimens within a 10% range error, while the proposed equation, EC4, and AISC360 attain nearly 83%, 68%, and 17% accuracy for the test samples, respectively, within the same error range. Thus, the introduced ML models can be considered valuable tools alongside the design standards in estimating the axial capacity of stub CFST columns.

## Feature importance analysis

**Figure 9:**
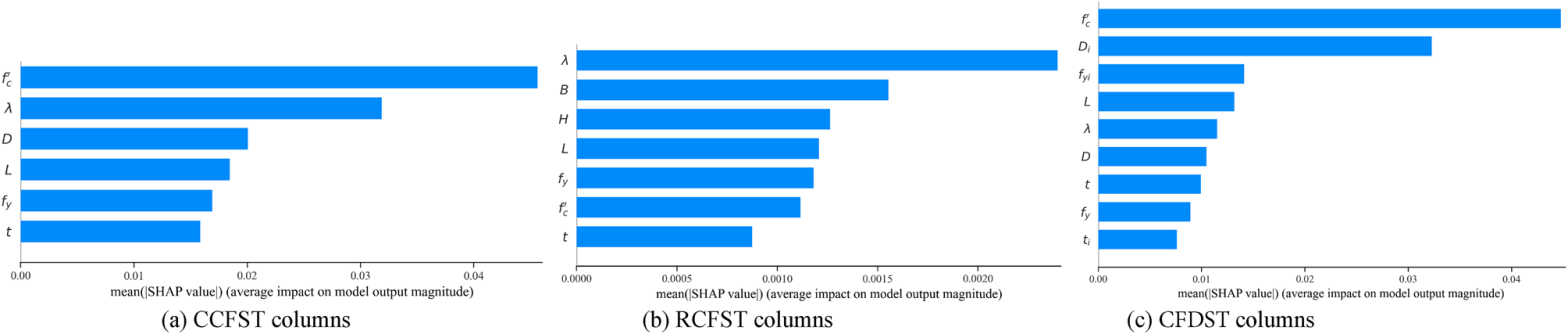
Summary plot for stub CFST column database.

**Figure 10:**
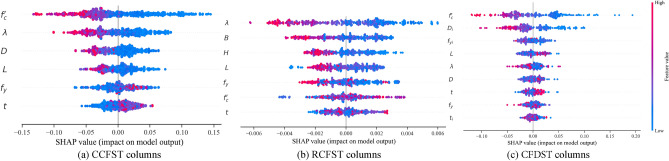
SHAP feature importance for stub CFST column database.

**Figure 11:**
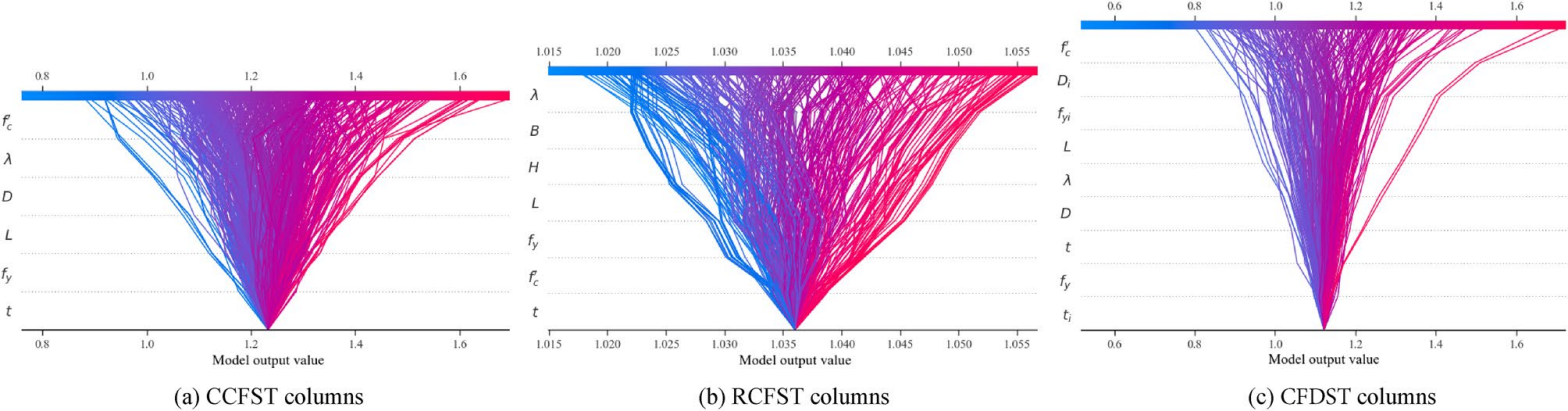
SHAP decision plots for stub CFST column database.

Evaluating the influence of input parameters on axial compressive strength is a critical aspect of designing CFST columns. This study employs the Shapley Additive Explanation (SHAP) method to analyze the impact of input parameters on the strength index^40^. As illustrated in Fig. 9, the summary plot provides the impact of each feature on a model's predictions and defines the relative importance of each feature on the axial strength. Figure 10 displays the SHAP feature importance for each input feature. A feature importance value greater than zero indicates a positive correlation between the variable and the strength index, while a value less than zero signifies a negative impact on the strength index. The SHAP decision plots in Fig. 11 reveal the complex decision-making process of ML models and to observe how the summary plot works globally in predicting axial compressive strength for CFST columns. The compressive concrete strength and the slenderness ratio stand out as the most influential design parameters within the dataset, especially for CCFST and CFDST columns. The remaining variables' feature importance is ranked in descending order. Additionally, it is observed that, except for yield strength *f*_*y*_ and steel tube thickness, *t*, all other input variables negatively influence or have a mixed impact on the strength index. This suggests that an increase in the outer tube yield strength and thickness will enhance the performance of stub columns, while increasing the section slenderness ratio and concrete strength will negatively impact the compressive strength index.

## Conclusion

In conclusion, this study compiled a comprehensive experimental database of 1316 datasets from various research papers, including circular, rectangular, and double-skin CFST short columns under axial loading without eccentricity. The datasets were carefully selected to ensure reliable and consistent results. Normalization of the axial load was performed to enhance the performance of the data-driven models using a unitless variable termed the strength index. Various data-driven models, including Gaussian process regression (GPR), symbolic regression (SR), support vector regression optimized with particle swarm optimization (PSVR), and artificial neural networks (ANN), XGBoost (XGB), CatBoost (CATB), Random Forest (RF), and LightGBM (LGBM) models, were developed and evaluated. In addition, the proposed formulas are presented for designing circular, rectangular, double-skin CFST columns. The following conclusions can be drawn:

- The proposed normalization approach of the axial load yields a nearly normal distribution, which improves model performance and robustness. In addition, using the strength index as an output parameter reflects the insights into the level of confinement provided by the outer tube for different column types.
- The CATB, GPR, and XGB models stood out as the most accurate and reliable models for strength predictions of CCFST, RCFST, and CFDST column datasets, respectively, while the proposed equations offered simple and practical expressions with acceptable accuracy.
- Symbolic regression emerges as a promising methodology for extracting empirical equations endowed with practical applicability and meaningful physical interpretations.
- The proposed equations demonstrated their reliability and robustness compared to existing design code standards.
- SHAP analysis revealed that an increase in the outer tube yield strength and thickness will enhance the performance of stub columns, while increasing the section slenderness ratio and concrete strength will negatively impact the compressive strength index.

In summary, the proposed data-driven models can extract the axial compression capacity of CFST stub columns with reliable and accurate results, making them valuable tools for structural engineers.

### Supplementary Information

Supplementary Information.

## Data Availability

All data generated or analyzed during this study are included in this published article and available in a public repository https://github.com/kmegahed/Stub-CFST-Machine-learning.
