# Supplementary material for: Prediction of the axial compression capacity of stub CFST columns using machine learning techniques
Source: Sci Rep. 2024 Feb 5;14:2885. doi: 10.1038/s41598-024-53352-1 (PMC10838919; doi:10.1038/s41598-024-53352-1)
Supplement: Supplementary file 1 — Supplementary Information. [file 41598_2024_53352_MOESM1_ESM.zip › supplementary data/data summary.docx]

Table 1: Summary of the test data of the circular CFST column.

| $\boldsymbol{n}$ | $\boldsymbol{D}$ (mm) | $\boldsymbol{t}$ (mm) | $\boldsymbol{f}_{\boldsymbol{y}}$ (MPa) | $\boldsymbol{f}_{\boldsymbol{c}}^{\boldsymbol{'}}$ (MPa) | Ref. | $\boldsymbol{n}$ | $\boldsymbol{D}$ (mm) | $\boldsymbol{t}$ (mm) | $\boldsymbol{f}_{\boldsymbol{y}}$ (MPa) | $\boldsymbol{f}_{\boldsymbol{c}}^{\boldsymbol{'}}$ (MPa) | Ref. |
| --- | --- | --- | --- | --- | --- | --- | --- | --- | --- | --- | --- |
| 14 | 76-153 | 1.7-4.9 | 363-633 | 21-43 | [1] | **13** | 114-115 | 3.8-5 | 343-365 | 25-95 | [2] |
| 11 | 168-169 | 2.6-5 | 221-317 | 18-37 | [3] | **12** | 100-200 | 3 | 304 | 50 | [4] |
| 1 | 83 | 1.4 | 483 | 41 | [5] | **26** | 108-450 | 3-6.5 | 279-853 | 25-85 | [6] |
| 12 | 100-160 | 1.5-4.5 | 232-410 | 28-40 | [7] | **4** | 120-180 | 1.5 | 223 | 37-41 | [8] |
| 10 | 92-210 | 1.5-4 | 232-434 | 20-47 | [9] | **6** | 165-219 | 2.7-4.8 | 350 | 35-68 | [10] |
| 30 | 101-160 | 0.6-5.3 | 265-357 | 23-39 | [11] | **4** | 100 | 1.9 | 404 | 112 | [12] |
| 1 | 109 | 4.6 | 272 | 30 | [13] | **3** | 112-114 | 1.9-3.6 | 260-261 | 40-48 | [14] |
| 16 | 166-320 | 5-7 | 250-275 | 27-47 | [15] | **7** | 76 | 2.5-3.3 | 278-305 | 145 | [16] |
| 3 | 108 | 4 | 339 | 29 | [17] | **6** | 114 | 2.7-5.9 | 235-355 | 56-107 | [18] |
| 10 | 100-102 | 0.5-5.7 | 244-320 | 18-37 | [19] | **2** | 165 | 2.4 | 288 | 24 | [20] |
| 2 | 150 | 0.7 | 245 | 23-33 | [21] | **3** | 558-559 | 16.5 | 546 | 25 | [22] |
| 10 | 159-1020 | 5.1-13.3 | 291-392 | 15-46 | [23] | **36** | 153-477 | 1.5-11.4 | 290-345 | 76 | [24] |
| 12 | 174-179 | 3-9 | 249-283 | 22-46 | [25] | **11** | 114-219 | 3.6-10 | 300-428 | 50-178 | [26] |
| 1 | 324 | 5.6 | 444 | 92 | [27] | **7** | 100-168 | 2.5-3 | 318-446 | 34-95 | [28] |
| 2 | 152 | 1.7 | 270 | 73 | [29] | **20** | 140 | 4.4-16.7 | 359-1153 | 53-125 | [30] |
| 4 | 111-133 | 2-4.5 | 324-355 | 67 | [31] | **9** | 108-115 | 2.1-8 | 252-304 | 59-131 | [32] |
| 6 | 190 | 1.1 | 203 | 95-110 | [33] | **40** | 89-169 | 2.1-12.1 | 336-460 | 40-79 | [34] |
| 13 | 108-133 | 1-4.7 | 232-358 | 77-85 | [35] | **36** | 153-477 | 1.5-11.4 | 290-345 | 76 | [24] |
| 15 | 165-190 | 0.9-2.8 | 186-363 | 41-108 | [36] | **12** | 297-302 | 4.5-11.9 | 348-472 | 27-79 | [37] |
| 13 | 101-319 | 3-10.4 | 331-452 | 23-53 | [38] | **36** | 129-133 | 3-5 | 306 | 46-67 | [39] |
| 2 | 120 | 2.7 | 340 | 15-29 | [40] | **26** | 60-250 | 1.9-2 | 282-404 | 76-80 | [41] |
| 2 | 158 | 1.5-2.1 | 286-308 | 19 | [42] | **4** | 76 | 2-3 | 225 | 30 | [43] |
| 4 | 114 | 6-8 | 368-370 | 79-82 | [44] | **3** | 280 | 8 | 495 | 38-117 | [45] |
| 6 | 76-100 | 2-3 | 255 | 30 | [46] | **2** | 140-180 | 3 | 295-321 | 37 | [47] |
| 2 | 180 | 4 | 340 | 45 | [48] | **8** | 275-1100 | 4.14-16.48 | 260-281 | 46 | [49] |
| 4 | 240 | 3.05 | 571-649 | 49.82 | [50] | **1** | 327 | 2 | 289 | 35 | [51] |
| 11 | 219-376 | 3.28-7.06 | 305-429 | 45-73 | [52] | **1** | 112 | 3 | 392 | 19 | [53] |
| 15 | 141-262 | 2.11-3.04 | 691-734 | 39 | [54] | **3** | 89-169 | 3.98-7.96 | 330-411 | 23 | [55] |
| 1 | 140 | 4 | 310 | 27 | [56] | **3** | 133 | 4.75 | 338 | 25 | [57] |
| 6 | 108 | 6-10 | 344-385 | 101-117 | [58] | **2** | 60.3 | 4.5 | 225 | 47 | [59] |
| 1 | 159 | 4 | 467 | 25 | [60] | **21** | 600-1000 | 8-20 | 435-735 | 110-190 | [61] |
| 4 | 114 | 6-8 | 400-406 | 66-69 | [62] | **4** | 76 | 2-3 | 255 | 30 | [63] |
| 6 | 165 | 4.25 | 351 | 34-49 | [64] | **2** | 189 | 5.09-5.11 | 347-464 | 38 | [65] |
| 2 | 102 | 3.4 | 311 | 72 | [66] | **2** | 159 | 4.64 | 397 | 47 | [67] |
| 6 | 114-133 | 4.5-6 | 335-382 | 42-64 | [68] | **2** | 90 | 3-4.5 | 357-381 | 36 | [69] |
| 2 | 114 | 2.5 | 365 | 47-66 | [68] | **4** | 159 | 4.5-6 | 318-325 | 31 | [70] |
| 1 | 203 | 4 | 300 | 36.7 | [68] | **2** | 140-180 | 3 | 295-321 | 31-37 | [71] |
| 4 | 140-180 | 3-6 | 300-320 | 37 | [72] | **10** | 165 | 2.3-4.5 | 332-386 | 34 | [73] |
| 2 | 150 | 3.55 | 326 | 25-31 | [74] | **3** | 114 | 4 | 330 | 29-43 | [75] |
| 2 | 114 | 3.01-4.03 | 301-331 | 48 | [76] | **2** | 250-300 | 4 | 276 | 26 | [77] |
| 4 | 87 | 1.9 | 255 | 26 | [78] | **3** | 165 | 2-4 | 242 | 29 | [79] |
|  |  |  |  |  |  | **674** | **60-1020** | **0.5-16.7** | **186-1153** | **15-178** | **Total** |

Table 2: Summary of the test data of the rectangular CFST column.

| $\boldsymbol{n}$ | $\boldsymbol{B}$ (mm) | $\boldsymbol{H}$ (mm) | $\boldsymbol{t}$ (mm) | $\boldsymbol{f}_{\boldsymbol{y}}$ (MPa) | $\boldsymbol{f}_{\boldsymbol{c}}^{\boldsymbol{'}}$ (MPa) | Ref. | $\boldsymbol{n}$ | $\boldsymbol{B}$ (mm) | $\boldsymbol{H}$ (mm) | $\boldsymbol{t}$ (mm) | $\boldsymbol{f}_{\boldsymbol{y}}$ (MPa) | $\boldsymbol{f}_{\boldsymbol{c}}^{\boldsymbol{'}}$ (MPa) | Ref. |
| --- | --- | --- | --- | --- | --- | --- | --- | --- | --- | --- | --- | --- | --- |
| 7 | 100 | 100 | 2.2-4.3 | 194-339 | 20-32 | [80] | **3** | 250-251 | 250-251 | 3.7-3.8 | 324 | 33 | [81] |
| 4 | 150-200 | 150 | 0.7-1.4 | 245 | 23-34 | [21] | **6** | 150 | 150 | 4-6 | 416-437 | 88 | [82] |
| 13 | 150 | 100 | 5 | 363 | 41 | [83] | **4** | 200-300 | 200-300 | 3-8 | 414-464 | 47 | [84] |
| 17 | 120-250 | 120-250 | 5-8 | 300-439 | 31-103 | [85] | **2** | 200 | 200 | 2-3.5 | 301-315 | 50 | [86] |
| 4 | 305 | 305 | 5.8-8.9 | 269-660 | 110 | [87] | **8** | 110-180 | 110-180 | 5 | 701 | 21-55 | [88] |
| 7 | 100-301 | 100-301 | 2.2-6.1 | 300-395 | 27-64 | [38] | **3** | 150-203 | 102-150 | 8.3 | 488 | 36-47 | [89] |
| 20 | 120-200 | 120-200 | 3.8-5.9 | 321-330 | 12-46 | [90] | **2** | 300 | 300 | 3.7-3.8 | 311 | 32-49 | [91] |
| 30 | 101-173 | 101-142 | 2-5.1 | 255-347 | 47-67 | [92] | **7** | 74-150 | 74-150 | 4.9 | 762 | 100 | [93] |
| 18 | 90-160 | 70-135 | 2.9-7.6 | 194-228 | 51 | [94] | **9** | 150 | 150 | 8-12.5 | 446-779 | 141-158 | [26] |
| 6 | 100-183 | 98-181 | 4.2 | 550 | 62-73 | [95] | **6** | 197-201 | 197-201 | 6.1-10.3 | 382-438 | 19-21 | [96] |
| 38 | 119-323 | 119-323 | 4.4-9.5 | 262-835 | 25-91 | [6] | **9** | 200-402 | 200-203 | 3.7-3.8 | 311 | 29-49 | [97] |
| 6 | 125 | 125 | 3.2-6 | 285-299 | 36-51 | [98] | **4** | 300-500 | 300-500 | 6-10 | 302-334 | 28 | [99] |
| 17 | 101-143 | 101-143 | 2-5.1 | 255-347 | 49-67 | [100] | **3** | 300 | 300 | 2-6 | 177-356 | 53 | [101] |
| 10 | 106-160 | 106-140 | 4 | 495 | 60-89 | [102] | **1** | 200 | 200 | 5.6 | 397 | 112 | [103] |
| 2 | 129-250 | 128-249 | 2.5 | 234 | 51-53 | [104] | **2** | 130 | 130 | 5 | 1031 | 76-125 | [105] |
| 3 | 120-180 | 120-180 | 1.5 | 223 | 48-49 | [8] | **22** | 60-250 | 60-250 | 1.9-2 | 282-404 | 43-72 | [41] |
| 6 | 80-149 | 80-149 | 1.5-3.6 | 280-284 | 34-45 | [106] | **4** | 110-160 | 110-160 | 5 | 750 | 28-30 | [107] |
| 6 | 190-250 | 190-250 | 2.5 | 270-342 | 50-58 | [108] | **2** | 120-170 | 120-170 | 5 | 761 | 20 | [109] |
| 3 | 160-280 | 160-280 | 2.5 | 202-221 | 33-39 | [110] | **19** | 80-162 | 51-162 | 3.9-5 | 629-1022 | 36-115 | [111] |
| 4 | 410-500 | 410-500 | 10-16 | 358-389 | 43 | [112] | **4** | 200 | 200 | 4-6 | 293 | 25-40 | [113] |
| 8 | 100-318 | 100-201 | 4.4-5.99 | 310-733 | 40-92 | [114] | **1** | 200 | 200 | 4 | 293 | 36 | [115] |
| 1 | 92 | 92 | 3 | 420 | 19 | [53] | **9** | 120 | 120 | 2.5-4 | 268-274 | 23-39 | [116] |
| 2 | 151 | 151 | 3.55 | 341.7 | 29 | [117] | **2** | 100 | 100 | 2.8 | 361.6 | 71.8 | [66] |
| 3 | 250-350 | 250-350 | 4-6 | 351-498 | 29 | [118] | **1** | 150 | 150 | 3.5 | 341.7 | 30.88 | [119] |
| 11 | 99-138 | 98-140 | 5.09-12.04 | 400-730 | 88 | [120] | **8** | 200-1001 | 200-1001 | 3.68-20.35 | 261-279 | 46 | [121] |
| 1 | 130 | 130 | 3 | 737 | 78 | [122] | **1** | 120 | 120 | 2.75 | 398 | 47 | [123] |
| 1 | 158 | 158 | 3 | 330 | 23 | [124] | **4** | 200-300 | 200 | 2.973 | 732 | 31-86 | [125] |
|  |  |  |  |  |  |  | **396** | **60-500** | **51-500** | **0.7-16** | **177-1031** | **12-158** | **Total** |

Table 3: Summary of concrete-filled double-skin circular steel tubular (CFDST) stub column test data.

| $\boldsymbol{n}$ | $\boldsymbol{D}$ (mm) | $\boldsymbol{t}$ (mm) | $\boldsymbol{D}_{\boldsymbol{i}}$ (mm) | $\boldsymbol{t}_{\boldsymbol{i}}$ (mm) | $\boldsymbol{f}_{\boldsymbol{y}}$ (MPa) | $\boldsymbol{f}_{\boldsymbol{yi}}$ (MPa) | $\boldsymbol{f}_{\boldsymbol{c}}^{\boldsymbol{'}}$ (MPa) | Ref. |
| --- | --- | --- | --- | --- | --- | --- | --- | --- |
| 26 | 75-114 | 0.6-1.8 | 61-89 | 0.6-1.6 | 255-524 | 216-512 | 59 | [126] |
| 12 | 114-300 | 3 | 48-165 | 3 | 276-295 | 295-396 | 47 | [127] |
| 14 | 114-165 | 1.7-6 | 48-102 | 2.8-3.3 | 395-454 | 394-425 | 63 | [128] |
| 5 | 160 | 1-2.1 | 75-112 | 1-2.1 | 220-300 | 220-300 | 24 | [129] |
| 4 | 240 | 3-4 | 80-120 | 3-4 | 280 | 280 | 29 | [130] |
| 9 | 157-159 | 0.9-2.1 | 38-115 | 0.9-2.1 | 221-308 | 221-308 | 19 | [131] |
| 2 | 350 | 3.8 | 231 | 2.9 | 439 | 397 | 44 | [132] |
| 5 | 139 | 2 | 75 | 3 | 250 | 250 | 47 | [133] |
| 6 | 114 | 2 | 48-89 | 1.6 | 279 | 235 | 40 | [134] |
| 19 | 102-203 | 1.6-3.2 | 50-114 | 1.5-3.2 | 226-353 | 226-399 | 40 | [135] |
| 23 | 140-166 | 2.9 | 22-89 | 3.9-10.8 | 276-300 | 433-1029 | 41-116 | [136] |
| 4 | 356 | 5.5 | 168-219 | 3.3 | 618 | 356-357 | 39 | [137] |
| 8 | 114 | 2.7-5.9 | 60 | 2.5-5.8 | 285-455 | 310-396 | 39-64 | [138] |
| 8 | 114 | 2.7-6.1 | 60 | 2.5-5.8 | 355-535 | 310-396 | 41-68 | [139] |
| 24 | 188-191 | 4.2-6.8 | 34-102 | 3.1-4.1 | 327-464 | 342-348 | 29-51 | [140] |
| 24 | 494-496 | 165 | 3.7-6 | 43-76 | 3 | 347-429 | 386-410 | [141] |
| 2 | 300 | 2-4 | 180 | 2 | 250 | 250 | 28 | [142] |
| 8 | 140-180 | 3-5 | 48-78 | 3-5 | 285-320 | 285-320 | 35 | [143] |
| 4 | 300 | 6 | 100-135 | 4 | 763 | 748 | 89 | [144] |
| 2 | 170 | 3.58 | 76-114 | 3.58 | 365 | 320-380 | 35 | [145] |
| 12 | 189-191 | 4.26-6.77 | 33.7-101.6 | 3.08-4.1 | 327-464 | 342-348 | 29-51 | [65] |
| 2 | 121 | 4.37-4.43 | 58 | 4.03 | 4.86 | 450 | 39-52 | [146] |
| 3 | 200 | 2.74 | 60-140 | 2.74 | 359 | 359 | 36-58 | [147] |
| 3 | 140-180 | 3 | 48-102 | 3 | 276-342 | 321-396 | 37 | [148] |
| 16 | 86-114 | 1-2.5 | 39-41 | 1-2 | 345 | 345 | 9.8-22 | [149], [150] |
| 195 | **75-356** | **0.6-6.8** | **22-231** | **0.6-10.8** | **220-618** | **216-1029** | **19-141** | **Total** |

References:

[1] E. R. Gardner, N. J. and Jacobson, “Structural Behavior of Concrete Filled Steel Tubes,” *urnal Am. Concr. Inst.*, vol. 64, no. 11, pp. 404–413, 1967.

[2] G. Giakoumelis and D. Lam, “Axial capacity of circular concrete-filled tube columns,” *J. Constr. Steel Res.*, vol. 60, no. 7, pp. 1049–1068, 2004, doi: 10.1016/j.jcsr.2003.10.001.

[3] N. J. Gardner, “Use of Spiral Welded Steel Tubes in Pipe Columns,” *J. Am. Concr. Inst.*, vol. 65, no. 11, pp. 937–942, 1968.

[4] L. H. Han and G. H. Yao, “Experimental behaviour of thin-walled hollow structural steel (HSS) columns filled with self-consolidating concrete (SCC),” *Thin-Walled Struct.*, vol. 42, no. 9, pp. 1357–1377, 2004, doi: 10.1016/j.tws.2004.03.016.

[5] R. Knowles, R. B. and Park, “Strength of Concrete Filled Steel Tubular Columns,” *J. Struct. Div. ASCE*, vol. 95, no. 12, pp. 2565–2587, 1969.

[6] K. Sakino, H. Nakahara, S. Morino, and I. Nishiyama, “Behavior of Centrally Loaded Concrete-Filled Steel-Tube Short Columns,” *J. Struct. Eng.*, vol. 130, no. 2, pp. 180–188, 2004, doi: 10.1061/(asce)0733-9445(2004)130:2(180).

[7] Y. C. Zhong, S. T., and Wang, “Discussion on the behavior and load-bearing capacity of axially loaded concrete-filled steel tubular columns,” *J. Harbin Inst. Archit. Eng.*, 1978.

[8] B. Z. Zhang, Y. C., Wang Q. P., Mao, X. Y., and Cao, “Research on Mechanics Behavior of Stub-column of Concrete-filled Thin-walled Steel Tube Under Axial Load,” *Build. Struct.*, vol. 35, no. 1, pp. 22–27, 2005.

[9] X. M. Tang, G. Z., Zhao, B. Q., Zhu, H. X., and Shen, “Study on the fundamental structural behaviour of concrete filled steel tubular columns,” *J. Build. Struct.*, vol. 1, pp. 13–31, 1982.

[10] Z. wu Yu, F. xing Ding, and C. S. Cai, “Experimental behavior of circular concrete-filled steel tube stub columns,” *J. Constr. Steel Res.*, vol. 63, no. 2, pp. 165–174, 2007, doi: 10.1016/j.jcsr.2006.03.009.

[11] R. Q. Zhong, S. T., and He, “Research on load-bearing capacity of slender concrete-filled steel tubular column under axial compression,” *J. Harbin Inst. Archit. Eng.*, vol. 1, pp. 1–13, 1983.

[12] Q. Yu, Z. Tao, and Y. X. Wu, “Experimental behaviour of high performance concrete-filled steel tubular columns,” *Thin-Walled Struct.*, vol. 46, no. 4, pp. 362–370, 2008, doi: 10.1016/j.tws.2007.10.001.

[13] S. T. Zhong, “Research on the Clamping force of eccentrically loaded Concrete filled steel Tube members,” *J. Harbin Inst. Archit. Eng.*, vol. 3, pp. 1–18, 1983.

[14] X. Chang, L. Fu, H. B. Zhao, and Y. Bin Zhang, “Behaviors of axially loaded circular concrete-filled steel tube (CFT) stub columns with notch in steel tubes,” *Thin-Walled Struct.*, vol. 73, pp. 273–280, 2013, doi: 10.1016/j.tws.2013.08.018.

[15] C. S. J. Zhanshuan, “Behavior and ultimate strength of short concrete-filled steel tubular columns,” *J. Build. Struct.*, vol. 5, no. 06, p. 13, 1984.

[16] S. Guler, A. C. Çopur, and M. Aydogan, “Axial capacity and ductility of circular UHPC-filled steel tube columns,” *Mag. Concr. Res.*, vol. 65, no. 15, pp. 898–905, 2013, doi: 10.1680/macr.12.00211.

[17] W. L. Cai, S. H. and Gu, “Behavior and Ultimate Strength of Long Concrete-Filled Steel Tubular Columns,” *J. Build. Struct.*, vol. 4, pp. 32–42, 1985, doi: 10.14006/j.jzjgxb.1985.01.003.

[18] T. Ekmekyapar and B. J. M. Al-Eliwi, “Experimental behaviour of circular concrete filled steel tube columns and design specifications,” *Thin-Walled Struct.*, vol. 105, pp. 220–230, 2016, doi: 10.1016/j.tws.2016.04.004.

[19] K. Sakino, K., Tomii, M., and Watanabe, “Sustaining Load Capacity of Plain Concrete Stub Columns Confined by Circular Steel Tube,” in *Proceedings 1st ASCCS International Conference on Steel-Concrete Composite Structures, Harbin, China*, 1985, pp. 112–118.

[20] Y. Ye, L. H. Han, T. Sheehan, and Z. X. Guo, “Concrete-filled bimetallic tubes under axial compression: Experimental investigation,” *Thin-Walled Struct.*, vol. 108, pp. 321–332, 2016, doi: 10.1016/j.tws.2016.09.004.

[21] C. Y. Lin, “Axial capacity of concrete infilled cold-formed steel columns,” *Int. Speacialty Conf. Cold-Formed Steel Struct.*, pp. 443–457, 1988, [Online]. Available: http://scholarsmine.mst.edu/isccsshttp://scholarsmine.mst.edu/isccss/9iccfss-session1/9iccfss-session5/2.

[22] L. Zhu *et al.*, “Large diameter concrete-filled high strength steel tubular stub columns under compression,” *Thin-Walled Struct.*, vol. 108, pp. 12–19, 2016, doi: 10.1016/j.tws.2016.08.004.

[23] A. P. Luksha, L. K. and Nesterovich, “Strength of Tubular Concrete Cylinders under Combined Loading,” in *Proceedings of 3rd International Conference on Steel-Concrete Composite Structures, Fukuoka, Japan*, 1991, pp. 67–71.

[24] Y. Wang, P. Chen, C. Liu, and Y. Zhang, “Size effect of circular concrete-filled steel tubular short columns subjected to axial compression,” *Thin-Walled Struct.*, vol. 120, no. September, pp. 397–407, 2017, doi: 10.1016/j.tws.2017.09.010.

[25] H. Sakino, K. and Hayashi, “Behavior of Concrete Filled Steel Tubular Stub Columns under Concentric Loading,” in *Proceedings of 3rd International Conference on Steel-Concrete Composite Structures, Fukuoka, Japan*, 1991, pp. 25–30.

[26] M. X. Xiong, D. X. Xiong, and J. Y. R. Liew, “Axial performance of short concrete filled steel tubes with high- and ultra-high- strength materials,” *Eng. Struct.*, vol. 136, pp. 494–510, 2017, doi: 10.1016/j.engstruct.2017.01.037.

[27] R. Bergmann, “Load introduction in composite columns filled with high strength concrete,” in *Tubular Structures VI, Proceedings of 6th International Symposium on Tubular Structures, Melbourne, Australia*, 1994, pp. 373–380.

[28] C. Ibañez, D. Hernández-Figueirido, and A. Piquer, “Shape effect on axially loaded high strength CFST stub columns,” *J. Constr. Steel Res.*, vol. 147, pp. 247–256, 2018, doi: 10.1016/j.jcsr.2018.04.005.

[29] H. G. L. Prion and J. Boehme, “Beam-column behaviour of steel tubes filled with high strength concrete,” *Can. J. Civ. Eng.*, vol. 21, no. 2, pp. 207–218, Apr. 1994, doi: 10.1139/l94-024.

[30] J. Wei, X. Luo, Z. Lai, and A. H. Varma, “Experimental Behavior and Design of High-Strength Circular Concrete-Filled Steel Tube Short Columns,” *J. Struct. Eng.*, vol. 146, no. 1, 2020, doi: 10.1061/(asce)st.1943-541x.0002474.

[31] L. H. Han, “Theoretical analysis and experimental researchs for the behaviors of high strength concrete filled steel tubes subjected to axial compression,” *Ind. Constr.*, vol. 27, no. 11, pp. 39–44, 1997.

[32] S. Chen, R. Zhang, L. J. Jia, J. Y. Wang, and P. Gu, “Structural behavior of UHPC filled steel tube columns under axial loading,” *Thin-Walled Struct.*, vol. 130, no. June, pp. 550–563, 2018, doi: 10.1016/j.tws.2018.06.016.

[33] M. D. O’Shea and R. Q. Bridge, “Tests of thin-walled concrete-filled steel tubes,” *Int. Spec. Conf. Cold-Formed Steel Struct. Recent Res. Dev. Cold-Formed Steel Des. Constr.*, pp. 399–419, 1994.

[34] Y. Cai, A. K. H. Kwan, and L. G. Li, “Circular concrete filled steel tubes made of eco-concrete with limestone fines added as cementitious paste replacement,” *Structures*, vol. 28, no. June, pp. 69–79, 2020, doi: 10.1016/j.istruc.2020.08.044.

[35] K. F. Tan, X. C. Pu, and S. H. Cai, “Study on the mechanical properties of steel extra-high strength concrete encased in steel tubes,” *J. Build. Struct.*, vol. 20, no. 1, pp. 10–15, 1999.

[36] O. M. D. and B. R. Q., “Design of Circular Thin-Walled Concrete Filled Steel Tubes,” *J. Struct. Eng.*, vol. 126, no. 11, pp. 1295–1303, Nov. 2000, doi: 10.1061/(ASCE)0733-9445(2000)126:11(1295).

[37] B. KATO, “COMPRESSIVE STRENGTH AND DEFORMATION CAPACITY OF CONCRETE-FILLED TUBULAR STUB-COLUMNS : Strength and Rotation Capactity of Concrete-filled Tubular Columns, Part 1,” *J. Struct. Constr. Eng. (Transactions AIJ)*, vol. 60, no. 468, pp. 183–191, 1995, doi: 10.3130/aijs.60.183.

[38] T. Yamamoto, J. Kawaguchi, and S. Morino, “Experimental study of scale effects on the compressive behavior of short concrete-filled steel tube columns,” *Proc. Conf. Compos. Constr. Steel Concr. IV*, pp. 879–890, 2000, doi: 10.1061/40616(281)76.

[39] Y. Lu, N. Li, S. Li, and H. Liang, “Behavior of steel fiber reinforced concrete-filled steel tube columns under axial compression,” *Constr. Build. Mater.*, vol. 95, pp. 74–85, 2015, doi: 10.1016/j.conbuildmat.2015.07.114.

[40] L. H. Han and G. H. Yao, “Behaviour of concrete-filled hollow structural steel (HSS) columns with pre-load on the steel tubes,” *J. Constr. Steel Res.*, vol. 59, no. 12, pp. 1455–1475, 2003, doi: 10.1016/S0143-974X(03)00102-0.

[41] L. H. Han, G. H. Yao, and X. L. Zhao, “Tests and calculations for hollow structural steel (HSS) stub columns filled with self-consolidating concrete (SCC),” *J. Constr. Steel Res.*, vol. 61, no. 9, pp. 1241–1269, 2005, doi: 10.1016/j.jcsr.2005.01.004.

[42] K. Uenaka, K., Hayami, M., Kitoh, H., and Sonoda, “Experimental Study on Concrete Filled Double Tubular Steel Columns under Axial Loading,” in *ASSCCA’03 International Conference Advances in Structures (ASCCS-7), Sydney, Australia*, 2003, pp. 877–882.

[43] S. M. I. Shah and G. M. Ganesh, “Experimental and numerical study on the effect of parameters in axial capacity of CFST columns with various L/D ratios,” *Int. J. Adv. Technol. Eng. Explor.*, vol. 9, no. 93, pp. 1209–1221, 2022, doi: 10.19101/IJATEE.2021.875190.

[44] Y. Q. Zeng, L. H. Xu, F. H. Wu, M. Yu, and Y. Chi, “Study on Axial Compression Behavior of Ca-Uhpc Filled Steel Tube Stub Columns,” *Gongcheng Lixue/Engineering Mech.*, vol. 39, no. 10, pp. 68–78, 2022, doi: 10.6052/j.issn.1000-4750.2021.05.0354.

[45] A. Karimi Pour, A. Shirkhani, N. Safaeian Hamzehkolaei, Y. Zhuge, and E. Noroozinejad Farsangi, “Performance evaluation of composite concrete-filled steel tube columns by steel fibers and different cross-section shapes: Experimental and numerical investigations,” *J. Constr. Steel Res.*, vol. 200, no. October 2022, p. 107656, 2023, doi: 10.1016/j.jcsr.2022.107656.

[46] S. M. I. Shah and G. M. Ganesh, “Impact of diameter to thickness (D/t) on axial capacity of circular CFST columns: Experimental, parametric and numerical analysis,” *Int. J. Appl. Sci. Eng.*, vol. 19, no. 2, 2022, doi: 10.6703/IJASE.202206_19(2).005.

[47] H. Singh and A. K. Tiwary, “Influence of Diagonal Prop Bar on the Behavior of Stiffened Concrete-Filled Steel Tube Columns,” *J. Inst. Eng. Ser. A*, 2023, doi: 10.1007/s40030-023-00770-5.

[48] Y. Chen, Z. Lin, F. Liao, Z. Lin, W. Zhang, and Z. Chen, “Experimental Research on Axial Compression Performance of Circular Steel Tubular Stub Columns Filled with Geopolymer Recycled Concrete,” *Prog. Steel Build. Struct.*, vol. 24, no. 8, pp. 61–69, 2022, doi: 10.13969/j.cnki.cn31-1893.2022.08.006.

[49] J. Liu, P. Gao, X. Lin, X. Wang, X. Zhou, and Y. F. Chen, “Experimental assessment on the size effects of circular concrete-filled steel tubular columns under axial compression,” *Eng. Struct.*, vol. 275, no. PA, p. 115247, 2023, doi: 10.1016/j.engstruct.2022.115247.

[50] Y. F. Yang, Y. Q. Zhang, and F. Fu, “Behaviour of axially compressed CTHST stub columns with inner spiral stirrup,” *Structures*, vol. 45, no. September, pp. 372–389, 2022, doi: 10.1016/j.istruc.2022.09.038.

[51] B. Liu, L. Zhang, M. Feng, H. Sun, and Y. Chai, “Experimental Study of Rubber-Concrete-Filled CST Composite Column Under Axial Compression,” *Int. J. Steel Struct.*, vol. 23, no. 1, pp. 247–262, 2023, doi: 10.1007/s13296-022-00692-1.

[52] H. Zhao, R. Han, W. Yuan, S. Zhao, and Y. Sun, “Elastoplastic Analysis of Circular Steel Tube of CFT Stub Columns under Axial Compression,” *Materials (Basel).*, vol. 15, no. 22, 2022, doi: 10.3390/ma15228275.

[53] M. L. Patton, S. B. F. Warsi, and D. Adak, “Experimental and numerical study on the structural behaviour of HST, RCC and CFST stub columns under pure axial compression,” *Innov. Infrastruct. Solut.*, vol. 8, no. 2, pp. 1–13, 2023, doi: 10.1007/s41062-022-01025-1.

[54] Z. Zhang *et al.*, “Experimental and Numerical Study of the Behavior of Concrete-Filled High-Strength Steel Tube Columns with Large D/t Ratio under Axial Compression,” *Buildings*, vol. 12, no. 11, p. 1953, 2022, doi: 10.3390/buildings12111953.

[55] M. H. Lai, Y. H. Lin, Y. Y. Jin, Q. Fei, Z. C. Wang, and J. C. M. Ho, “Uni-axial behaviour of steel slag concrete-filled-steel-tube columns with external confinement,” *Thin-Walled Struct.*, vol. 185, no. September 2022, 2023, doi: 10.1016/j.tws.2023.110562.

[56] H. Huang, L. Guo, O. Zhao, and S. Gao, “Experimental and numerical investigation into locally corroded circular concrete-filled steel tubular stub columns strengthened by CFRP,” *Thin-Walled Struct.*, vol. 192, no. April, p. 111174, 2023, doi: 10.1016/j.tws.2023.111174.

[57] Y. Diao, L. Chen, and Y. Huang, “Experimental Study on Mechanical Properties of Concrete Containing Waste Glass and Its Application on Concrete-Filled Steel Tubular Columns,” *Processes*, vol. 11, no. 4, 2023, doi: 10.3390/pr11040975.

[58] C. Xue, M. Yu, M. Wu, and S. Cheng, “Axial compressive behaviour of ultra-high performance concrete filled steel tube stub columns at elevated temperatures,” *Structures*, vol. 57, no. July, p. 105333, 2023, doi: 10.1016/j.istruc.2023.105333.

[59] T. Ghanbari-Ghazijahani, M. G. Azandariani, V. Vimonsatit, and N. H. R. Sulong, “Experiments and design of concrete-filled steel tubes with timber chips under axial compression,” *Thin-Walled Struct.*, vol. 186, no. March, p. 110679, 2023, doi: 10.1016/j.tws.2023.110679.

[60] S. Zhang, K. Miao, Y. Wei, X. Xu, B. Luo, and W. Shi, “Experimental and Theoretical Study of Concrete-Filled Steel Tube Columns Strengthened by FRP/Steel Strips Under Axial Compression,” *Int. J. Concr. Struct. Mater.*, vol. 17, no. 1, pp. 1–22, 2023, doi: 10.1186/s40069-022-00556-2.

[61] B. Cheng, W. Wang, J. Li, J. Huang, and H. Chen, “Mechanical Properties of Full-Scale UHPC-Filled Steel Tube Composite Columns under Axial Load,” *Materials (Basel).*, vol. 16, no. 13, 2023, doi: 10.3390/ma16134860.

[62] F. Wu, L. Xu, Y. Zeng, M. Yu, and B. Li, “Behavior of CA-UHPC filled circular steel tube stub columns under axial compression,” *J. Constr. Steel Res.*, vol. 211, no. June, p. 108204, 2023, doi: 10.1016/j.jcsr.2023.108204.

[63] S. M. I. Shah and G. M. Ganesh, “Micro-Steel Fiber-Reinforced Self-compacting Concrete-Filled Steel-Tube Columns Subjected to Axial Compression,” *Int. J. Steel Struct.*, vol. 23, no. 4, pp. 1031–1045, 2023, doi: 10.1007/s13296-023-00747-x.

[64] H. Jiang, Y. Ye, and S. Y. Lai, “Behavior of seawater sea sand concrete-filled plastic-lined steel tube stub columns under axial compression,” *Structures*, vol. 58, no. November, p. 105577, 2023, doi: 10.1016/j.istruc.2023.105577.

[65] X. F. Yan, S. Lin, and M. He, “Comparative Study on Behavior of Circular Axially Loaded CFDST Short Columns under Different Loading Arrangements,” *Buildings*, vol. 13, no. 8, pp. 1–17, 2023, doi: 10.3390/buildings13082054.

[66] M. Elzeadani, D. V. Bompa, and A. Y. Elghazouli, “Axial compressive behaviour of composite steel elements incorporating rubberised alkali-activated concrete,” *J. Constr. Steel Res.*, vol. 212, no. August 2023, p. 108276, 2024, doi: 10.1016/j.jcsr.2023.108276.

[67] Z. L. Li, S. Q. Lin, Y. G. Zhao, and W. Da Wang, “Experimental Study on Behavior of Axially Loaded Concrete-Filled Steel Tube Stub Columns With Local Corrosion,” *Gongcheng Lixue/Engineering Mech.*, vol. 40, no. 8, pp. 170–180, 2023, doi: 10.6052/j.issn.1000-4750.2021.12.1003.

[68] B. Wei, Y. Wei, Y. Lin, G. Wang, and Y. Zhang, “Compressive performance of bamboo scrimber and concrete-filled steel tube columns,” *Eng. Struct.*, vol. 300, no. November 2023, p. 117192, 2024, doi: 10.1016/j.engstruct.2023.117192.

[69] X. Lyu, T. Zhang, W. Wang, L. Zhang, and G. Xue, “Behavior of concrete-filled circular steel tubular stub columns exposed to corrosion and freeze–thaw cycles,” *Structures*, vol. 55, no. June, pp. 2266–2279, 2023, doi: 10.1016/j.istruc.2023.07.029.

[70] K. Miao, Y. Wei, F. Dong, K. Zheng, and J. Wang, “Experimental study on concrete-filled steel tube columns with inner distributed seawater and sea sand concrete-filled fiber-reinforced polymer tubes under axial compression,” *Compos. Struct.*, vol. 320, no. October 2021, p. 117181, 2023, doi: 10.1016/j.compstruct.2023.117181.

[71] H. Singh and A. K. Tiwary, “Experimental and numerical investigation on concrete filled steel tube columns reinforced with diagonal stiffeners under axial loading,” *Eng. Struct.*, vol. 292, no. June, p. 116602, 2023, doi: 10.1016/j.engstruct.2023.116602.

[72] A. K. Tiwary and S. Bhatia, “Experimental Investigation on Post-fire Response of Concrete-Filled Steel Tube Column Subjected to Elevated Temperature,” *Fire Technol.*, vol. 59, no. 2, pp. 847–878, 2023, doi: 10.1007/s10694-023-01368-w.

[73] A. Xiamuxi, A. Aosimanjiang, and B. Yang, “Loading performance of reinforced and recycled aggregate concrete-filled circular steel tube short column with different steel ratios,” *Constr. Build. Mater.*, vol. 399, no. March, p. 132486, 2023, doi: 10.1016/j.conbuildmat.2023.132486.

[74] Y. Zhaoyuan, Q. Qiyun, C. Wanlin, and L. Jiafeng, “Study on axial compressive behavior of circular steel tube confined rubberized concrete stub columns,” *Structures*, vol. 41, no. March, pp. 887–907, 2022, doi: 10.1016/j.istruc.2022.05.022.

[75] C. Hui, Y. Li, K. Li, C. Liu, R. Hai, and C. Li, “Experimental investigation and analysis on the axial compressive performance of recycled concrete-filled corroded steel tubular columns,” *Arch. Civ. Mech. Eng.*, vol. 22, no. 2, 2022, doi: 10.1007/s43452-022-00422-8.

[76] C. Hui, K. Li, Y. Li, Y. Bian, R. Hai, and C. Li, “Experimental Study and Analysis on Axial Compression Performance of High-Strength Recycled Concrete-Filled Steel Tube Column in Corrosive Environments,” *Int. J. Steel Struct.*, vol. 22, no. 2, pp. 450–471, 2022, doi: 10.1007/s13296-022-00584-4.

[77] J. Wang, W. J. Qiu, S. C. Kong, and J. H. Zhu, “Investigation of the axial compressive behaviour of CFRP-confined circular CFST stub columns with inner latticed steel angles,” *Compos. Struct.*, vol. 280, no. October 2021, p. 114895, 2022, doi: 10.1016/j.compstruct.2021.114895.

[78] S. Ahmad, K. Kumar, and A. Kumar, “Axial behaviour of steel tubes filled with concrete incorporating high-volume rubber,” *Innov. Infrastruct. Solut.*, vol. 7, no. 2, pp. 1–11, 2022, doi: 10.1007/s41062-022-00739-6.

[79] M. Shen, W. Huang, J. Liu, and Z. Zhou, “Axial compressive behavior of rubberized concrete-filled steel tube short columns,” *Case Stud. Constr. Mater.*, vol. 16, no. November 2021, p. e00851, 2022, doi: 10.1016/j.cscm.2021.e00851.

[80] M. Tomii and K. Sakino, “EXPERIMENTAL STUDIES ON THE ULTIMATE MOMENT OF CONCRETE FILLED SQUARE STEEL TUBULAR BEAM-COLUMNS,” *Trans. Archit. Inst. Japan*, vol. 275, pp. 55–65, 1979.

[81] F. X. Ding, C. Fang, Y. Bai, and Y. Z. Gong, “Mechanical performance of stirrup-confined concrete-filled steel tubular stub columns under axial loading,” *J. Constr. Steel Res.*, vol. 98, pp. 146–157, 2014, doi: 10.1016/j.jcsr.2014.03.005.

[82] Y. Liu, *Research on mechanical behavior of high-strength concrete-filled high-strength square steel tubular stub columns under axial load (Master dissertation), Department of Civil Engineering, Shenyang Jianzhu University, Shenyang, China, December.* 2014.

[83] M. Shakir-Khalil, H. and Mouli, “Further Tests on Concrete-Filled Rectangular Hollow-Section Columns,” *Struct. Eng.*, vol. 68, no. 20, pp. 405–413, 1990.

[84] J. G. Liu, Y. J., Cheng, G., Zhang, N., and Zhang, “Experimental research on concrete-filled square steel tubular columns stiffened with PBL,” *J. Build. Struct.*, vol. 35, no. 10, pp. 39–46, 2014.

[85] M. Grauers, “Composite Columns of Hollow Steel Sections Filled with High Strength Concrete,” 1993.

[86] Y. Yang, Y. Wang, and F. Fu, “Effect of reinforcement stiffeners on square concrete-filled steel tubular columns subjected to axial compressive load,” *Thin-Walled Struct.*, vol. 82, pp. 132–144, 2014, doi: 10.1016/j.tws.2014.04.009.

[87] Varma, *Seismic Behavior, Analysis, and Design of High Strength Square Concrete Filled Steel Tube (CFT) Columns (Ph.D. dissertation), Department of Civil Engineering, Lehigh University, Bethlehem, Pennsylvania*. 2000.

[88] F. Aslani, B. Uy, Z. Tao, and F. Mashiri, “Behaviour and design of composite columns incorporating compact high-strength steel plates,” *J. Constr. Steel Res.*, vol. 107, pp. 94–110, 2015, doi: 10.1016/j.jcsr.2015.01.005.

[89] Y. Du, Z. Chen, and M. Xiong, “Experimental behavior and design method of rectangular concrete-filled tubular columns using Q460 high-strength steel,” *Constr. Build. Mater.*, vol. 125, pp. 856–872, 2016, doi: 10.1016/j.conbuildmat.2016.08.057.

[90] Z. Han, L.-H., Zhao, X.-L., and Tao, “Tests and mechanics model for concrete-filled SHS stub columns, columns and beam-columns,” *Steel Compos. Struct.*, vol. 1, no. 1, pp. 51–74, 2001.

[91] F. xing Ding, T. Zhang, X. mei Liu, Z. H. Lu, Q. Guo, and G. shuai Jiang, “Behavior of steel-reinforced concrete-filled square steel tubular stub columns under axial loading,” *Thin-Walled Struct.*, vol. 119, no. July, pp. 737–748, 2017, doi: 10.1016/j.tws.2017.07.021.

[92] Z. L. Ye, *Research on the fundamental behavior of high-strength concrete-filled square and rectangular steel tubes under axial load (Ph.D. Dissertation), Harbin Institute of Technology, Harbin, China*. 2001.

[93] M. Khan, B. Uy, Z. Tao, and F. Mashiri, “Behaviour and design of short high-strength steel welded box and concrete-filled tube (CFT) sections,” *Eng. Struct.*, vol. 147, pp. 458–472, 2017, doi: 10.1016/j.engstruct.2017.06.016.

[94] L. H. Han, “Tests on stub columns of concrete-filled RHS sections,” *J. Constr. Steel Res.*, vol. 58, no. 3, pp. 353–372, 2002, doi: 10.1016/S0143-974X(01)00059-1.

[95] D. Liu, W. M. Gho, and J. Yuan, “Ultimate capacity of high-strength rectangular concrete-filled steel hollow section stub columns,” *J. Constr. Steel Res.*, vol. 59, no. 12, pp. 1499–1515, 2003, doi: 10.1016/S0143-974X(03)00106-8.

[96] A. Zhu, X. Zhang, H. Zhu, J. Zhu, and Y. Lu, “Experimental study of concrete filled cold-formed steel tubular stub columns,” *J. Constr. Steel Res.*, vol. 134, pp. 17–27, 2017, doi: 10.1016/j.jcsr.2017.03.003.

[97] F. xing Ding, L. Luo, J. Zhu, L. Wang, and Z. wu Yu, “Mechanical behavior of stirrup-confined rectangular CFT stub columns under axial compression,” *Thin-Walled Struct.*, vol. 124, no. June 2017, pp. 136–150, 2018, doi: 10.1016/j.tws.2017.12.007.

[98] Shang, *Study on behavior of square concrete filled steel tubular beams and columns and square concrete with reinforce filled steel tubular beams and columns (M.S. Thesis), School of Architecture and Civil Engineering, Shenyang University of Technology, Shenyan*. 2004.

[99] M. C. Wu, C. C. Chen, and C. C. Chen, “Size effect on axial behavior of concrete-filled box columns,” *Adv. Struct. Eng.*, vol. 21, no. 13, pp. 2068–2078, 2018, doi: 10.1177/1369433218766366.

[100] Y. Zhang, S., Guo, L., Ye, Z., and Wang, “Experimental research on high strength concrete-filled SHS stub columns subjected to axial compression load,” *J. Harbin Inst. Technol.*, vol. 36, no. 12, pp. 1610–1614, 2004.

[101] Z. Zhou, D. Gan, and X. Zhou, “Improved Composite Effect of Square Concrete-Filled Steel Tubes with Diagonal Binding Ribs,” *J. Struct. Eng.*, vol. 145, no. 10, pp. 1–12, 2019, doi: 10.1061/(asce)st.1943-541x.0002400.

[102] D. Liu, “Tests on high-strength rectangular concrete-filled steel hollow section stub columns,” *J. Constr. Steel Res.*, vol. 61, no. 7, pp. 902–911, 2005, doi: 10.1016/j.jcsr.2005.01.001.

[103] H.-S. Hu, H.-Z. Wang, Z.-X. Guo, and B. M. Shahrooz, “Axial Compressive Behavior of Square Spiral-Confined High-Strength Concrete-Filled Steel-Tube Columns,” *J. Struct. Eng.*, vol. 146, no. 7, pp. 1–11, 2020, doi: 10.1061/(asce)st.1943-541x.0002702.

[104] Z. Tao, L. H. Han, and Z. Bin Wang, “Experimental behaviour of stiffened concrete-filled thin-walled hollow steel structural (HSS) stub columns,” *J. Constr. Steel Res.*, vol. 61, no. 7, pp. 962–983, 2005, doi: 10.1016/j.jcsr.2004.12.003.

[105] Z. Huang, B. Uy, D. Li, and J. Wang, “Behaviour and design of ultra-high-strength CFST members subjected to compression and bending,” *J. Constr. Steel Res.*, vol. 175, p. 106351, 2020, doi: 10.1016/j.jcsr.2020.106351.

[106] L. H. Guo, *Theoretical and experimental research on the behavior of concrete-filled rectangular hollow section steel tubes (Ph.D. Dissertation), Harbin Institute of Technology, Harbin, China*. 2006.

[107] B. Uy, “Strength of short concrete filled high strength steel box columns,” *J. Constr. Steel Res.*, vol. 57, no. 2, pp. 113–134, 2001, doi: 10.1016/S0143-974X(00)00014-6.

[108] Z. Tao, L. H. Han, and D. Y. Wang, “Strength and ductility of stiffened thin-walled hollow steel structural stub columns filled with concrete,” *Thin-Walled Struct.*, vol. 46, no. 10, pp. 1113–1128, 2008, doi: 10.1016/j.tws.2008.01.007.

[109] M. Mursi and B. Uy, “Strength of slender concrete filled high strength steel box columns,” *J. Constr. Steel Res.*, vol. 60, no. 12, pp. 1825–1848, 2004, doi: 10.1016/j.jcsr.2004.05.002.

[110] M. C. Huang, H., Zhang, A. G., Li, Y., and Chen, “Experimental research and finite element analysis on mechanical performance of concrete-filled stiffened square steel tubular stub colunms subjected to axial compression,” *J. Build. Struct.*, vol. 32, no. 2, pp. 75–82, 2011.

[111] Y. Cai, M. Su, X. Chen, and B. Young, “High strength steel square and rectangular tubular stub columns in filled with concrete,” *J. Constr. Steel Res.*, vol. 179, p. 106536, 2021, doi: 10.1016/j.jcsr.2021.106536.

[112] C. C. Chen, J. W. Ko, G. L. Huang, and Y. M. Chang, “Local buckling and concrete confinement of concrete-filled box columns under axial load,” *J. Constr. Steel Res.*, vol. 78, pp. 8–21, 2012, doi: 10.1016/j.jcsr.2012.06.006.

[113] F. Yuan, L. Cao, and H. Li, “Axial compressive behaviour of high-strength steel spiral-confined square concrete-filled steel tubular columns,” *J. Constr. Steel Res.*, vol. 192, no. March, p. 107245, 2022, doi: 10.1016/j.jcsr.2022.107245.

[114] Y. Zhong and O. Zhao, “Concrete-filled high strength steel tube stub columns after exposure to fire: Testing, numerical modelling and design,” *Thin-Walled Struct.*, vol. 177, no. April, p. 109428, 2022, doi: 10.1016/j.tws.2022.109428.

[115] F. Yuan, H. Li, and H. Li, “Mechanical behavior of the sustainable square spiral stirrup-confined recycled aggregate concrete filled steel tubular columns under compression,” *J. Clean. Prod.*, vol. 426, no. March, p. 139099, 2023, doi: 10.1016/j.jclepro.2023.139099.

[116] W. Zhang *et al.*, “Axial compression behavior of square CFST short column with triangular-corner gap,” *J. Build. Eng.*, vol. 80, no. August, p. 108046, 2023, doi: 10.1016/j.jobe.2023.108046.

[117] L. Zhong, L. Guo, and C. Jia, “Axial compression behavior of stub square concrete-filled steel tubes with regional defect,” *Eng. Struct.*, vol. 278, no. June 2022, p. 115510, 2023, doi: 10.1016/j.engstruct.2022.115510.

[118] J. Wang, Z. M. Yang, X. L. Zheng, and Y. Ding, “Axial compression behavior of square section concrete-filled steel tubes reinforced with internal latticed steel angles,” *J. Constr. Steel Res.*, vol. 213, no. October 2023, p. 108414, 2024, doi: 10.1016/j.jcsr.2023.108414.

[119] C. Jia, J. Li, L. Guo, and L. Zhong, “Behavior of stub SCFST columns with localized pitting corrosion damage,” *Eng. Fail. Anal.*, vol. 156, no. November 2023, p. 107824, 2024, doi: 10.1016/j.engfailanal.2023.107824.

[120] P. Li, J. Jiang, Q. Li, and Z. Ren, “Axial compression performance and optimum design of round-cornered square CFST with high-strength materials,” *J. Build. Eng.*, vol. 68, no. February, 2023, doi: 10.1016/j.jobe.2023.106145.

[121] P. Gao, X. Zhou, J. Liu, X. Lin, X. Wang, and Y. Frank Chen, “Experimental assessment on the size effects of square concrete-filled steel tubular columns under axial compression,” *Eng. Struct.*, vol. 281, no. October 2022, p. 115706, 2023, doi: 10.1016/j.engstruct.2023.115706.

[122] Z. Lai, J. Yan, Y. Wang, C. Dong, and X. Weng, “Axial compressive behavior and design of high-strength square concrete-filled steel tube short columns with embedded GFRP tubes,” *J. Constr. Steel Res.*, vol. 207, no. April, p. 107955, 2023, doi: 10.1016/j.jcsr.2023.107955.

[123] J. Guo and Y. Diao, “Experimental behaviors of square concrete filled steel tubular columns with PBL stiffeners,” *Structures*, vol. 38, no. March, pp. 1556–1569, 2022, doi: 10.1016/j.istruc.2022.03.003.

[124] Q. Qiao, R. Ding, and W. Cao, “Experimental investigation on axial local compressive behavior of square concrete-filled double tubular stub columns,” *Structures*, vol. 55, no. June, pp. 2157–2174, 2023, doi: 10.1016/j.istruc.2023.07.005.

[125] Y. Du, D. Gao, Z. Chen, Z. Zheng, and X. Wang, “Behaviors of FRP confined rectangular concrete-filled thin-walled steel tubular stub columns using high-strength materials under axial load,” *Compos. Struct.*, vol. 280, no. October 2021, p. 114915, 2022, doi: 10.1016/j.compstruct.2021.114915.

[126] S. Wei, S. T. Mau, C. Vipulanandan, and S. K. Mantrala, “Performance of a new sandwich tube under axial loading,” *Proc. - 1994 Annu. Task Gr. Tech. Sess. Struct. Stab. Res.*, pp. 223–237, 1994.

[127] Z. Tao, L. H. Han, and X. L. Zhao, “Behaviour of concrete-filled double skin (CHS inner and CHS outer) steel tubular stub columns and beam-columns,” *J. Constr. Steel Res.*, vol. 60, no. 8, pp. 1129–1158, 2004, doi: 10.1016/j.jcsr.2003.11.008.

[128] X. L. Zhao, L. W. Tong, and X. Y. Wang, “CFDST stub columns subjected to large deformation axial loading,” *Eng. Struct.*, vol. 32, no. 3, pp. 692–703, 2010, doi: 10.1016/j.engstruct.2009.11.015.

[129] Y. Chen, J. Y., & Hai, “Research on Bearing Capacity of Short Concrete Filled Double Skin Steel Tubes Columns under Axial Compression,” *Adv. Mater. Res.*, vol. 168–170, pp. 2154–2157, 2010, doi: 10.4028/www.scientific.net/amr.168-170.2154.

[130] J. Fan, M. Baig, and J. Nie, “Test and analysis on double-skin concrete filled tubular columns,” *Tubul. Struct. XII Proc. Tubul. Struct. XII, Shanghai, China, 8-10 Oct. 2008*, vol. 407, 2008.

[131] K. Uenaka, H. Kitoh, and K. Sonoda, “Concrete filled double skin circular stub columns under compression,” *Thin-Walled Struct.*, vol. 48, no. 1, pp. 19–24, 2010, doi: 10.1016/j.tws.2009.08.001.

[132] W. Li, Q. X. Ren, L. H. Han, and X. L. Zhao, “Behaviour of tapered concrete-filled double skin steel tubular (CFDST) stub columns,” *Thin-Walled Struct.*, vol. 57, pp. 37–48, 2012, doi: 10.1016/j.tws.2012.03.019.

[133] H. Hastemoglu, “Behaviour of Double Skinned Composite Columns with Concrete Filled Tubular Columns,” *J. Archit. Eng. Technol.*, vol. 06, no. 02, 2017, doi: 10.4172/2168-9717.1000194.

[134] Wen XF., “Experimental and theoretical analysis of mechanical behaviour of concrete-filled the gap between stainless steel and steel tubular columns,” 2017.

[135] Y. L. Li, X. L. Zhao, R. K. Singh Raman, and X. Yu, “Axial compression tests on seawater and sea sand concrete-filled double-skin stainless steel circular tubes,” *Eng. Struct.*, vol. 176, no. March, pp. 426–438, 2018, doi: 10.1016/j.engstruct.2018.09.040.

[136] F. Wang, B. Young, and L. Gardner, “Compressive testing and numerical modelling of concrete- filled double skin CHS with austenitic stainless steel outer tubes,” *Thin Walled Struct.*, vol. 141, no. March, pp. 345–359, 2019, doi: 10.1016/j.tws.2019.04.003.

[137] W. Li and Y. X. Cai, “Performance of CFDST stub columns using high-strength steel subjected to axial compression,” *Thin-Walled Struct.*, vol. 141, no. April, pp. 411–422, 2019, doi: 10.1016/j.tws.2019.04.021.

[138] T. Ekmekyapar and H. Ghanim Hasan, “The influence of the inner steel tube on the compression behaviour of the concrete filled double skin steel tube (CFDST) columns,” *Mar. Struct.*, vol. 66, no. December 2018, pp. 197–212, 2019, doi: 10.1016/j.marstruc.2019.04.006.

[139] T. Ekmekyapar, O. H. Alwan, H. G. Hasan, B. A. Shehab, and B. J. M. AL-Eliwi, “Comparison of classical, double skin and double section CFST stub columns: Experiments and design formulations,” *J. Constr. Steel Res.*, vol. 155, pp. 192–204, 2019, doi: 10.1016/j.jcsr.2018.12.025.

[140] X. Yan and Y. Zhao, “Compressive strength of axially loaded circular concrete- filled double-skin steel tubular short columns,” *J. Constr. Steel Res.*, vol. 170, p. 106114, 2020, doi: 10.1016/j.jcsr.2020.106114.

[141] X. Yan, Y. Zhao, and S. Lin, “Compressive behaviour of circular CFDST short columns with high- and ultrahigh-strength concrete,” *Thin-Walled Struct.*, vol. 164, no. May, p. 107898, 2021, doi: 10.1016/j.tws.2021.107898.

[142] M. L. Lin and K.-C. Tsai, “Behavior of double-skinned composite steel tubular columns subjected to combined axial and flexural loads,” in *Proc. of the first international conference on the steel \& composite structures*, 2001, pp. 1145–1152.

[143] A. K. Tiwary, “Experimental investigation into mild steel circular concrete-filled double skin steel tube columns,” *J. Constr. Steel Res.*, vol. 198, no. September, p. 107527, 2022, doi: 10.1016/j.jcsr.2022.107527.

[144] W. Cancan *et al.*, “Study on the axial compression behavior of circular high strength concrete-filled double skin steel tubular members,” pp. 7823–7830, doi: 10.15986/j.1006-7930.2021.03.008.

[145] B. C. Cihan Yilmaz, E. Binbir, C. Guzelbulut, H. Yildirim, and O. C. Celik, “Circular concrete-filled double skin steel tubes under concentric compression: Tests and FEA parametric study,” *Compos. Struct.*, vol. 309, no. 79, p. 116765, 2023, doi: 10.1016/j.compstruct.2023.116765.

[146] H. Tang, J. Qin, H. Yang, L. Lang, Z. Gao, and X. Tang, “Analytical and experimental investigation on axial compression capacity of carbon fiber-reinforced plastic-confined concrete-filled double-skin steel tube stub columns,” *Adv. Struct. Eng.*, vol. 26, no. 13, pp. 2465–2488, 2023, doi: 10.1177/13694332231190710.

[147] X. Liu, Z. Liu, Y. Hui, and J. Wang, “Research on the Concrete-Filled Double Skin steel Tubular (CFDST) columns subjected to axial force after fire,” *Structures*, vol. 57, no. August, p. 105213, 2023, doi: 10.1016/j.istruc.2023.105213.

[148] H. Singh and A. K. Tiwary, “Axial compression behaviour of concrete filled double skin steel tube columns anchored with orbicular rings: Experimental and numerical approach,” *Adv. Struct. Eng.*, vol. 26, no. 15, pp. 2830–2861, 2023, doi: 10.1177/13694332231205054.

[149] S. T. N. Aghamaleki, M. Naghipour, J. V. Amiri, and M. Nematzadeh, “Compression behavior of the concrete-filled double skin steel tube columns under hydrostatic pressure: Experimental and modeling study,” *Structures*, vol. 58, no. November, p. 105505, 2023, doi: 10.1016/j.istruc.2023.105505.

[150] S. T. Nemati Aghamaleki, M. Naghipour, J. Vaseghi Amiri, and M. Nematzadeh, “Experimental Study on Compressive Behavior of Concrete-filled Double-skin Circular Tubes with Active Confinement,” *Int. J. Eng. Trans. A Basics*, vol. 35, no. 4, pp. 819–829, 2022, doi: 10.5829/ije.2022.35.04a.22.
