# Supplementary material for: Prediction of the axial compression capacity of stub CFST columns using machine learning techniques
Source: Sci Rep. 2024 Feb 5;14:2885. doi: 10.1038/s41598-024-53352-1 (PMC10838919; doi:10.1038/s41598-024-53352-1)
Supplement: Supplementary file 1 — Supplementary Information. [file 41598_2024_53352_MOESM1_ESM.zip › supplementary data/ML results.docx]

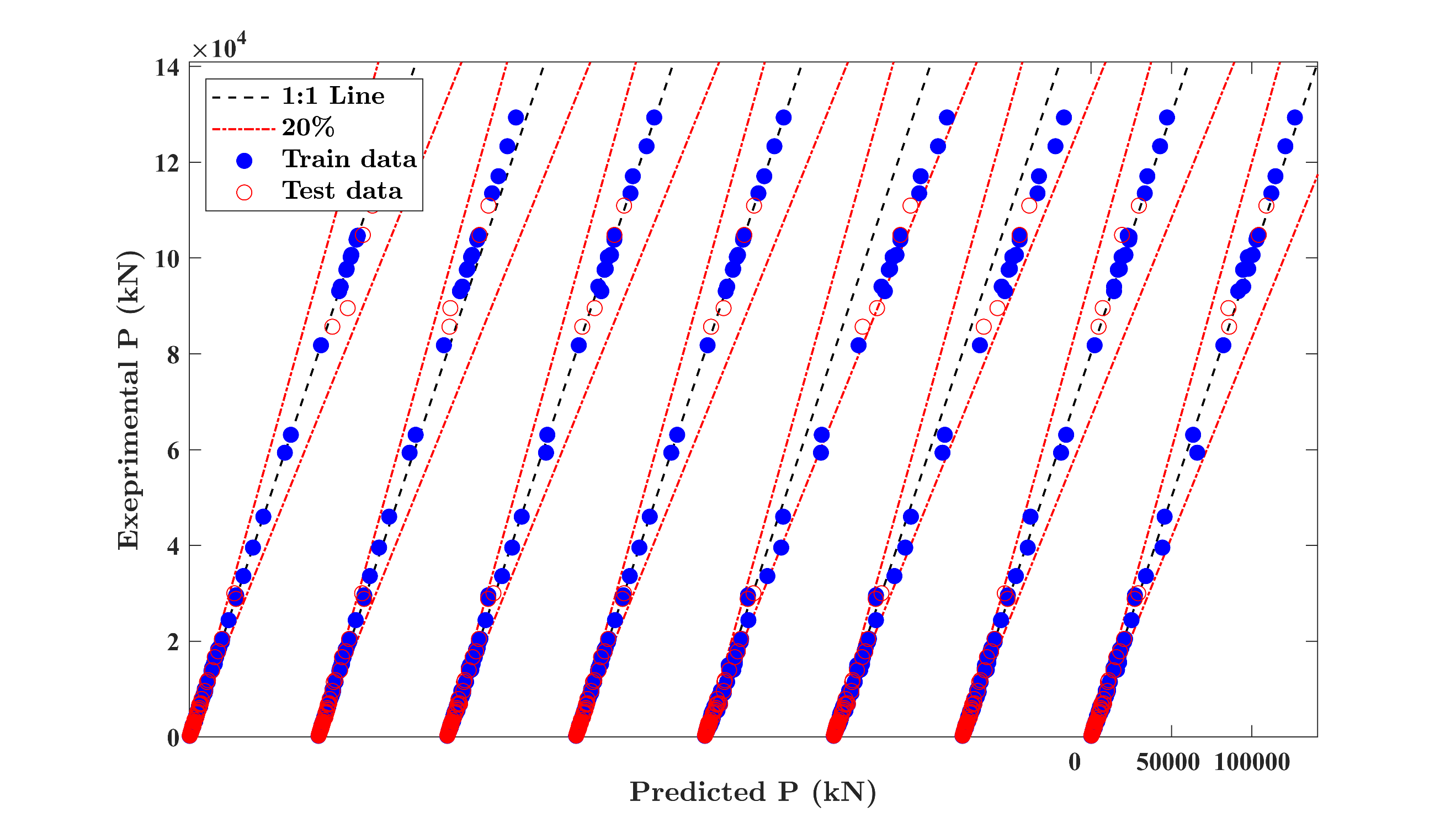


**PSVR**

**GPR**

**Prop. Eqn.**

**RF**

**CATB**

**XGB**

**LGBM**

**ANN**

1. CCFST columns


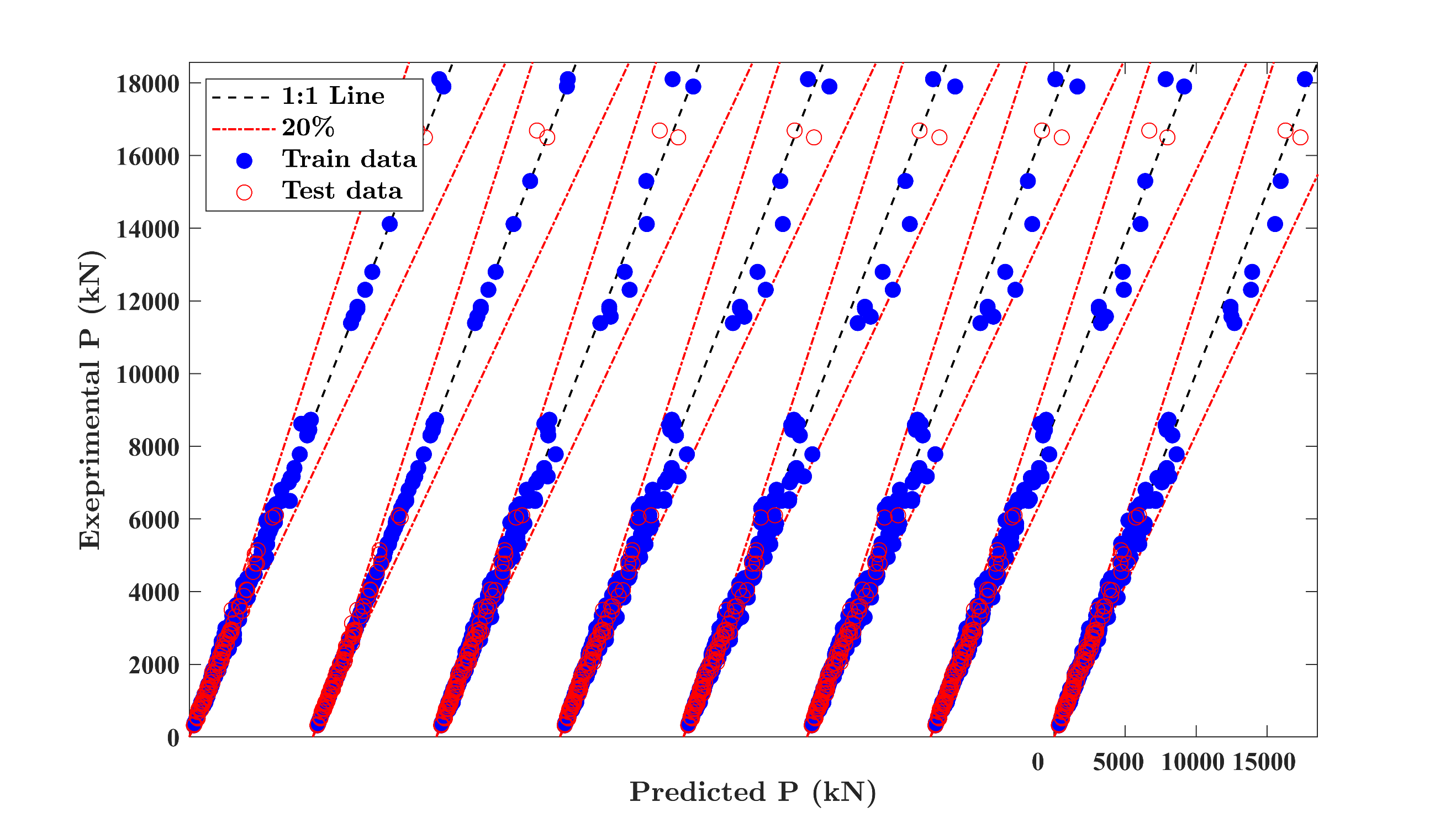


**PSVR**

**GPR**

**Prop. Eqn.**

**RF**

**CATB**

**XGB**

**LGBM**

**ANN**

b) RCFST columns


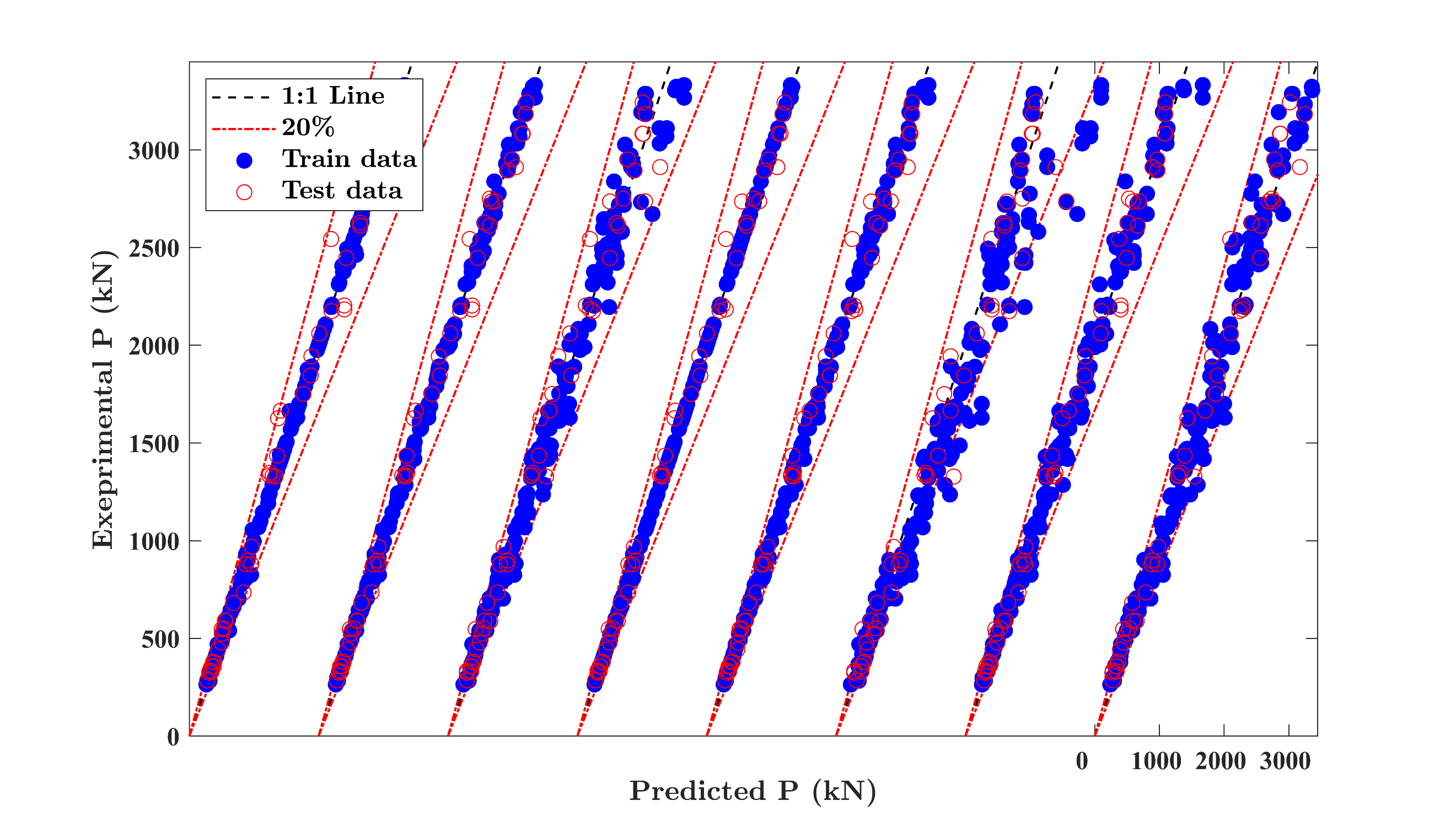


**PSVR**

**GPR**

**Prop. Eqn.**

**RF**

**CATB**

**XGB**

**LGBM**

**ANN**

(c) CFDST columns

Comparison between proposed equations and ML models for training and testing datasets.
