# Supplementary figures and images for: Prediction of the axial compression capacity of stub CFST columns using machine learning techniques
Source: Sci Rep. 2024 Feb 5;14:2885. doi: 10.1038/s41598-024-53352-1 (PMC10838919; doi:10.1038/s41598-024-53352-1)

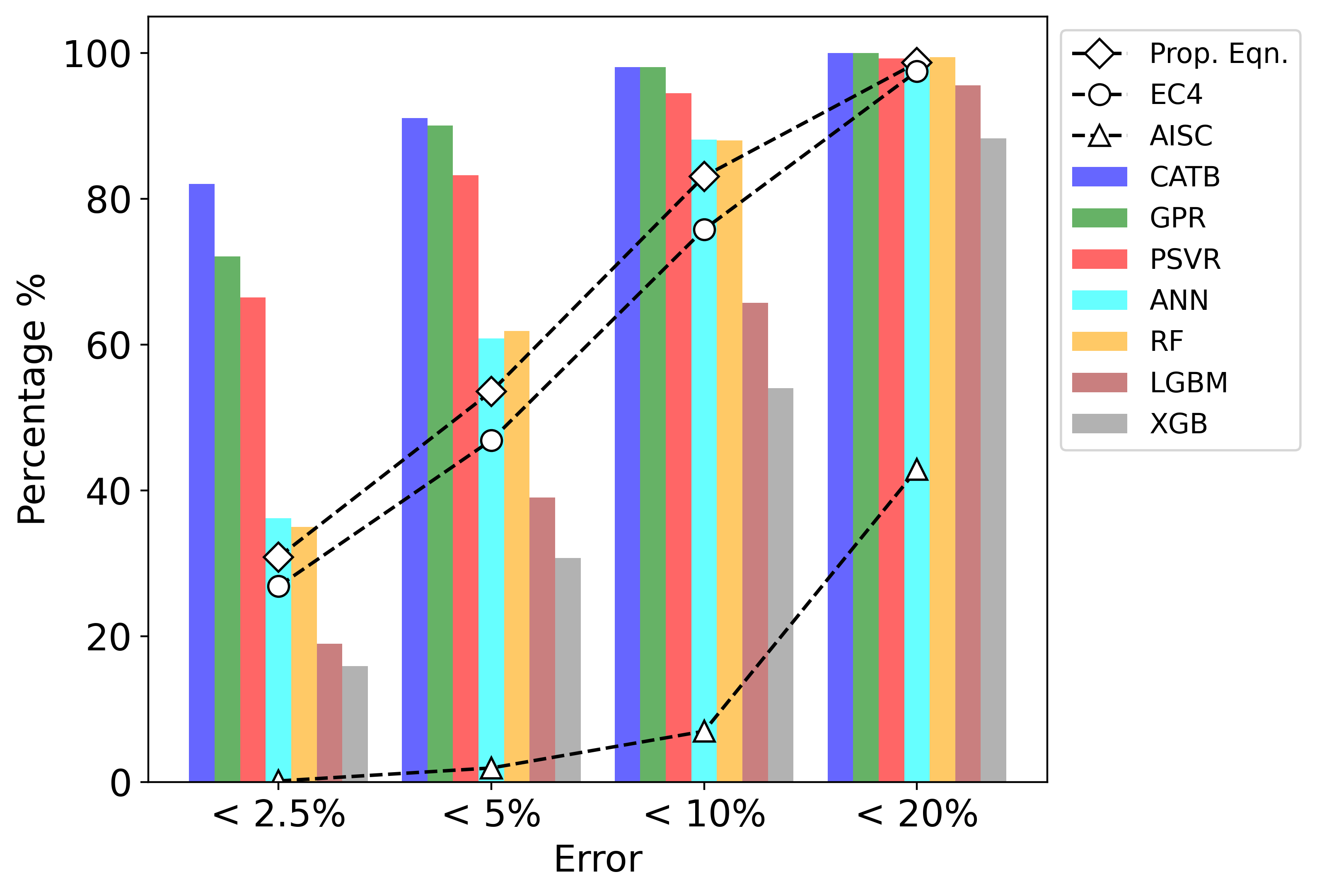

Supplement: Supplementary file 1 — Supplementary Information. [file 41598_2024_53352_MOESM1_ESM.zip › supplementary data/illustration figures/CIRC1.png]

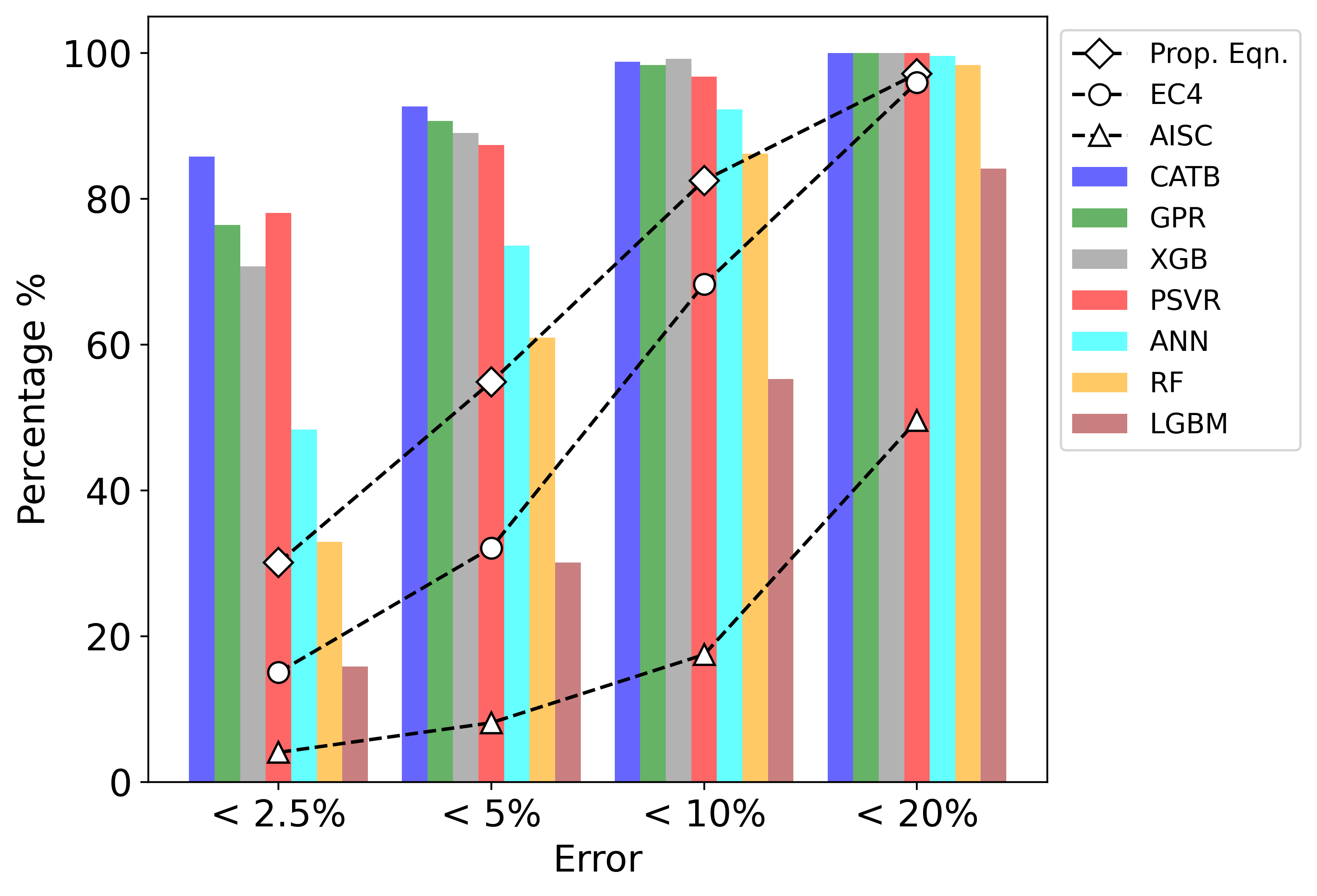

Supplement: Supplementary file 1 — Supplementary Information. [file 41598_2024_53352_MOESM1_ESM.zip › supplementary data/illustration figures/DS1.png]

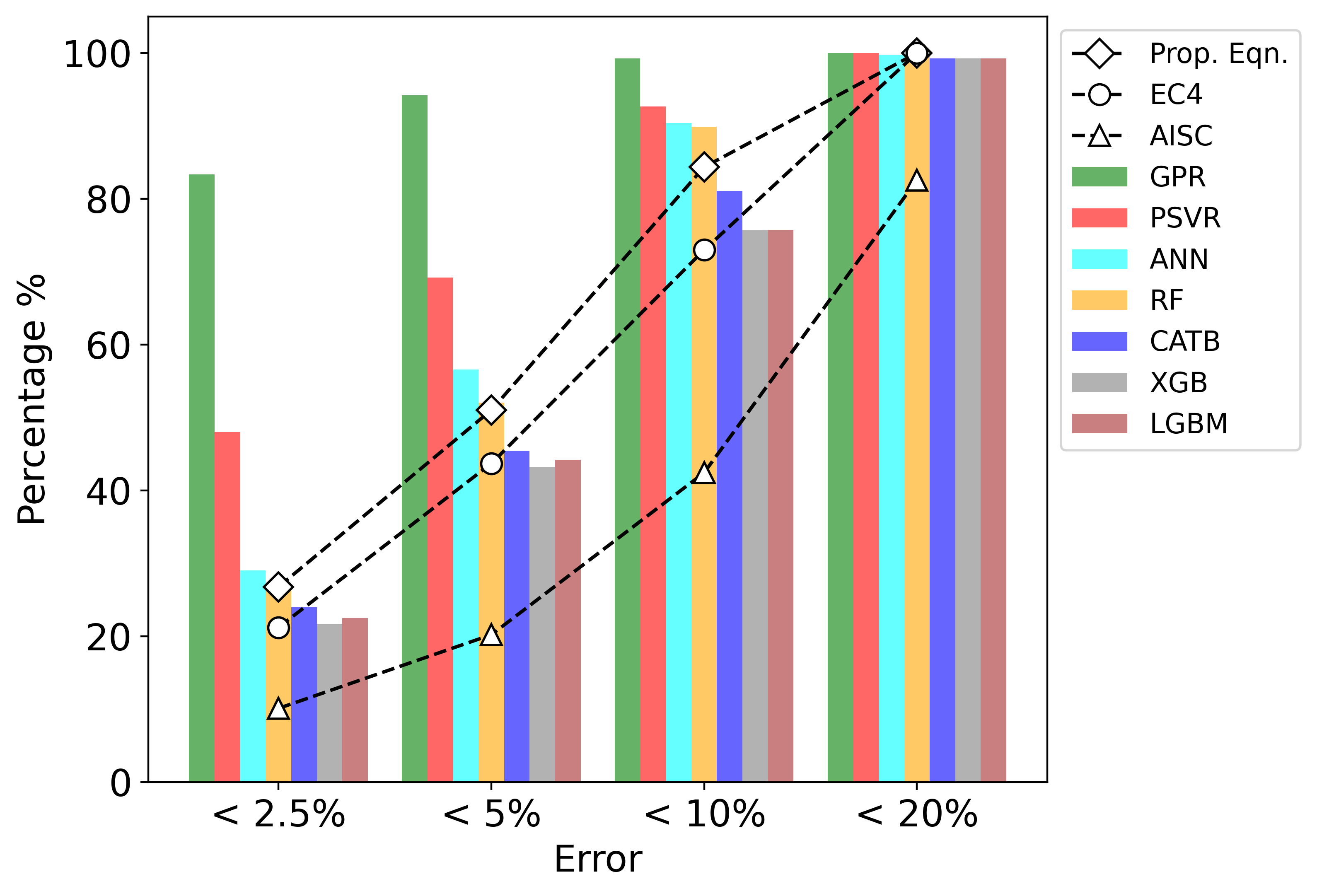

Supplement: Supplementary file 1 — Supplementary Information. [file 41598_2024_53352_MOESM1_ESM.zip › supplementary data/illustration figures/RECT1.png]

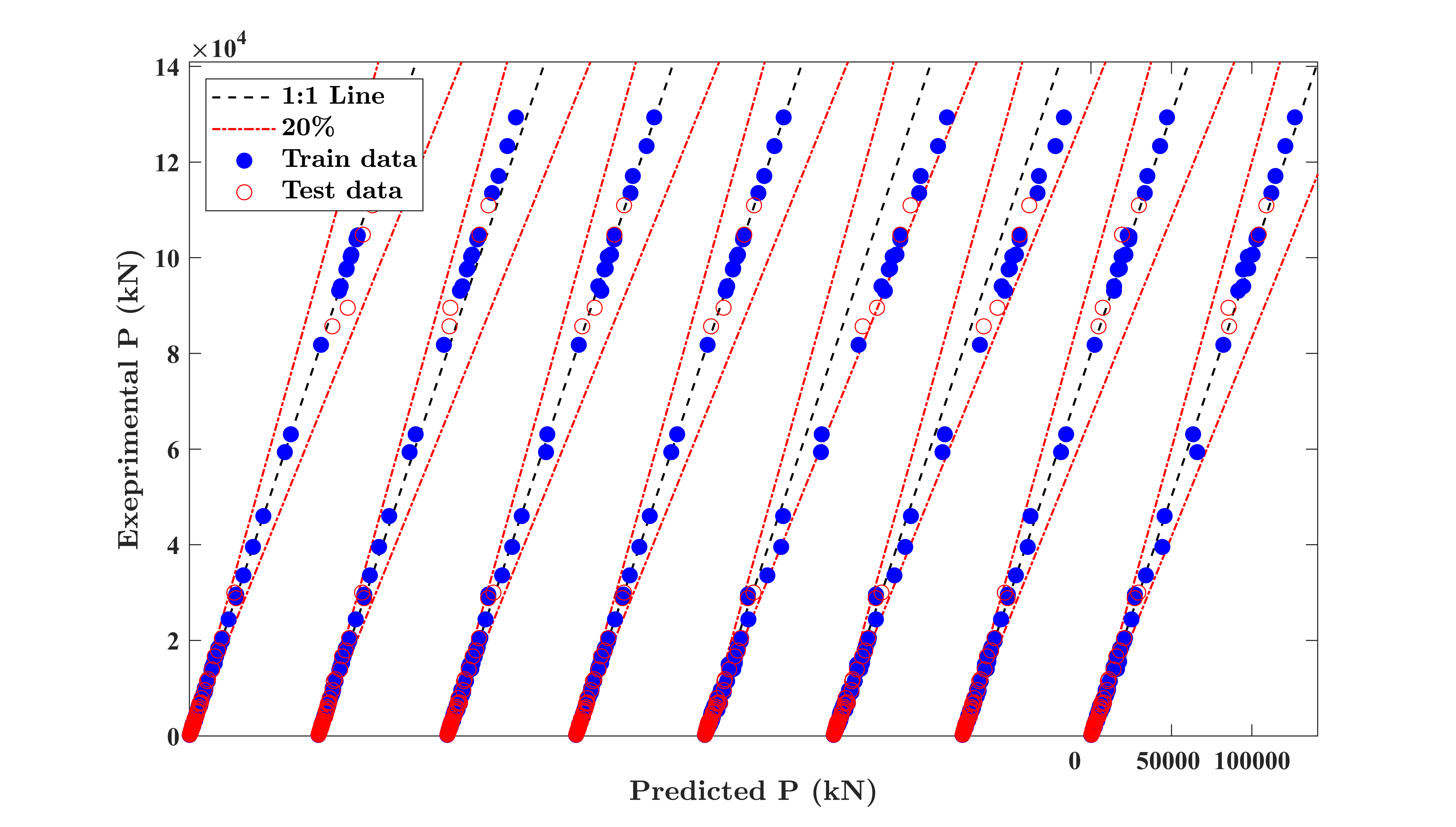

Supplement: Supplementary file 1 — Supplementary Information. [file 41598_2024_53352_MOESM1_ESM.zip › supplementary data/illustration figures/train_test_circ.png]

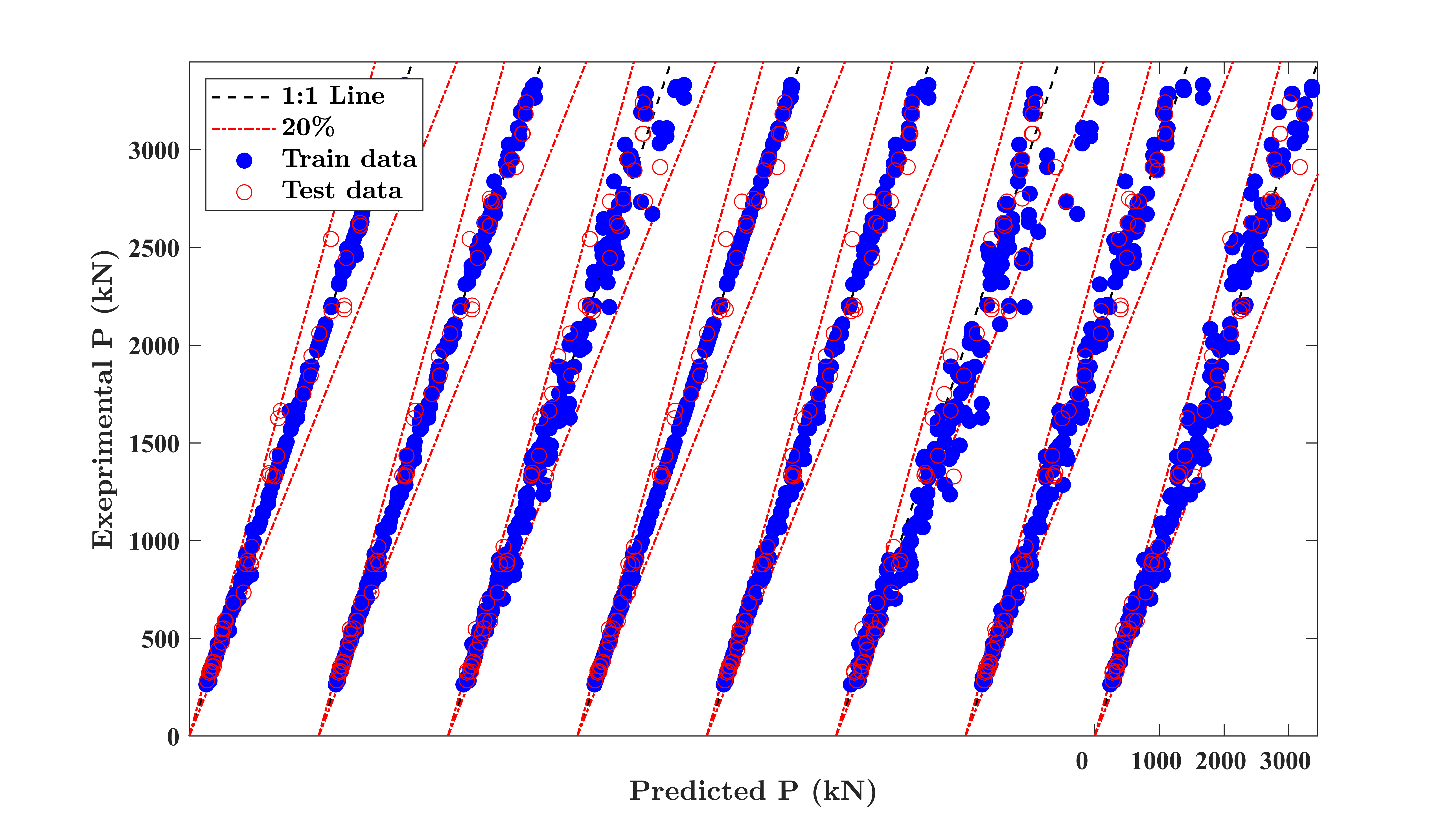

Supplement: Supplementary file 1 — Supplementary Information. [file 41598_2024_53352_MOESM1_ESM.zip › supplementary data/illustration figures/train_test_ds.png]

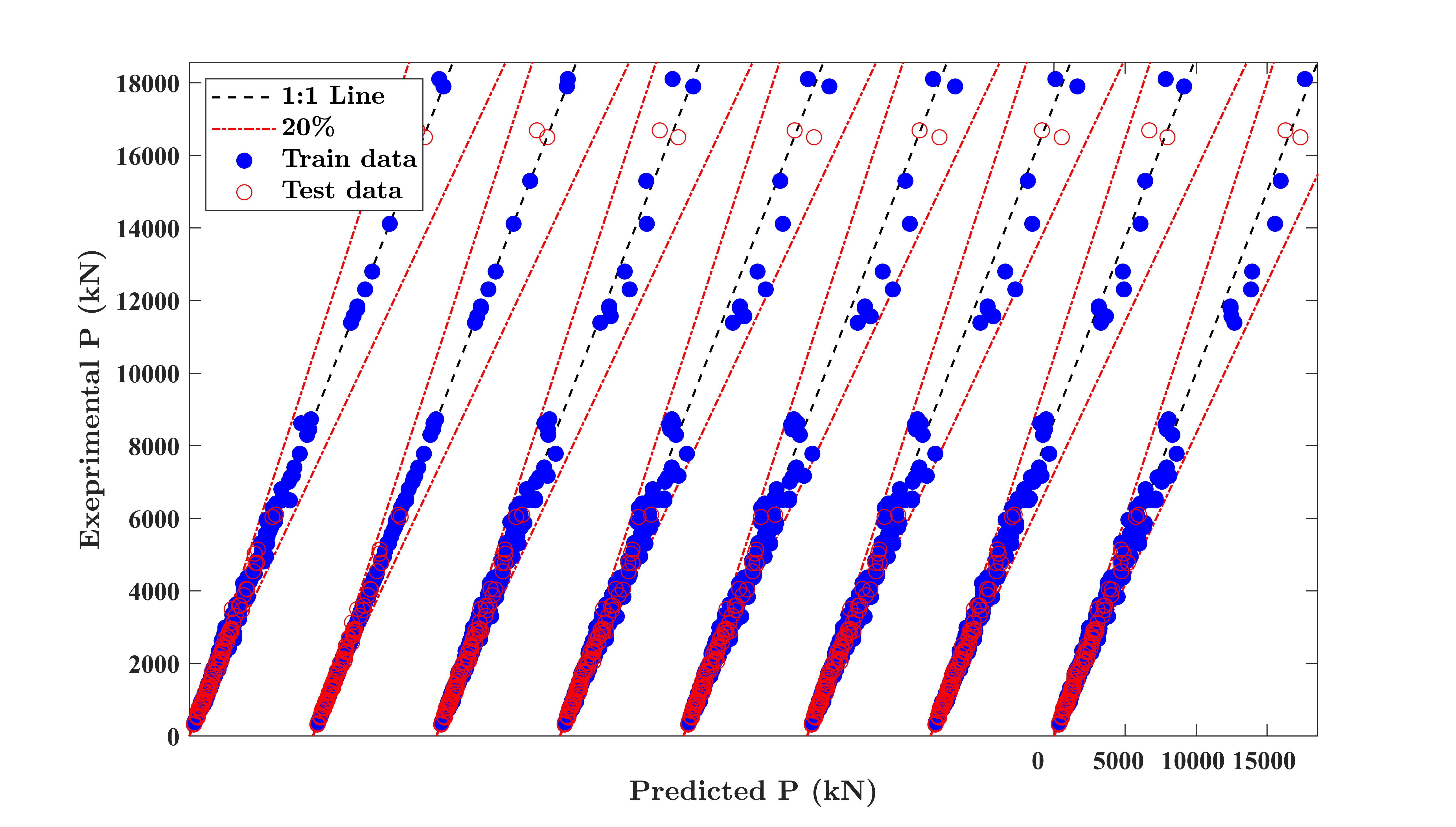

Supplement: Supplementary file 1 — Supplementary Information. [file 41598_2024_53352_MOESM1_ESM.zip › supplementary data/illustration figures/train_test_rec.png]

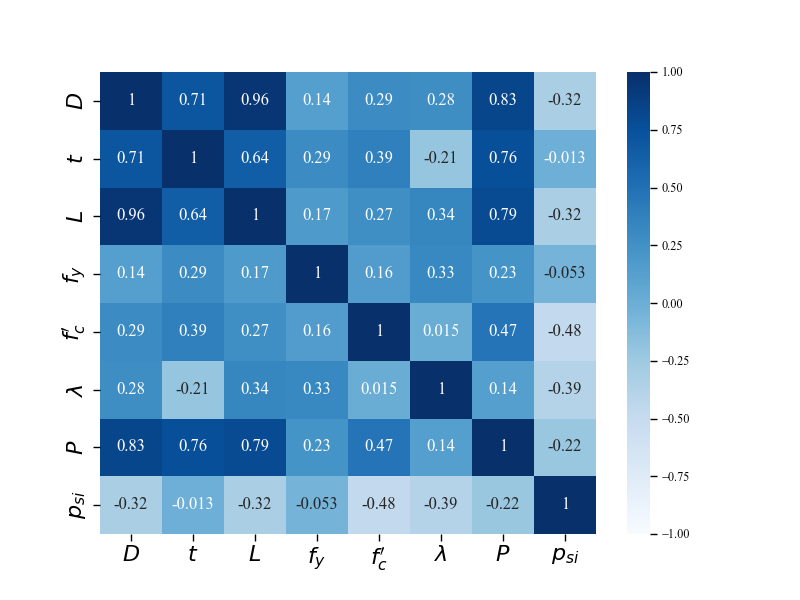

Supplement: Supplementary file 1 — Supplementary Information. [file 41598_2024_53352_MOESM1_ESM.zip › supplementary data/illustration figures/Correlation and distribution/correlation_circ.png]

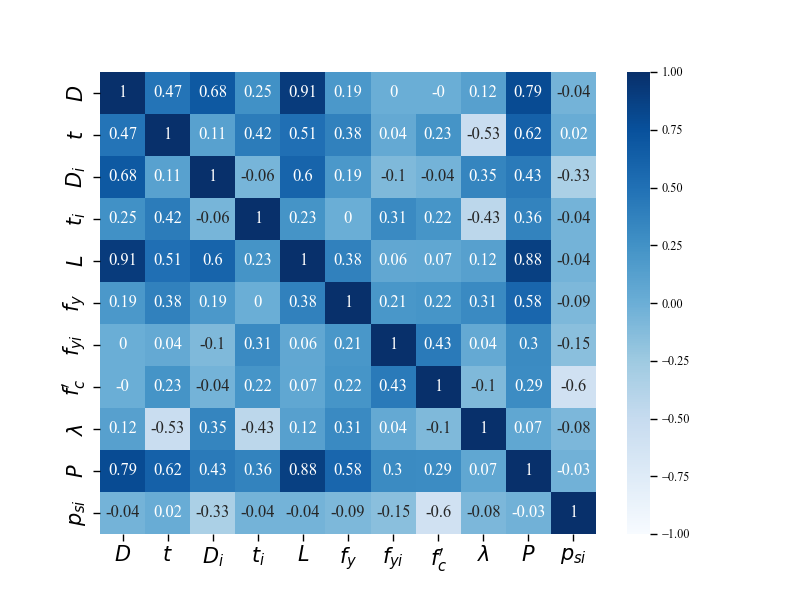

Supplement: Supplementary file 1 — Supplementary Information. [file 41598_2024_53352_MOESM1_ESM.zip › supplementary data/illustration figures/Correlation and distribution/correlation_DS.png]

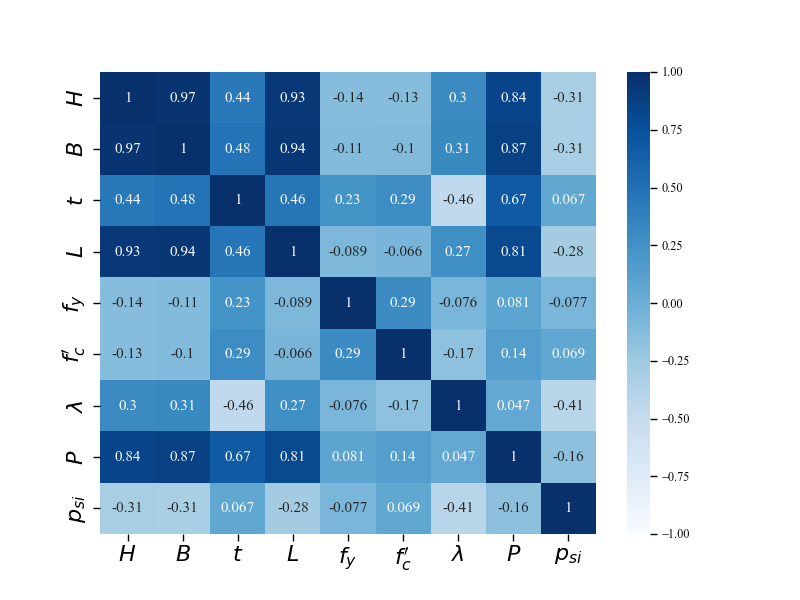

Supplement: Supplementary file 1 — Supplementary Information. [file 41598_2024_53352_MOESM1_ESM.zip › supplementary data/illustration figures/Correlation and distribution/correlation_rect.png]

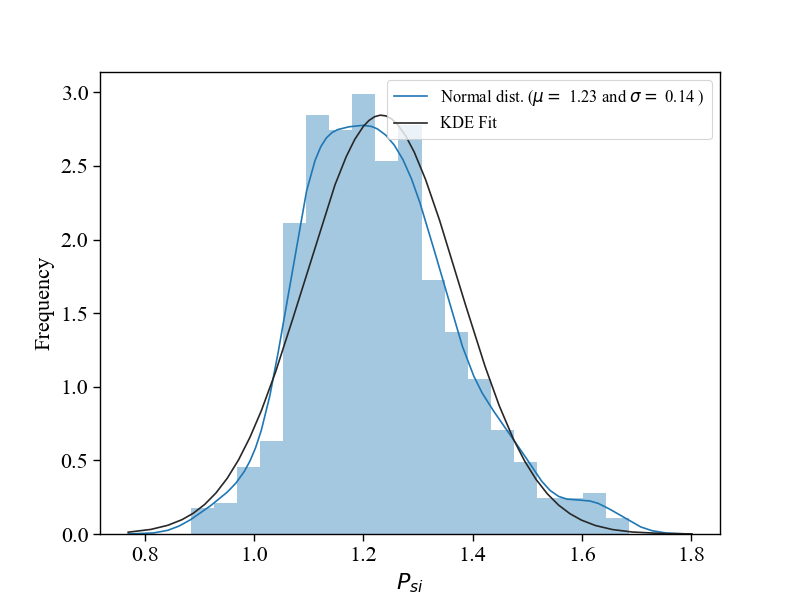

Supplement: Supplementary file 1 — Supplementary Information. [file 41598_2024_53352_MOESM1_ESM.zip › supplementary data/illustration figures/Correlation and distribution/Distribution_circ.png]

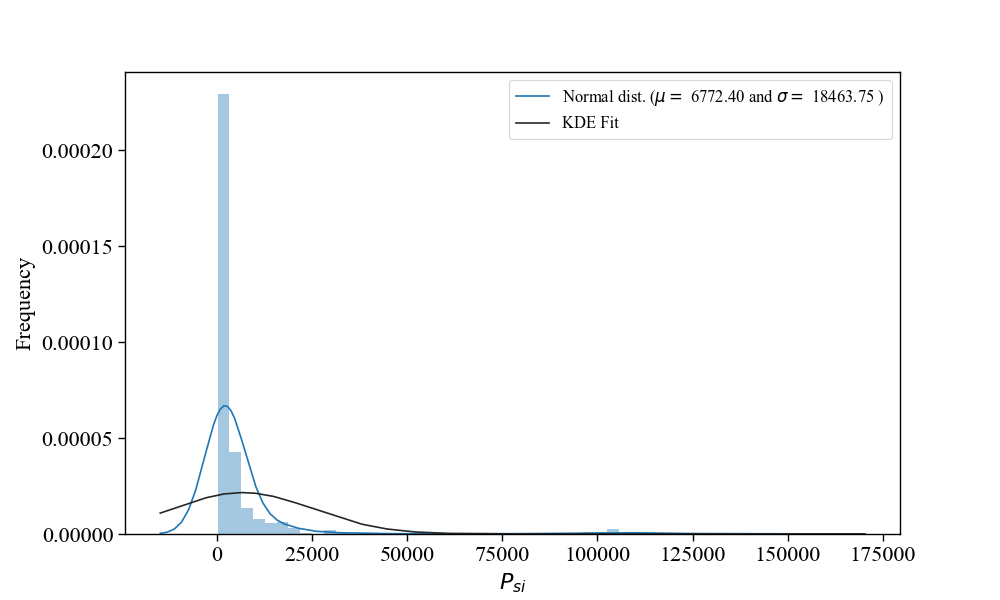

Supplement: Supplementary file 1 — Supplementary Information. [file 41598_2024_53352_MOESM1_ESM.zip › supplementary data/illustration figures/Correlation and distribution/Distribution_circ_before.png]

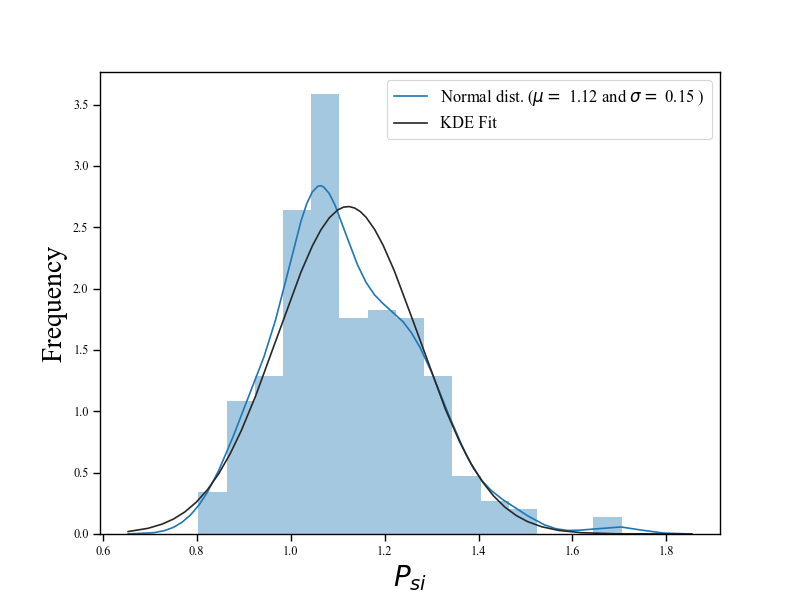

Supplement: Supplementary file 1 — Supplementary Information. [file 41598_2024_53352_MOESM1_ESM.zip › supplementary data/illustration figures/Correlation and distribution/Distribution_DS.png]

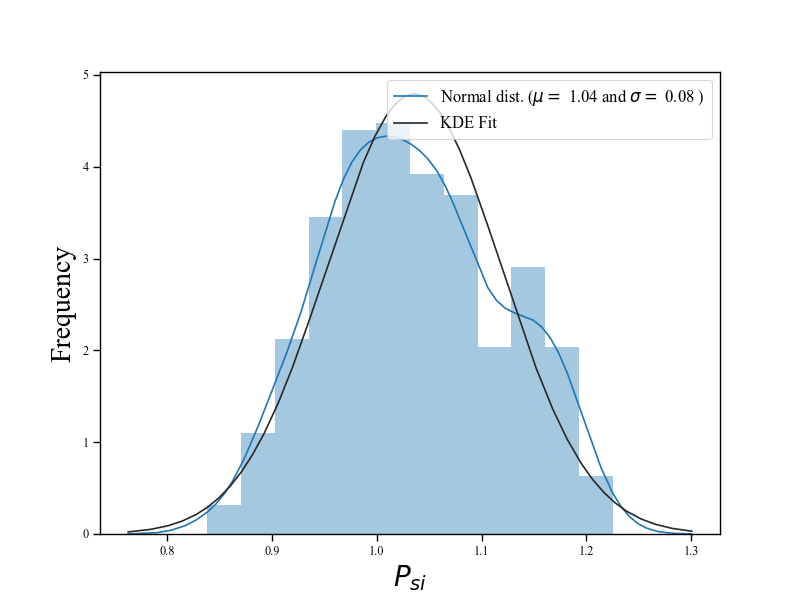

Supplement: Supplementary file 1 — Supplementary Information. [file 41598_2024_53352_MOESM1_ESM.zip › supplementary data/illustration figures/Correlation and distribution/Distribution_rect.png]

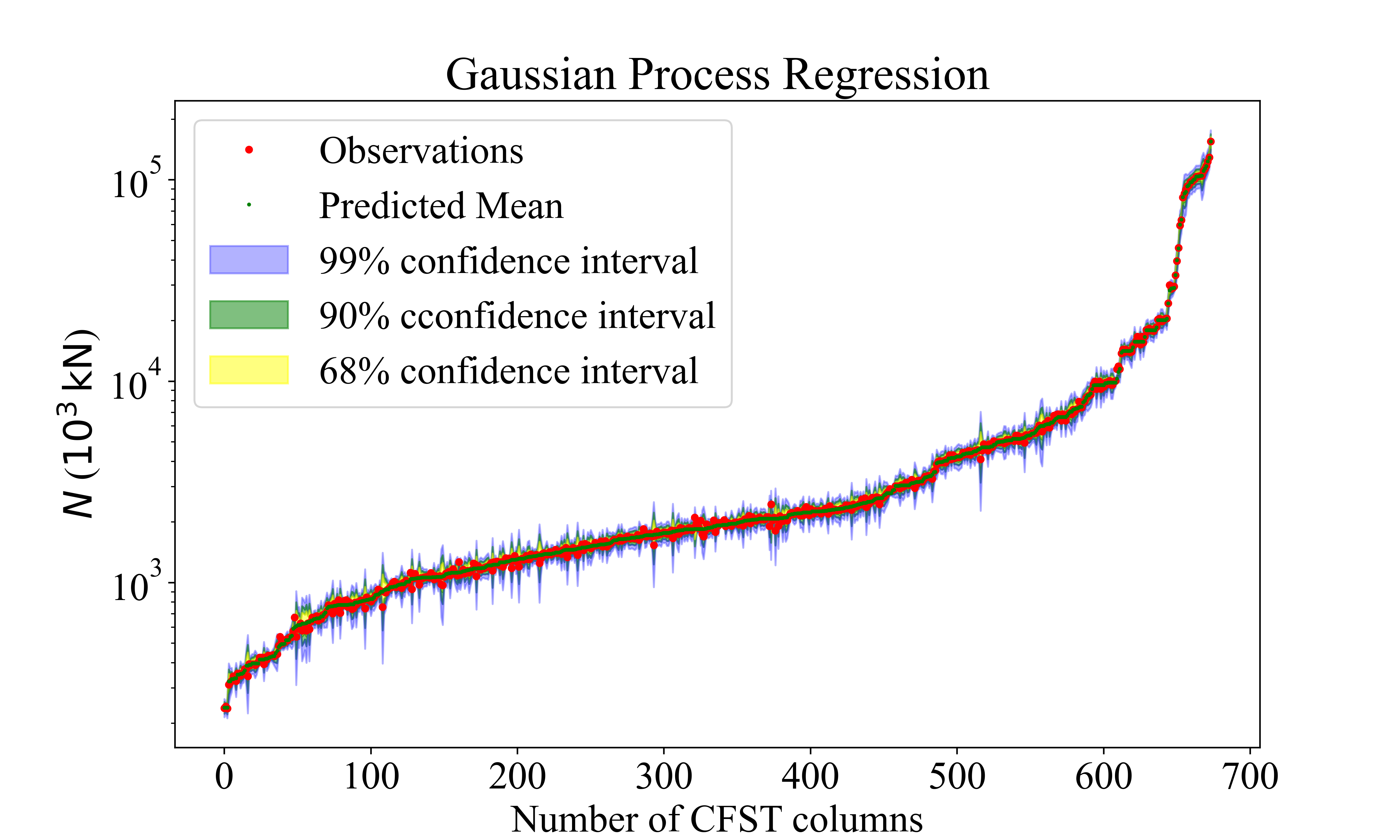

Supplement: Supplementary file 1 — Supplementary Information. [file 41598_2024_53352_MOESM1_ESM.zip › supplementary data/illustration figures/GPR/GPR_Predicted_Mean_Confidence_Intervals1.png]

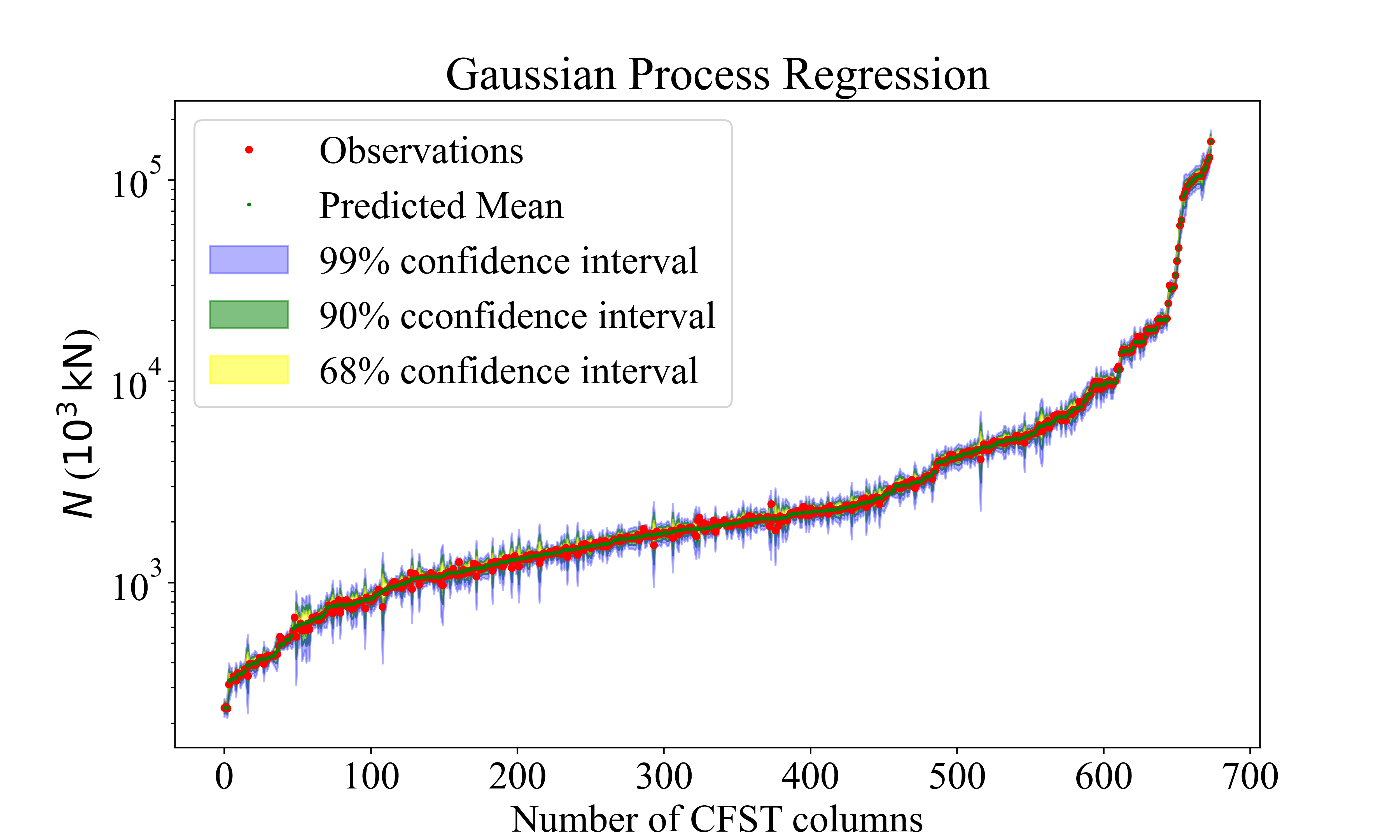

Supplement: Supplementary file 1 — Supplementary Information. [file 41598_2024_53352_MOESM1_ESM.zip › supplementary data/illustration figures/GPR/GPR_Predicted_Mean_Confidence_Intervals1i.png]

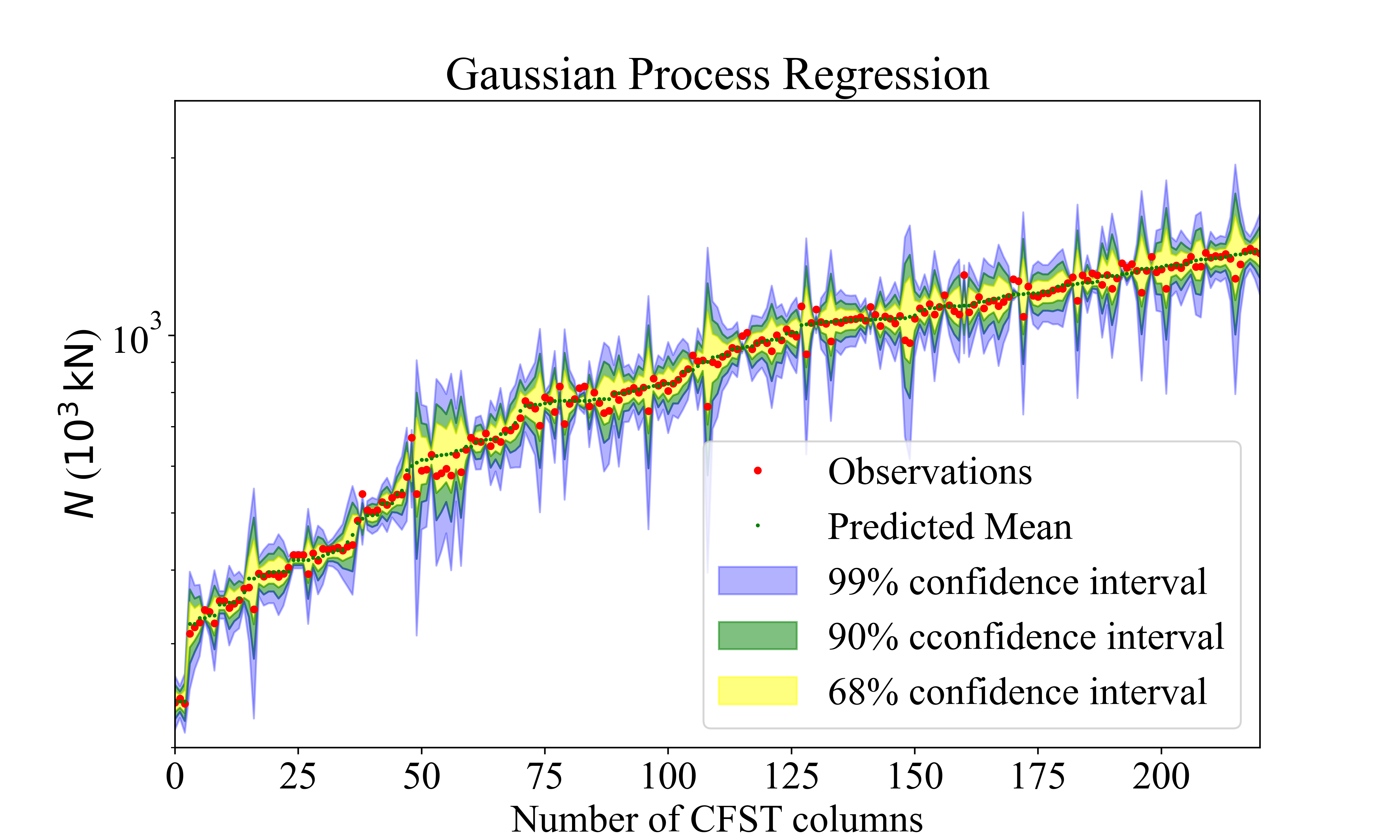

Supplement: Supplementary file 1 — Supplementary Information. [file 41598_2024_53352_MOESM1_ESM.zip › supplementary data/illustration figures/GPR/GPR_Predicted_Mean_Confidence_Intervals1i11.png]

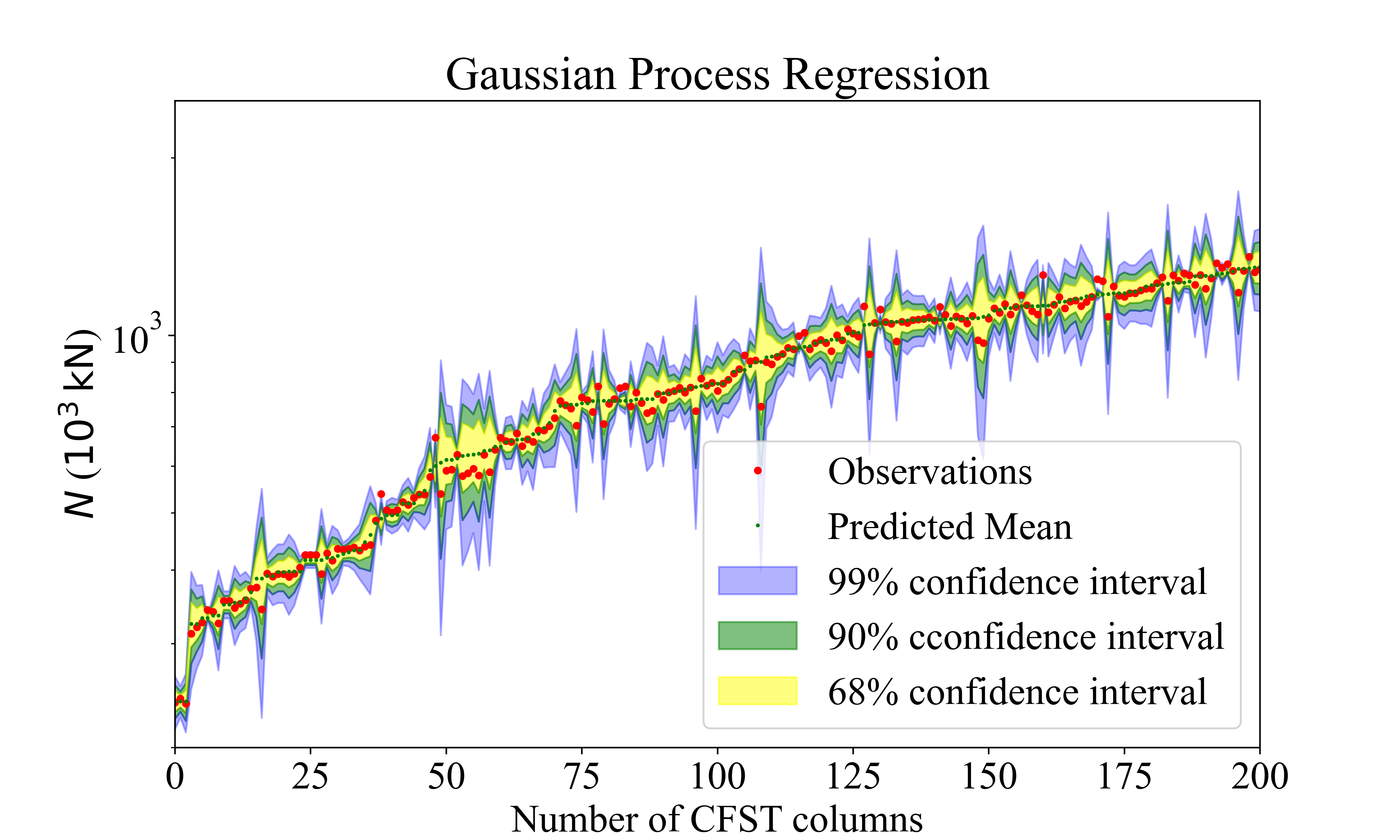

Supplement: Supplementary file 1 — Supplementary Information. [file 41598_2024_53352_MOESM1_ESM.zip › supplementary data/illustration figures/GPR/GPR_Predicted_Mean_Confidence_Intervals2.png]

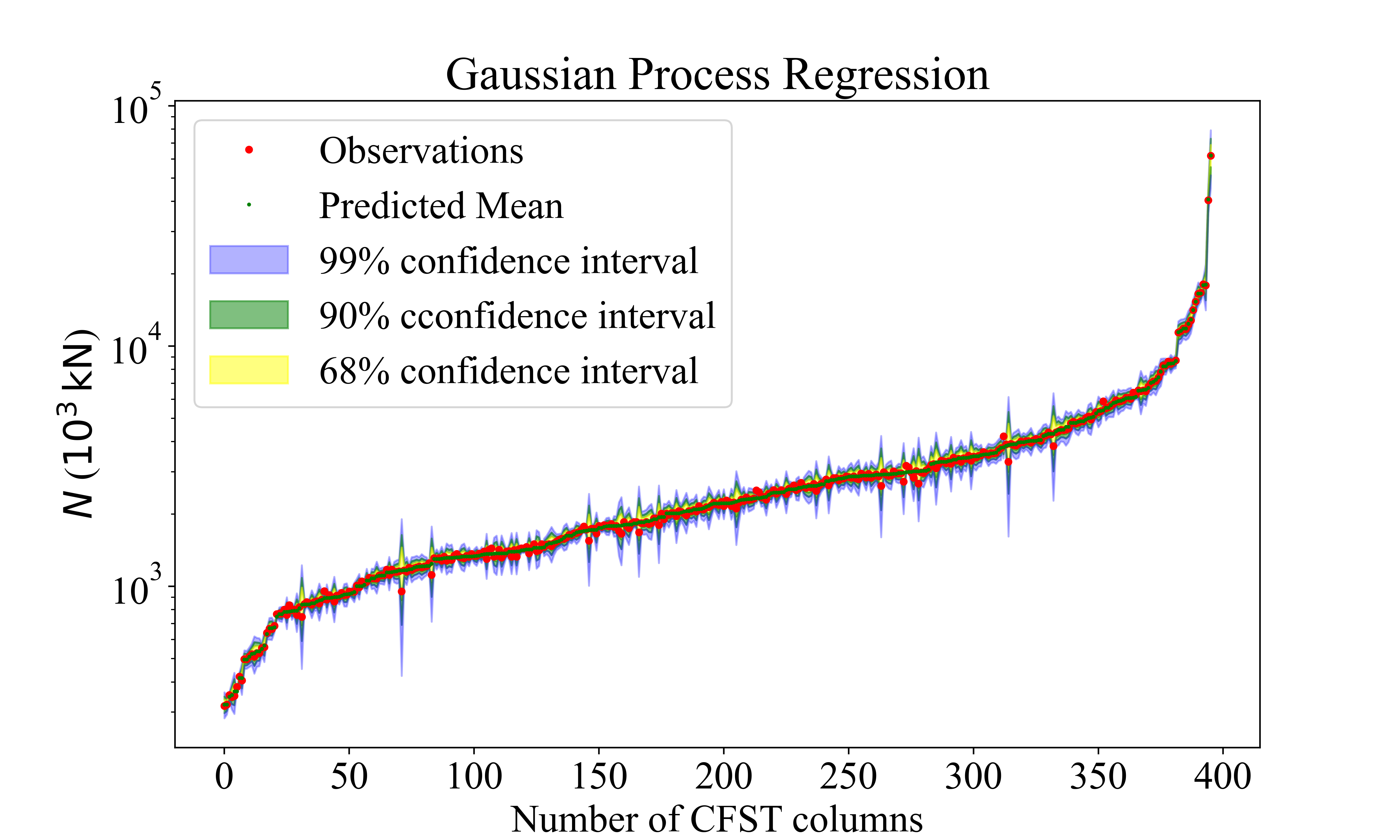

Supplement: Supplementary file 1 — Supplementary Information. [file 41598_2024_53352_MOESM1_ESM.zip › supplementary data/illustration figures/GPR/Rect_GPR_Predicted_Mean_Confidence_Intervals1.png]

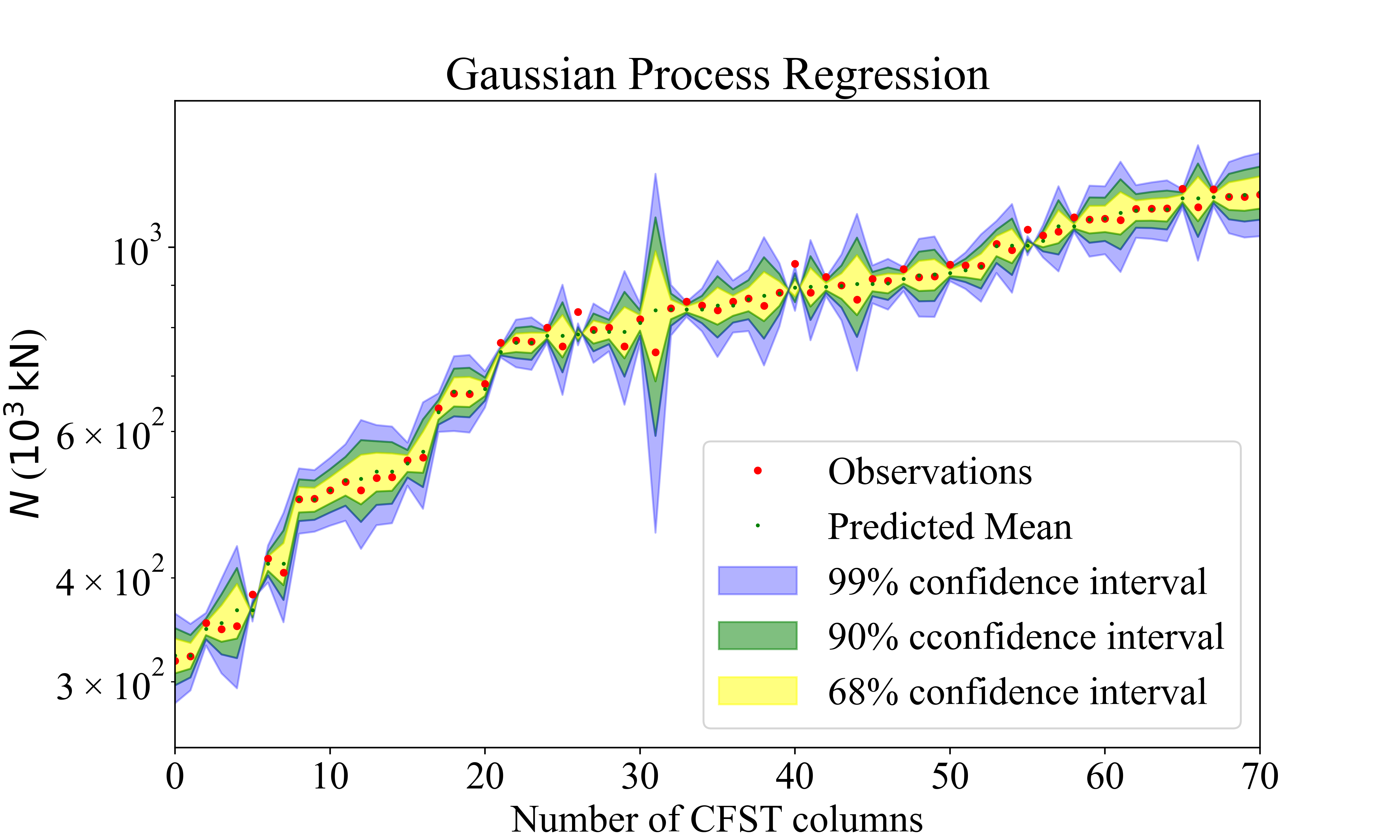

Supplement: Supplementary file 1 — Supplementary Information. [file 41598_2024_53352_MOESM1_ESM.zip › supplementary data/illustration figures/GPR/Rect_GPR_Predicted_Mean_Confidence_Intervals2.png]

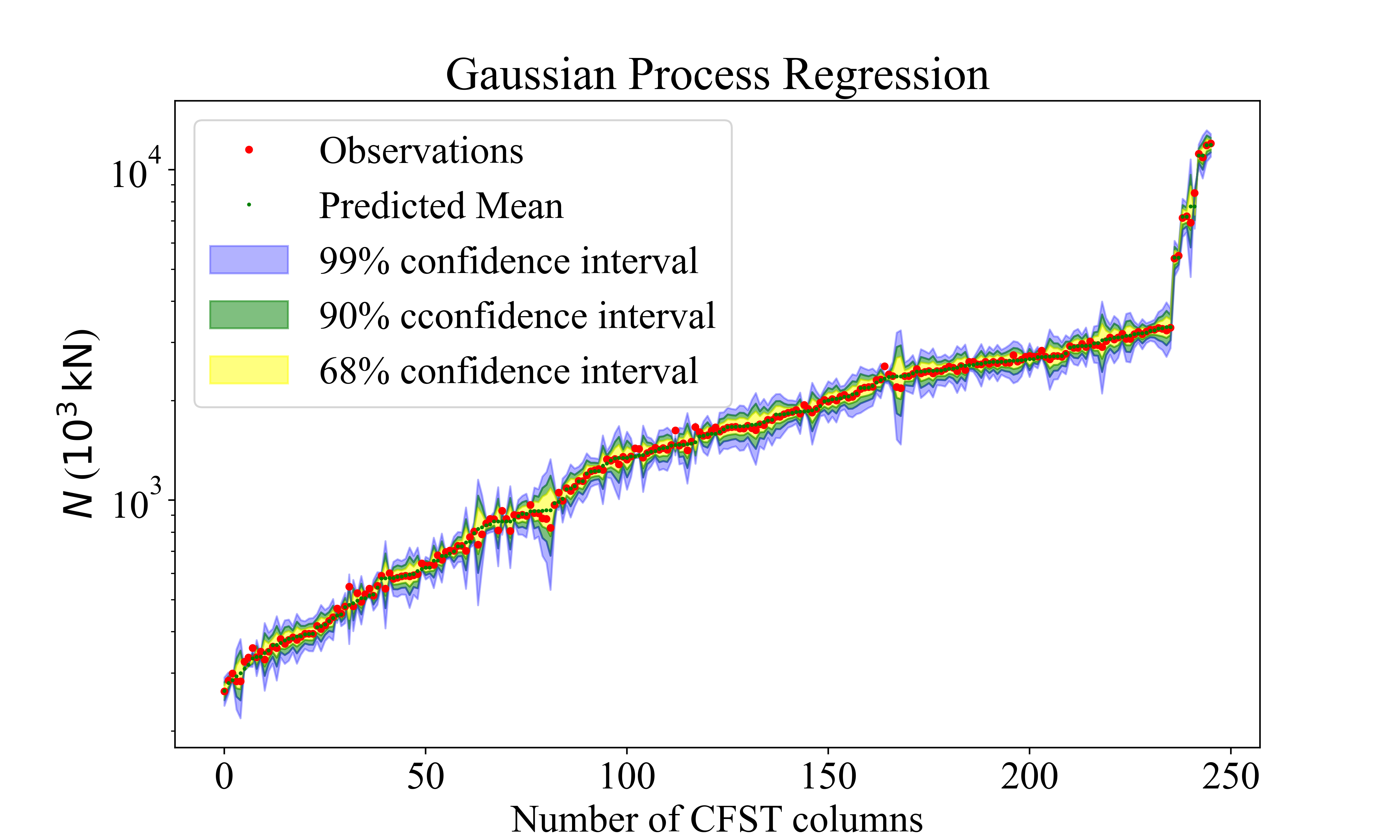

Supplement: Supplementary file 1 — Supplementary Information. [file 41598_2024_53352_MOESM1_ESM.zip › supplementary data/illustration figures/GPR/Skin_GPR_Predicted_Mean_Confidence_Intervals1.png]

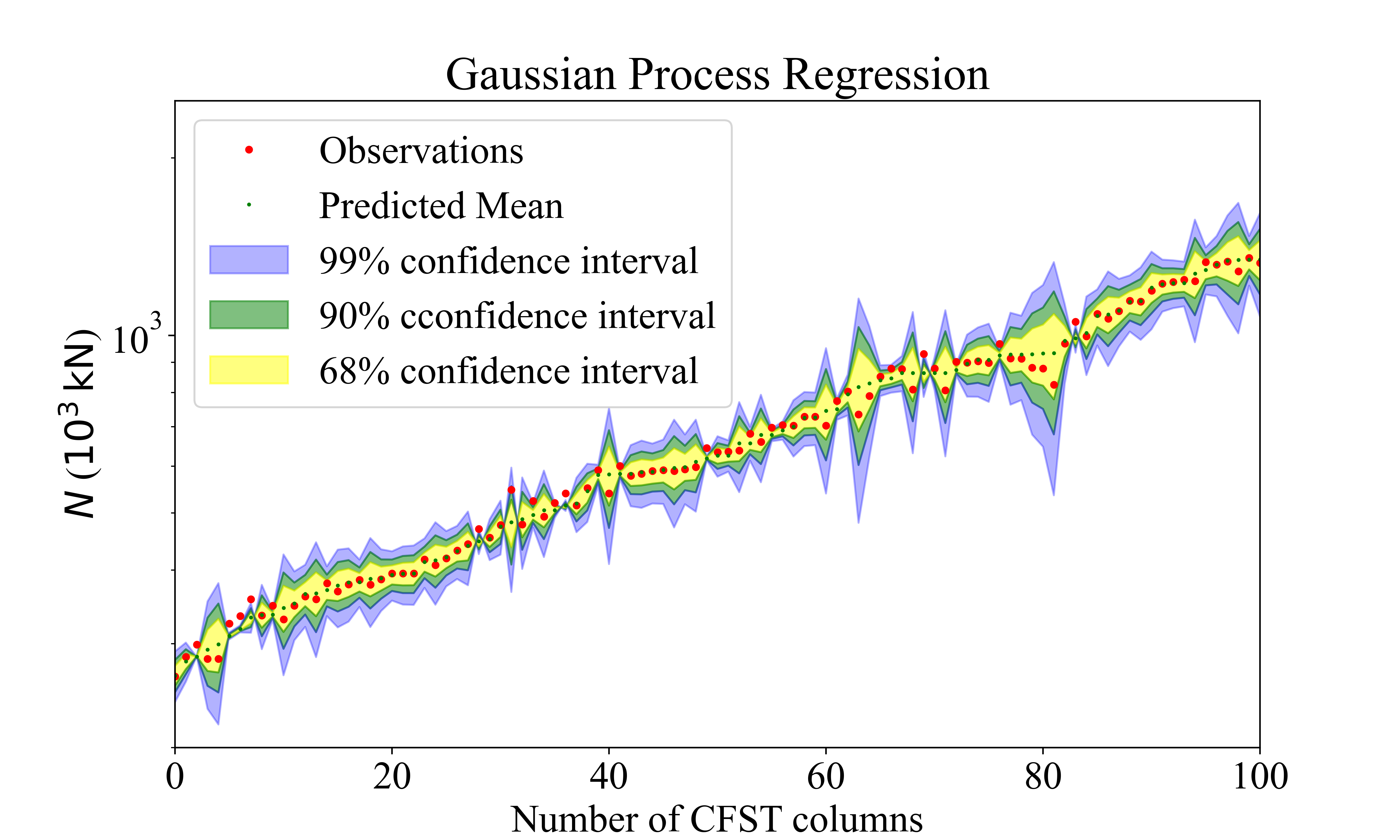

Supplement: Supplementary file 1 — Supplementary Information. [file 41598_2024_53352_MOESM1_ESM.zip › supplementary data/illustration figures/GPR/Skin_GPR_Predicted_Mean_Confidence_Intervals2.png]

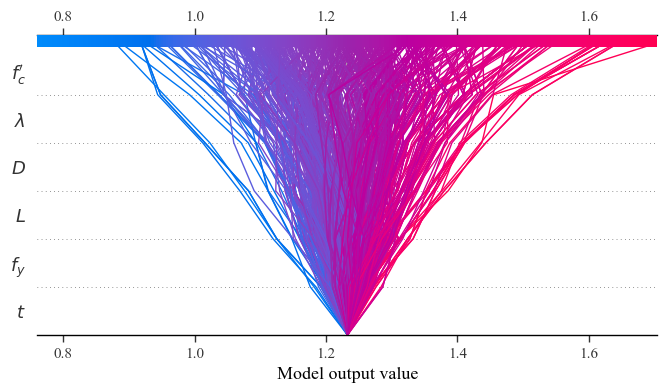

Supplement: Supplementary file 1 — Supplementary Information. [file 41598_2024_53352_MOESM1_ESM.zip › supplementary data/illustration figures/shap analysis/decision_plot_circ.png]

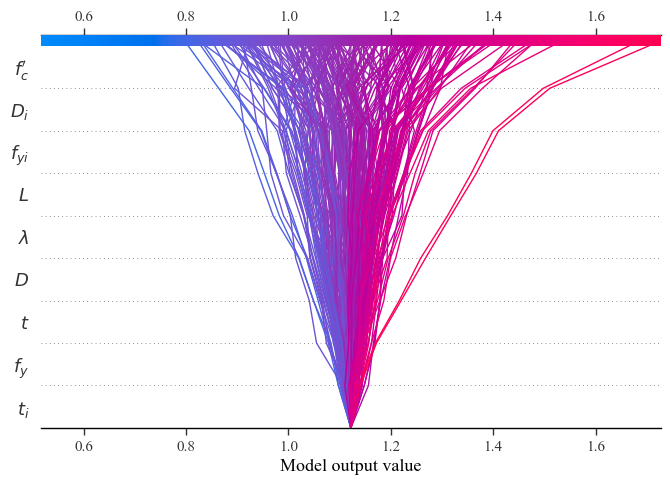

Supplement: Supplementary file 1 — Supplementary Information. [file 41598_2024_53352_MOESM1_ESM.zip › supplementary data/illustration figures/shap analysis/decision_plot_ds.png]

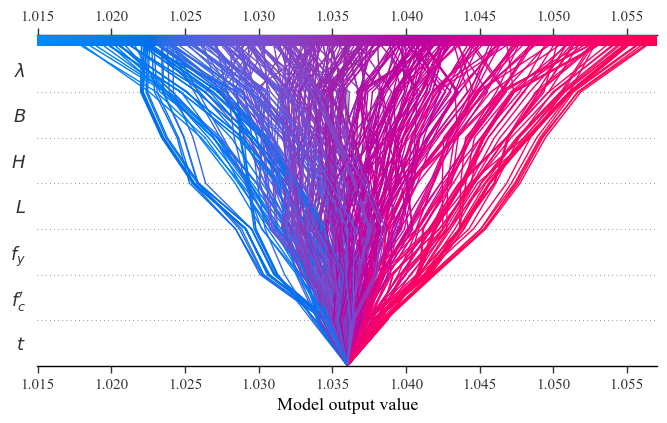

Supplement: Supplementary file 1 — Supplementary Information. [file 41598_2024_53352_MOESM1_ESM.zip › supplementary data/illustration figures/shap analysis/decision_plot_rect.png]

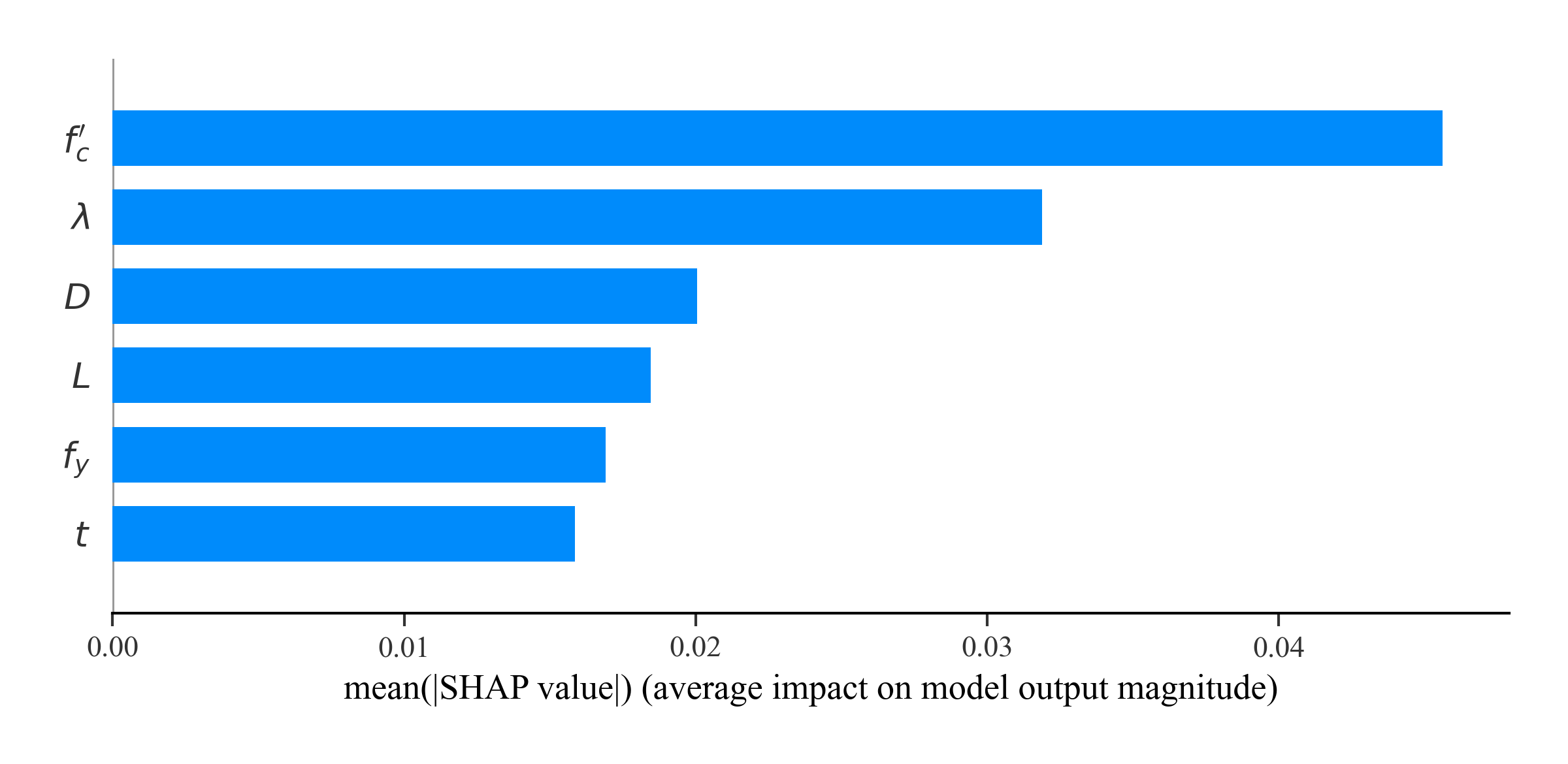

Supplement: Supplementary file 1 — Supplementary Information. [file 41598_2024_53352_MOESM1_ESM.zip › supplementary data/illustration figures/shap analysis/shap_summary_plot_circ.png]

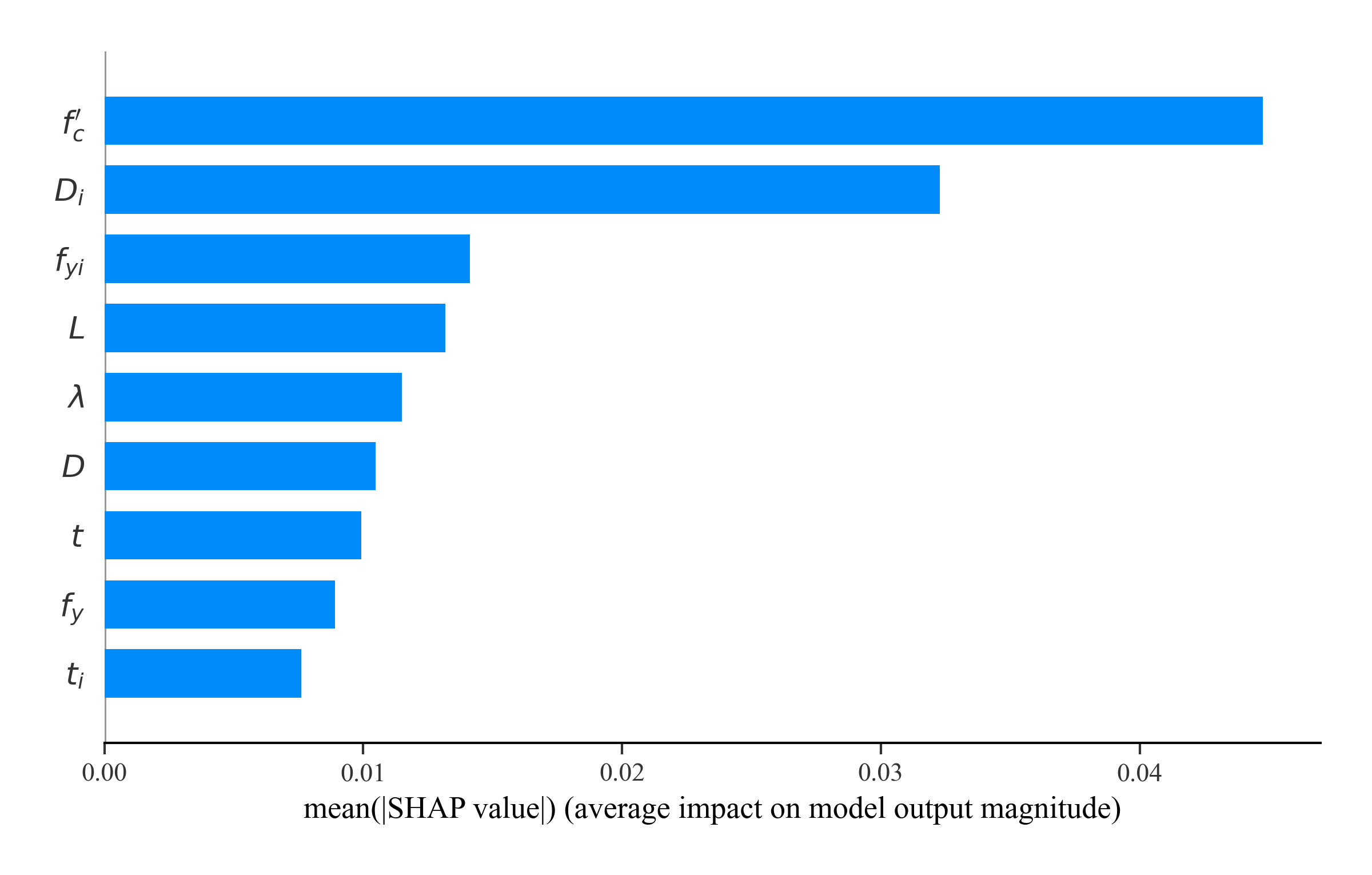

Supplement: Supplementary file 1 — Supplementary Information. [file 41598_2024_53352_MOESM1_ESM.zip › supplementary data/illustration figures/shap analysis/shap_summary_plot_ds.png]

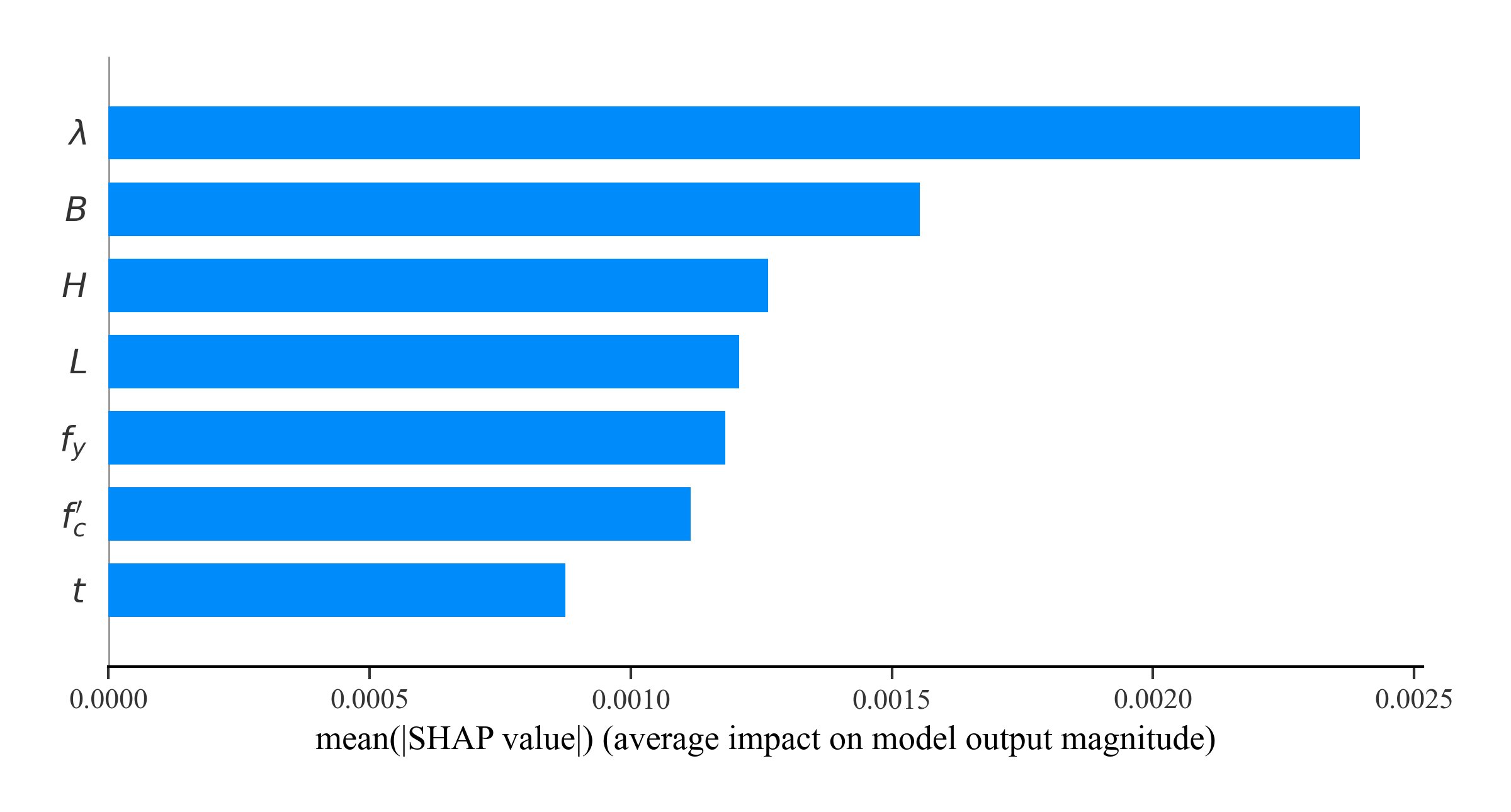

Supplement: Supplementary file 1 — Supplementary Information. [file 41598_2024_53352_MOESM1_ESM.zip › supplementary data/illustration figures/shap analysis/shap_summary_plot_rect.png]

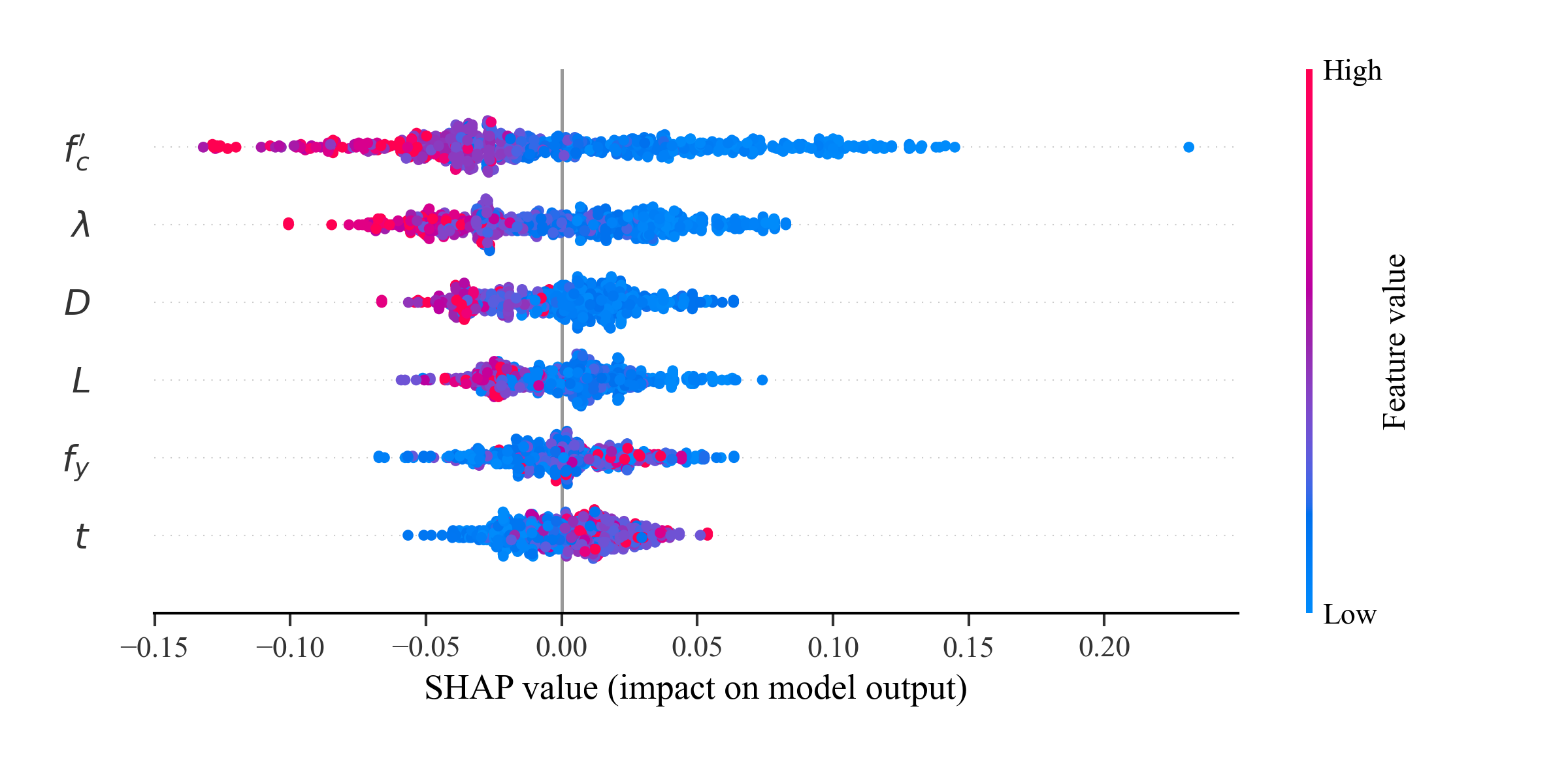

Supplement: Supplementary file 1 — Supplementary Information. [file 41598_2024_53352_MOESM1_ESM.zip › supplementary data/illustration figures/shap analysis/summary_plot_circ_short.png]

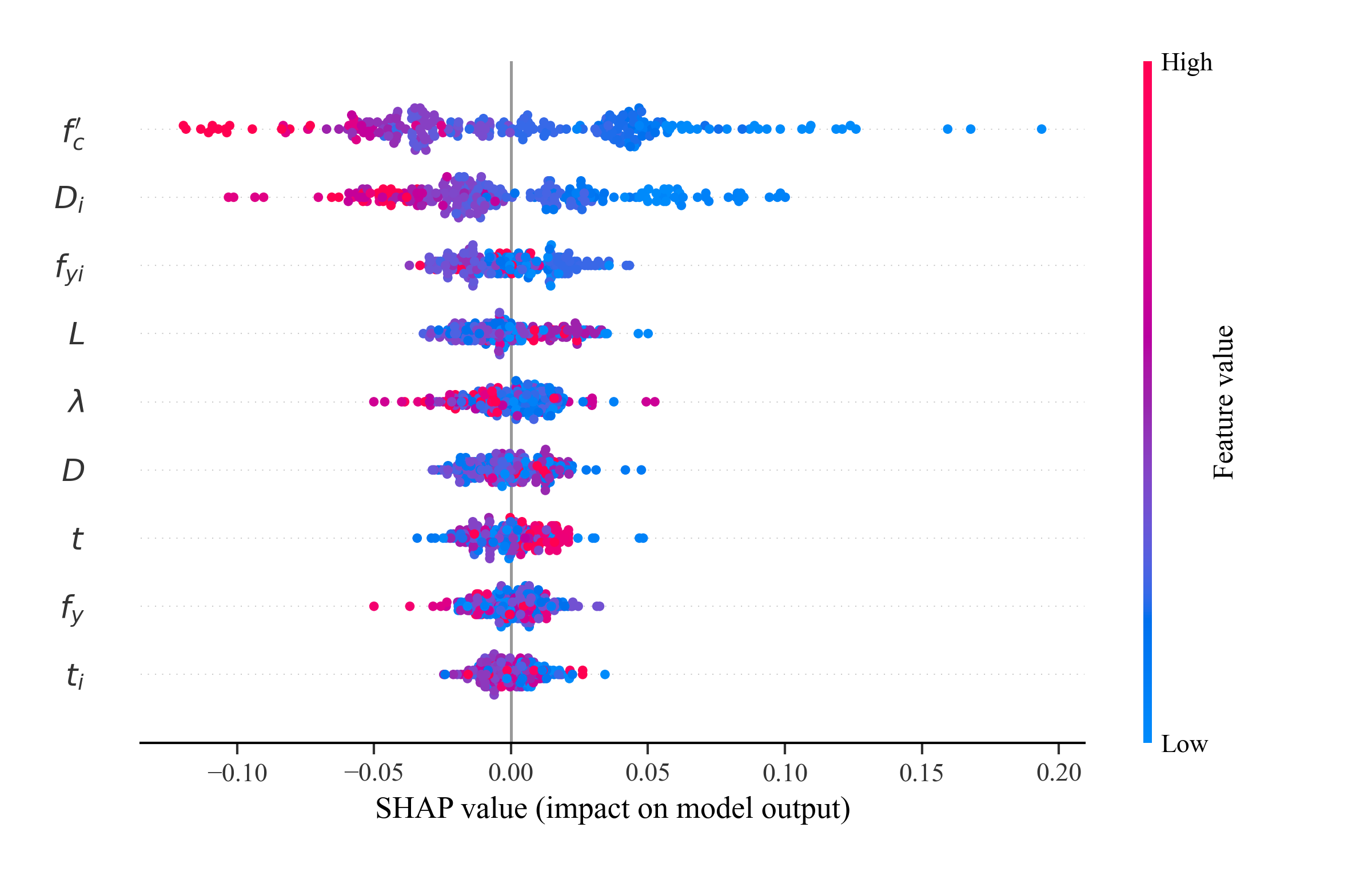

Supplement: Supplementary file 1 — Supplementary Information. [file 41598_2024_53352_MOESM1_ESM.zip › supplementary data/illustration figures/shap analysis/summary_plot_ds_short.png]

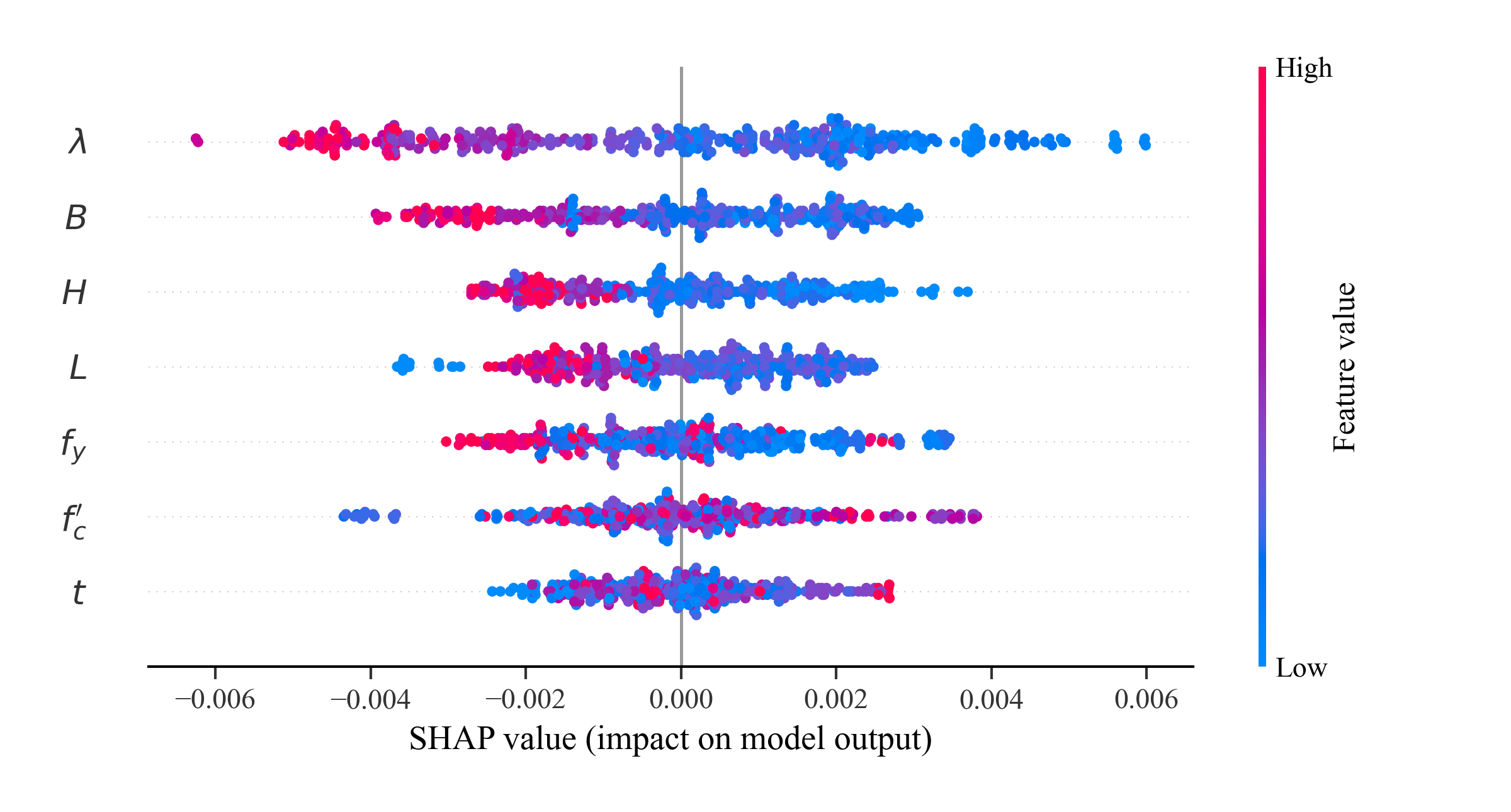

Supplement: Supplementary file 1 — Supplementary Information. [file 41598_2024_53352_MOESM1_ESM.zip › supplementary data/illustration figures/shap analysis/summary_plot_rect_short.png]
